# Occupational health in the Gulf Cooperation Council (GCC): A systematic review and call for comprehensive policy development

**DOI:** 10.1371/journal.pone.0312251

**Published:** 2024-12-10

**Authors:** Muhammad A. Masood, Raghad Khaled, Ahmad Bin-Ismail, Lucy Semerjian, Khaled Abass

**Affiliations:** 1 Department of Environmental Health Sciences, College of Health Sciences, University of Sharjah, Sharjah, United Arab Emirates; 2 Research Institute of Science and Engineering, University of Sharjah, Sharjah, United Arab Emirates; 3 Research Institute for Medical and Health Sciences, University of Sharjah, Sharjah, United Arab Emirates; 4 Research Unit of Biomedicine and Internal Medicine, University of Oulu, Oulu, Finland; University of the West of England, UNITED KINGDOM OF GREAT BRITAIN AND NORTHERN IRELAND

## Abstract

**Background:**

This systematic review evaluates occupational health within the Gulf Cooperation Council (GCC) countries, focusing on ergonomic, physical, chemical, and biological hazards. It identifies significant impacts of these hazards across various professions and underscores the highlights for region-specific strategies and further research.

**Methods:**

A comprehensive search in Scopus, PubMed, and Web of Science databases until October 2023 targeted occupational health studies in the GCC, adhering to PRISMA guidelines and NIH Quality Assessment Tools. The protocol was registered on PROSPERO (CRD42023465909).

**Results:**

From 2202 articles screened, 202 were included, with publications distributed as follows: Saudi Arabia (121), United Arab Emirates (26), Kuwait (20), Oman (15), Qatar (13), and Bahrain (7). Findings indicate that ergonomic hazards, characterized by musculoskeletal disorders, are influenced by job roles and work environments. Physical hazards, particularly needlestick injuries, eye safety concerns, and risks from extreme temperatures and radiation, were notable across the region. Chemical hazards, including exposure to pesticides, cement, and petrochemicals, were identified as significant health risks, necessitating better safety measures. Biological hazards, evidenced in studies from Oman, UAE, and Saudi Arabia, highlighted the risks from infectious agents and parasites, stressing the need for effective prevention and hygiene practices.

**Conclusion:**

The review advocates for the urgent development of comprehensive health policies to mitigate occupational hazards in the GCC. It highlights the need for collaborative efforts to address ergonomic challenges, enhance protective measures, and respond to chemical and biological risks effectively. Region-specific strategies, ongoing research, and technological advancements are crucial for ensuring workforce safety in these evolving environments.

## 1. Introduction

Occupational Health and Safety (OSH), including various aspects like workplace health and safety, is a crucial field focusing on the holistic well-being of workers in diverse occupations [1]. The global workplace sees over 2.78 million fatalities and 374 million non-fatal injuries annually due to occupational accidents or diseases [2, 3]. The industrial revolution and technological advancements have increased unsafe working conditions across a broad spectrum of occupations [4]. Such advancements have introduced new challenges in OSH, from increased pesticide use to interactions with nanomaterials, and ergonomic issues [5–7].

Industrialization also brought significant improvements in OSH, primarily through the efforts of entities like the ILO [8]. However, recent socio-economic and political developments necessitate ongoing adaptation of labor laws to protect worker health and safety [9]. The GCC countries, experiencing rapid industrial and economic growth, have seen an influx of workers from various regions, bringing diverse OSH challenges to the forefront [4]. Studies indicate that contemporary OSH risks are multi-faceted, involving unexpected accidents, and a range of physical, chemical, and biological exposures [10–12].

Globally, the increasing trend in occupational diseases necessitates concerted efforts to enhance workforce health and safety [13]. Despite advances in injury prevention methods, specific occupations still report high rates of traumatic injuries [14]. The fourth industrial revolution introduces new challenges in OSH, including technological developments that could lead to musculoskeletal disorders [15,16]. Organizational changes and the demands for new learning contribute to increased psychosocial stress, emphasizing the need for innovative approaches to OSH management [15].

In this context, this systematic review aims to provide a comprehensive review of the challenges, trends, and developments in occupational health from a GCC perspective. While significant research and implementation efforts have been documented in regions like the USA, EU, and Asia, there is a notable gap in understanding the unique occupational health challenges in GCC countries. This review aims to bridge the existing gap, exploring significant occupational health risks and challenges specific to the GCC, with the goal of contributing to the effective implementation of occupational health measures, thereby ensuring the health and safety of the workforce in such rapidly developing countries.

## 2. Methods

The current systematic review was carried out in line with the guidelines of the PRISMA framework [17]. Our research involved a comprehensive English-language literature search in the Scopus, PubMed, and Web of Science databases up to October 2023. The decision to focus on English-language literature was made because English is the predominant language for scientific research both globally and within the GCC countries. Additionally, this approach ensures a broad and relevant spectrum of high-quality studies, facilitates consistent and standardized analysis, and addresses resource limitations that prevent accurate and comprehensive translation and interpretation of studies in other languages. We focused on studies pertinent to the six GCC countries, using ’Occupational health’ as our primary search term. Despite using just this single keyword, the results were already adequate, yielding a comprehensive overview of various hazards. We identified a significant number of potential hazards, aligning with our primary aim to aggregate and analyze studies in the GCC to assess occupational health in the area. These countries were included because they share a common regional organization (the GCC) that coordinates their economic, social, and political policies, including those related to occupational health. Additionally, their rapidly developing economies and significant industrial and construction sectors make occupational health a critical area of concern.

The inclusion criteria for the study were set to include publications in English that presented original research relevant to occupational health within the GCC. Eligible studies were primary studies with either descriptive or analytical observational designs. Only studies published in peer-reviewed journals were included, ensuring a level of quality control and scientific rigor. The studies needed to have a clearly defined aim, indicating a clear research question or objective. Geographic relevance was also a criterion, with studies pertaining to the GCC regions (Bahrain, Kuwait, Oman, Qatar, Saudi Arabia, and the UAE) being included. The exclusion criteria were established to omit gray literature, review articles, studies beyond the scope of the search terms, and publications specifically related to COVID-19 and psychosocial hazards. We categorized the selected studies based on their focus on physical, chemical, biological, or ergonomic factors. The review protocol was proactively registered on PROSPERO (CRD42023465909), an international database of systematic review protocols managed by the University of York’s Center for Research and Dissemination and funded by the National Institute for Health Research. It includes protocols for systematic reviews across various sectors, including public health, social care, and international development. By providing a comprehensive listing of these protocols, PROSPERO aims to minimize duplication of effort, reduce reporting bias, and enhance transparency, which helps prevent plagiarism and fosters a more open research environment [18].

Two independent reviewers systematically assessed study eligibility using predefined criteria. They initially screened abstracts with Covidence software and subsequently reviewed full articles to determine inclusion suitability. Data from selected studies were extracted independently by both researchers using a standardized template. S1 Table details findings from selected studies.

For evaluating the methodological soundness of included studies, we applied checklist criteria from the National Institutes of Health [https://www.nhlbi.nih.gov/health-topics/study-quality-assessment-tools]. Each study was appraised and classified as either poor, fair, or good based on responses to a sequence of questions. The grading system varied depending on the study type: observational cohorts and cross-sectional studies were rated on a scale from 0 to 14, case-control studies from 0 to 12, and case series studies from 0 to 9. Completed checklists for all included studies can be accessed in S2 and S3 Tables.

## 3. Results

**Fig 1:**
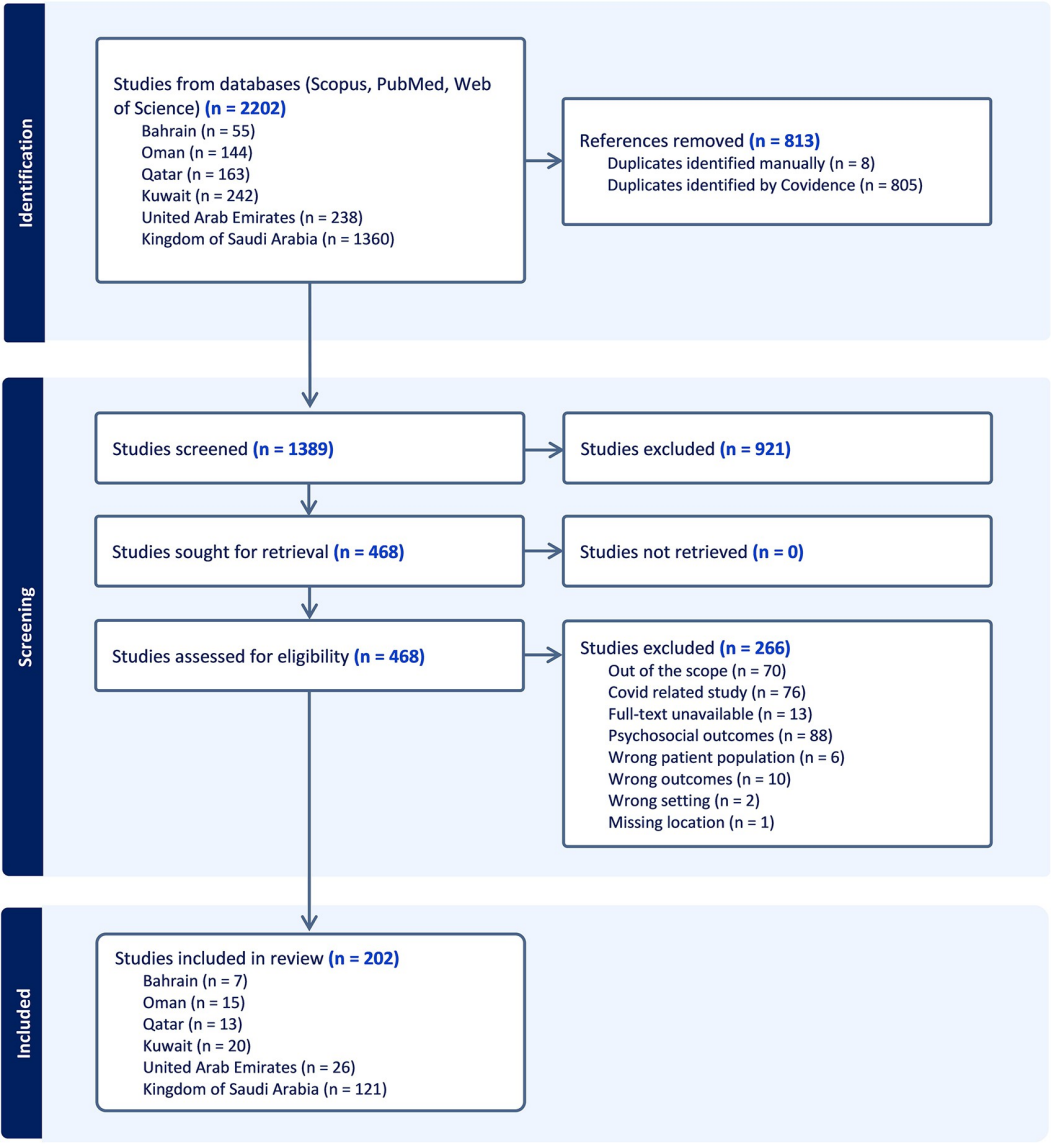
Screening process of the systematic review using the Preferred Reporting Items for Systematic Reviews and Meta-Analyses [PRISMA].

The systematic review process, Fig 1, involved an extensive literature search that identified 2202 records. Out of these, 202 studies met the eligibility criteria and were included in the review.

The bias analysis using NIH Quality Assessment Tools indicated moderate methodological quality in the reviewed occupational health studies. Studies were categorized as poor if they met less than 30% of the criteria, as fair if they fell within the 70% range, and as good if they exceeded 70%, according to the NIH Quality Assessment scoring system. In Bahrain, 85.7% of studies were rated as fair, and 14.3% as poor. Oman had 86.7% fair and 13.3% good. Qatar, Kuwait, and the UAE saw all studies rated as fair. Saudi Arabia had 92.6% fair, 4.1% poor, and 3.3% good ratings among its 121 studies. Overall, 97% of studies achieved good (3%) or fair (94%) quality, with only 3% rated poor (S3 Table). Most studies adequately described their research questions, methods, and study population selection. However, some common limitations were observed, including the absence of sample size justification, non-blinding of outcome assessors, and inadequate statistical analysis. This overview suggests that while occupational health research in the GCC predominantly achieves fair quality, there is room for improvement, particularly in the areas of study design and reporting standards.

**Fig 2:**
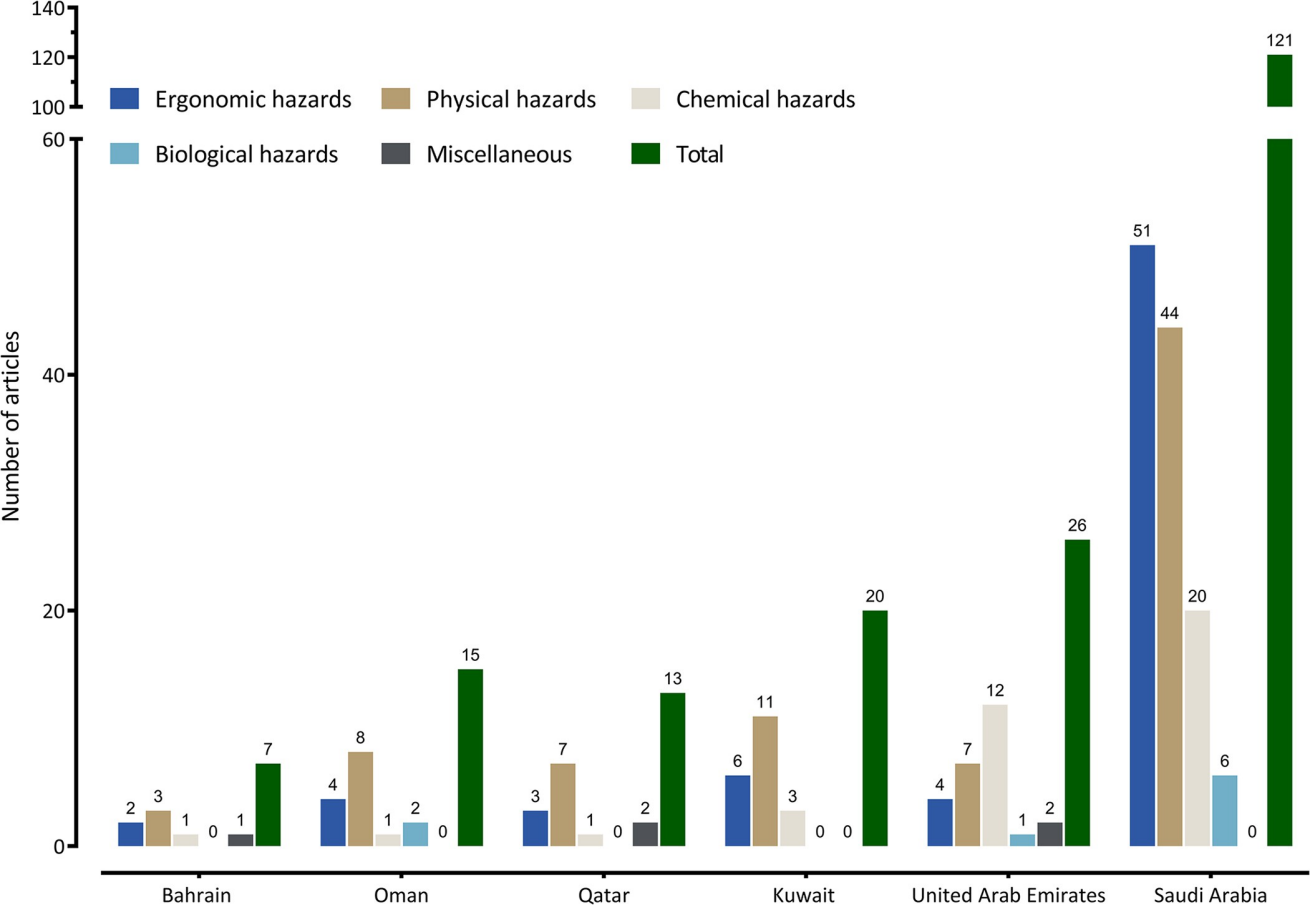
Summary of occupational hazard studies included in this review, as of October 2023.

The following section presents the results of the four major occupational hazards identified across the GCC region: ergonomic, physical, chemical, biological hazards, and miscellaneous (which represent diverse and uncategorized risks such as cardiovascular diseases, brain injuries, and unspecified occupational injuries) for each country within the GCC. Fig 2 provides a summary of the occupational hazard studies included in this review by country.

### 3.1. Kingdom of Bahrain

#### 3.1.1. Ergonomic hazards (n = 2)

A survey at Ahlia University with 200 participants highlighted that 44.5% suffered from low back pain and 40% from neck pain over a year, with no BMI correlation found [19]. In contrast, a study across three hospitals involving 550 mostly female nurses revealed an 88.1% prevalence of MSD symptoms, especially in the lower back (72.3%), shoulder (52.8%), and neck (49%), with age, medical conditions, practice area, and shift type being significant factors [20].

#### 3.1.2. Physical hazards (n = 3)

A survey on NSIs among healthcare professionals showed that 30 out of 91 participants reported NSIs, evenly split between doctors and nurses. Notably, just over half had received infection prevention training, with a notable number of nurses and doctors acknowledging the risks of needle recapping [21]. Research on eye safety in the workplace showed that 95.2% of eye injuries occurred in workers not wearing protective eyewear, primarily due to grinding activities. This underscores the high risk to construction workers and welders, highlighting the critical need for eye protection [22]. A study on the impact of climate-related conditions on expatriate laborers revealed that a significant portion of medical visits stemmed from CRC, mainly among those aged 26–35, with infectious diseases and heat-related illnesses being top concerns [23].

#### 3.1.3. Chemical hazards (n = 1)

Research on charcoal meat grilling workers analyzed Carboxyhemoglobin (%COHb) levels in 100 males, mostly aged 26–35. Initially, smokers showed 3.8% COHb and nonsmokers 2.4%. Post-shift, levels increased to 8.1% and 6.2%, surpassing the WHO and NIOSH 5% standard [24].

#### 3.1.4. Miscellaneous (n = 1)

This study on Bahraini government employees found that over 21% had high blood pressure, 24.2% had elevated cholesterol, and there were notable HDL level differences between males (64.1%) and females (26.6%). Despite 50.8% reporting no physical activity, 95.35% presented fewer than three cardiovascular disease risk factors [25].

### 3.2. Sultanate of Oman

#### 3.2.1. Ergonomic hazards (n = 4)

One study on oil rigs revealed ergonomic deficiencies causing worker discomfort, while another study identified workstation design flaws leading to pain [26, 27]. The negative effects of long hours on oil and gas workers in Oman were also reported. Furthermore, a study demonstrated the success of ergonomic interventions in an Omani refinery, effectively reducing health complaints [28, 29].

#### 3.2.2. Physical hazards (n = 8)

In a series of occupational health studies, high injury rates during initial jumps at the Sultan’s Oman Parachute Unit were reported [30]. The health effects of fasting during Ramadan in a high-temperature environment at an aluminum smelter were investigated, indicating the necessity for administrative adjustments [31]. A study documented prevalence of non-fatal injuries, such as eye injuries and falls, at the Harweel oil field, noting younger workers as especially vulnerable [32]. Three studies demonstrated that radiation doses to medical staff in Oman remained within safe limits, in alignment with international standards [33–35]. Additionally, a significant incidence of Noise-Induced Hearing Loss at Muscat International Airport called for enhanced noise control and protection measures [36]. Research at a construction project revealed the risks of vibration exposure, indicating the potential for Hand-Arm Vibration Syndrome [37].

#### 3.2.3. Chemical hazards (n = 1)

A study on 74 greenhouse workers revealed widespread misuse of pesticides and scant use of protective gear, leading to skin irritation and headaches among 70.3% and 33.8% of workers, respectively [38].

#### 3.2.4. Biological hazards (n = 2)

A study confirmed CCHF among animal-related workers, with a significantly higher prevalence in non-Omani citizens [30.3%] compared to Omanis (2.4%), emphasizing the risk to butchers [39]. Additionally, a case highlighted doxycycline’s limitations as post-exposure prophylaxis for brucellosis in a veterinary assistant after a NSI [40].

### 3.3. Qatar

#### 3.3.1. Ergonomic hazards (n = 3)

A study at a fertilizer plant involving 2,562 employees showed that shift workers (648 participants) had a 13.5% incidence of CHD, significantly higher than the 7.1% among daytime workers, indicating a link between shift work and cardiovascular disease [41]. A significant impact of lower back pain on nurses at Hamad General Hospital was reported, affecting their work and daily life [42]. Additionally, Hanna et al.’s study on Qatar University employees showed 61.2% suffered from back pain, with sedentary behavior increasing the risk. Depression was also linked to all categories of back pain [43].

#### 3.3.2. Physical hazards (n = 7)

A series of studies have examined various occupational physical hazards. A study revealed that 20.9% of healthcare workers at Hamad Medical Corporation suffered NSIs, notably affecting females and increasing virus transmission risks [44]. Bener et al. observed head and neck injuries from falls, predominantly among young males, with neck injuries being especially severe [45]. Traffic accidents frequently caused head injuries [46], and heat stress contributed to higher cardiac mortality among Nepali workers, highlighting the need for enhanced heat protection [47]. Additionally, an incidence of 0.8% NSIs among healthcare providers in operating theaters was reported without subsequent seroconversion [48]. Unreported sharp injuries were prevalent among dental professionals [49], and significant occupational fatalities and injuries underscored the critical need for improved safety [50].

#### 3.3.3. Chemical hazards (n = 1)

A study identified major gaps in pesticide safety among farmworkers: only 2% knew pesticide names, a third were unsure about application amounts, most lacked protective gear, and 18% were unaware of health risks. Detectable levels of DAP metabolites in all workers underscore the need for enhanced safety education and practices [51].

#### 3.3.4. Miscellaneous (n = 2)

A study examined CHD risks among 369 Qatar Petroleum employees, finding firefighters, despite higher tobacco use, had a lower CHD risk [6.5%] than non-firefighters (9.5%), with non-firefighters showing higher diabetes rates [52]. Another study at Hamad Trauma Center showed 30.7% of 6,555 trauma admissions (2010–2013) were work-related, translating to a non-fatal injury rate of 37.34 per 100,000-workers and a fatality rate of 1.58 per 100,000. Despite workforce growth, a decline in severe injuries was observed, highlighting the ongoing necessity for enhanced workplace safety [53].

### 3.4. Kuwait

#### 3.4.1. Ergonomic hazards (n = 6)

Numerous studies have underscored MSD among healthcare workers. About 70% of physical therapists faced significant work-related lower back pain [54]. Another investigation found 70.9% of orthopedic hospital staff suffered from lower back pain, mostly from moving patients [55]. Further, 47.6% of physical therapists reported MSDs, with little effect on job performance [56]. A survey highlighted carpal tunnel syndrome in 18.7% of office workers, notably in women and overweight individuals [57]. Around 48% of physical therapists experienced MSDs due to manual work and lifting patients [58]. Additionally, 47% of dentists faced similar issues, especially those with extensive practice years, impacting work attendance [59].

#### 3.4.2. Physical hazards (n = 11)

A study observed a decline in radium-226 among dial-painters but noted an uptick in byproducts [60]. Another found radiology workers’ annual radiation doses surpassing global norms [61]. A third study confirmed radiation exposure for nuclear medicine and diagnostic staff remained under ICRP guidelines [62]. Omar et al. discovered a 0.7% blood exposure rate in healthcare workers, mainly from NSIs [63]. Research showed nuclear medicine department staff’s eye radiation doses stayed within safety limits, though interventional cardiologists and nurses might face higher risks [64, 65].

Classroom noise sometimes breached recommended levels, impacting students and educators [66], while another study cited a 20.4% prevalence of noise-induced hearing loss among migrants [67]. Kuwait’s first asbestosis case in an asbestos-cement plant worker was documented [68]. Research on 22 workers highlighted dehydration-triggered hypokalemic paralysis [69]. Lastly, a study linked high temperatures to increased occupational injury risks despite work hour adjustments [70].

#### 3.4.3. Chemical hazards (n = 3)

Research on cement dermatitis in 191 male workers revealed 108 chronic cases, all sensitive to potassium dichromate. Symptoms peaked between April and November, linked to temperature and humidity enhancing chromate absorption [71]. Another study during Kuwait’s locust invasion assessed pesticide exposure in 74 workers, showing lung function variations and decreased plasma cholinesterase, hinting at effects from organophosphate and lindane [72]. An examination of 460 gas station workers, mostly South Asians, identified eye issues, respiratory problems, allergies, and headaches [73].

### 3.5. United Arab Emirates

#### 3.5.1. Ergonomic hazards (n = 5)

A survey of 202 dental students revealed 68.3% experienced MSDs, unaffected by gender. Contributing factors included trauma history, lack of exercise, extended clinical sessions, and elevated BMI [74]. Another study of 60 male dentists found 83% reported MSD, primarily in the lower back, with chronic pain and postural deviations in the neck and shoulders significantly associated with MSD [75]. Further investigation across various dental specialties highlighted high MSD rates, especially among periodontists, conservative dentists, and endodontists, with distinct pain distribution patterns based on specialty [76]. A broader survey involving 179 dentists showed a staggering 90.4% experienced MSDs, significantly influenced by workplace stress, gender, age, professional experience, and the availability of ergonomic equipment. The lack of ergonomic chairs and high workplace stress were identified as major contributors [77].

#### 3.5.2. Physical hazards (n = 7)

Research spanning 2002–2016 across nine hospitals assessed occupational radiation exposure in 1011 radiology and cardiology medical workers. Cardiologists recorded the highest mean annual effective dose of 0.78 mSv, with others ranging from 0.42 to 0.45 mSv, all within safety standards [78]. In Al-Ain City, 35% of 500 small-scale industrial workers reported eye injuries, attributing to less education, safety training, and protective gear use, with high-risk activities being arc welding, chipping, and drilling [79]. Among 2,573 trauma patients in a UAE study, 6% suffered from work-related injuries from falling objects, mostly affecting extremities, with a 1.3% mortality rate [80]. Furthermore, 468 steel workers were exposed to noise levels above 85 dB[A], with only 13.2% using hearing protection regularly, indicating low hazard awareness [81]. A survey among 177 dental professionals revealed 21% experienced hearing problems, primarily tinnitus, affecting clinic communication [82]. Another study found dental clinic noise levels averaged 70.60 ± 7.53 dBA, with those experiencing constant tinnitus being more affected [83]. Additionally, healthcare workers faced a high sharps injury risk, mitigated by adhering to standard precautions, with female gender notably less likely to report such injuries [84].

#### 3.5.3. Chemical hazards (n = 12)

In Al-Ain, industrial workers had notably higher blood lead levels, linked to various health issues [85], and showed adverse health markers from lead exposure [86]. A cement factory investigation found increased respiratory symptoms among exposed workers, emphasizing the protective role of masks [87]. Pesticide-exposed farm workers showed decreased acetylcholinesterase activity, stressing the need for proper handling training [88].

Older cement factory workers and prolonged-exposure welders in Ajman presented with chronic respiratory conditions and elevated metal levels, respectively [89, 90]. Industrial workers exposed to lead faced liver and kidney function risks [91]. Compared to non-farm workers, farm workers exposed to pesticides reported more health symptoms, underscoring pesticide exposure risks [92]. Cement workers exhibited lower ventilatory functions and more respiratory symptoms [93], while taxi and garage workers faced increased chronic respiratory issues, suggesting environmental and occupational exposures as contributing factors [94, 95]. Farmers dealing with pesticides showed severe dermo-respiratory symptoms, highlighting the critical health impacts of pesticide use [96].

#### 3.5.4. Biological hazards (n = 1)

A study in Sharjah on 21,347 expatriate workers, mostly food handlers and housemaids, found a 3.3% intestinal parasite infection rate [97]. Protozoa caused 67.4% of infections, helminths 29.1%, with Indian nationals most affected.

#### 3.5.5. Miscellaneous (n = 1)

A UAE survey among 844 dentists highlighted occupational health issues: 68% reported MSD, 42% had percutaneous injuries, 18% suffered contact dermatitis from latex gloves, 53% encountered eye problems, and 5% had hearing issues [98]. Additionally, a study on traumatic brain injuries (TBI) at an Abu Dhabi hospital between 2005 and 2009 analyzed 581 cases, identifying 10% as work-related, predominantly among male migrant workers, with falls and falling objects as primary causes [99].

### 3.6. Kingdom of Saudi Arabia

#### 3.6.1. Ergonomic hazards (n = 51)

Several studies across Saudi Arabia have underscored the ergonomic risks within various professions. Research has consistently revealed a high prevalence of neck and back pain among dentists, dental auxiliaries [100], and construction workers, with lower back pain being especially common [101]. Factors such as gender, occupation, nationality [102], extended working hours, and low body weight [103] have been identified as significant risk factors for MSDs among healthcare workers.

Further analysis highlighted similar concerns among construction workers and dentists in the Ha’il Region [104, 105], and eye care professionals and cabin crew members of Saudi Airlines, who commonly reported neck and back pain [106, 107]. Operating room healthcare staff also exhibited a significant association between lower back pain and risky activities [108].

Subsequent studies [109–113] emphasized the correlation between long working hours, specific work habits, and MSDs among physical therapists and dentists. The period between 2017 and 2023 saw a comprehensive examination of MSDs across various professions in Saudi Arabia, revealing a high incidence of such disorders among sonographers, dental practitioners [114, 115], and Arabic calligraphers [116].

Recent research from 2019 to 2023 further examined the prevalence of low back pain among paint factory workers, healthcare workers, and surgeons [117–119]. Studies pointed out the impact of occupational stressors on conditions such as carpal tunnel syndrome among dentists and MSDs among nurses [120, 121]. MSDs in female secondary school teachers and radiologists were also reported [122, 123].

The findings from these series of studies [124–150] reinforce the need for improved ergonomic interventions, and comprehensive strategies to mitigate MSD risks, to ensure occupational well-being in Saudi Arabia.

#### 3.6.2. Physical hazards (n = 44)

Forty-four articles shed light on physical hazards, grouped into Radiation, NSIs, and a collective category of Noise, Heat, and Falls.

Radiation exposure studies (n = 12) from 2015–2023 highlight significant findings across various sectors. Research at King Faisal Specialist Hospital and Research Centre showed cardiologists’ higher eye radiation doses than nurses and technologists [151]. A study pointed to inadequate protective gear in Eastern Province healthcare facilities [152], while another assessed radionuclide concentrations in Al-zabirah’s bauxite [153]. Reports confirmed that pilot’s exposure to ionizing radiation, as well as nuclear medicine staff exposures, remained within dose limits [154–156]. Additionally, a national retrospective on diagnostic radiology workers suggested enhanced radiation protection measures [157]. Assessments indicated dental workers’ exposure was safe [158], yet a study on thyroid radioiodine therapy found relatively high occupational exposures [159]. Research across Aseer’s radiology departments affirmed staff doses within acceptable limits [160], with significant findings also in ultrasound and PET/CT scanning exposures [161, 162].

NSIs [n = 15] revealed nurses as the most at-risk group, with 65% of incidents [163]. Studies identified risk practices in hospitals [164–171, 172–175], and a significant prevalence among dental assistants in Jeddah due to knowledge gaps [176]. Reports show higher risks in secondary hospitals [177] and a 19.7% prevalence among Jeddah nurses [174], emphasizing the need for targeted safety training and vaccinations [168].

Physical hazards studies (n = 17) underscore safety needs against noise, heat, and falls. Reports of ocular injuries in an iron forging industry [178], pulmonary declines in cement workers [179], and high incidence of non-fatal occupational injuries in Al-Khobar [180]. Hearing loss prevalence among dentists [181] and construction workers’ heat stress [182–184] highlight environmental and equipment-related risks, advocating for comprehensive health monitoring and protective strategies [185–191, 192–194].

#### 3.6.3. Chemical hazards (n = 20)

Ammonia exposure in urea fertilizer factories was correlated with increased respiratory symptoms [195, 196], and pesticide sprayers in Riyadh showed decreased acetylcholinesterase activity [197]. Firefighters in Jeddah and Yanbu experienced altered serum heavy metals and ferritin levels due to smoke exposure [198], while welders in Jeddah were found to be exposed to harmful fumes beyond safety limits [199].

Additionally, long-term exposure to cleaning chemicals among healthcare workers showed a potential impact on respiratory health, despite no significant symptoms [200]. Firefighters showed biochemical changes from smoke exposure [201], and nonsmoking workers in the plastic industry exhibited a high prevalence of diabetes, linked to work exposure duration [202]. Welders and gasoline pump attendants faced health issues from materials and benzene exposure [203, 204], with certain genetic variants increasing the risk of lead toxicity [205].

Respiratory symptoms were also reported among salon employees and cement industry workers [206, 207], as well as diabetes among workers in the cement, wood, welding, motor mechanics, and oil refinery industries [208, 209]. Small-scale industry workers showed increased respiratory risks and decreased lung function [210], with car garage workers reporting contact dermatitis [211]. Incense smoke inhalation was associated with lung impairments and diabetes among sellers [212, 213], and polyaromatic hydrocarbons raised oxidative stress and tumor marker levels [214].

#### 3.6.4. Biological hazards (n = 6)

Poultry slaughterhouse workers displayed an increased prevalence of warts, suggesting heightened infection risks [215]. Healthcare workers showed variable rates of *Staphylococcus aureus* nasal carriage [216]. A notable rise in sharps injuries within a Saudi hospital pointed to persistent risks of blood-borne pathogen exposure among medical staff [217]. Difficulties in managing tuberculosis exposure among healthcare personnel were reported in Riyadh, with particular emphasis on the need for improved testing and prophylaxis adherence [218]. A study identified an increased rate of respiratory pathogen acquisition among healthcare workers during Hajj, calling for better protective measures during large gatherings [219]. Additionally, a low incidence rate of certain zoonotic infections among abattoir workers in 2013 was observed, highlighting occupational risks from zoonotic pathogens [220].

## 4. Discussion

Overall, the analysis of 202 articles exploring occupational hazards in the GCC region highlighted a predominant focus on physical and ergonomic risks (Fig 2). Studies from Bahrain and Oman mainly addressed these hazards, with Qatar incorporating miscellaneous risks such as cardiovascular diseases with other unspecified occupational injury, and Kuwait and the UAE reporting primarily on physical and ergonomic hazards, the latter also noting chemical risks. Saudi Arabia, contributing the most studies, concentrated on ergonomic hazards. Biological hazards were minimally reported across the GCC, indicating a need for broader research in this area to fully understand occupational health challenges in the GCC.

Ergonomic hazards in the GCC exhibited distinct regional patterns. Bahrain’s studies on MSDs among university staff and nurses indicated occupational roles’ significant impact on MSD prevalence. Oman’s research covered ergonomic challenges in oil rigs to noise-induced hearing loss and vibration exposure risks, underlining the need for customized ergonomic solutions. In Qatar, the association between shift work and CHD raised concerns about the cardiovascular risks of irregular work schedules. Kuwait focused on healthcare professionals, revealing prevalent low back pain and MSDs, mainly from patient handling. UAE’s research showed widespread musculoskeletal symptoms among dental professionals. Saudi Arabia’s findings on MSDs across professions pointed to issues related to long work hours and physical strain, calling for comprehensive occupational health strategies.

Studies across the GCC underscored a range of occupational physical health hazards. Bahrain’s research on NSIs among healthcare professionals highlighted training gaps in infection prevention. In Oman, studies presented the physiological impacts of fasting in high temperatures and a high rate of non-fatal injuries in the oil sector. Qatar’s findings on the significant prevalence of NSIs and the link between heat stress and cardiac mortality among migrant workers were notable. Kuwait explored varied occupational hazards, including radiation exposure among radiology and nuclear medicine staff, noise-induced hearing loss, asbestos exposure, and heat-related injuries, prompting calls for improved safety measures across industries. The UAE’s research into occupational radiation and eye injuries, along with studies on injuries from falling objects and noise exposure, stressed the need for enhanced safety practices. Saudi Arabia offered an extensive overview of physical hazards, emphasizing the need for tailored safety strategies across sectors.

Occupational chemical hazards across various sectors were identified as significant health risks. In Bahrain, elevated Carboxyhemoglobin levels in meat grilling workers required better safety practices. Oman’s greenhouse workers faced health issues from improper pesticide handling, stressing the need for enhanced safety training. Similar challenges in Qatar with pesticide use among farmworkers emphasized the importance of improved handling practices. Kuwait’s cement dermatitis and petrochemical exposure in gas station workers suggested a need for increased protective measures. The UAE’s elevated blood lead levels and respiratory issues among industrial and cement factory workers highlighted the urgency for better protective gear and health monitoring. Saudi Arabian research across multiple sectors showed varied health impacts due to chemical exposures, emphasizing the need for comprehensive safety protocols and effective protective measures.

Occupational biological hazards presented significant health risks across various sectors. In Oman, the prevalence of CCHF among animal-related workers highlights the need for increased preventive measures in occupations involving animal contact. The importance of effective postexposure treatments was underscored by cases like brucellosis from NSIs in veterinary settings. A study in Sharjah, UAE, found a considerable incidence of intestinal parasite infections among expatriate workers. Saudi Arabia faced diverse biological hazards in its occupational environments, indicating significant risks from infectious agents and stressing the need for enhanced protective and compliance strategies.

**Fig 3:**
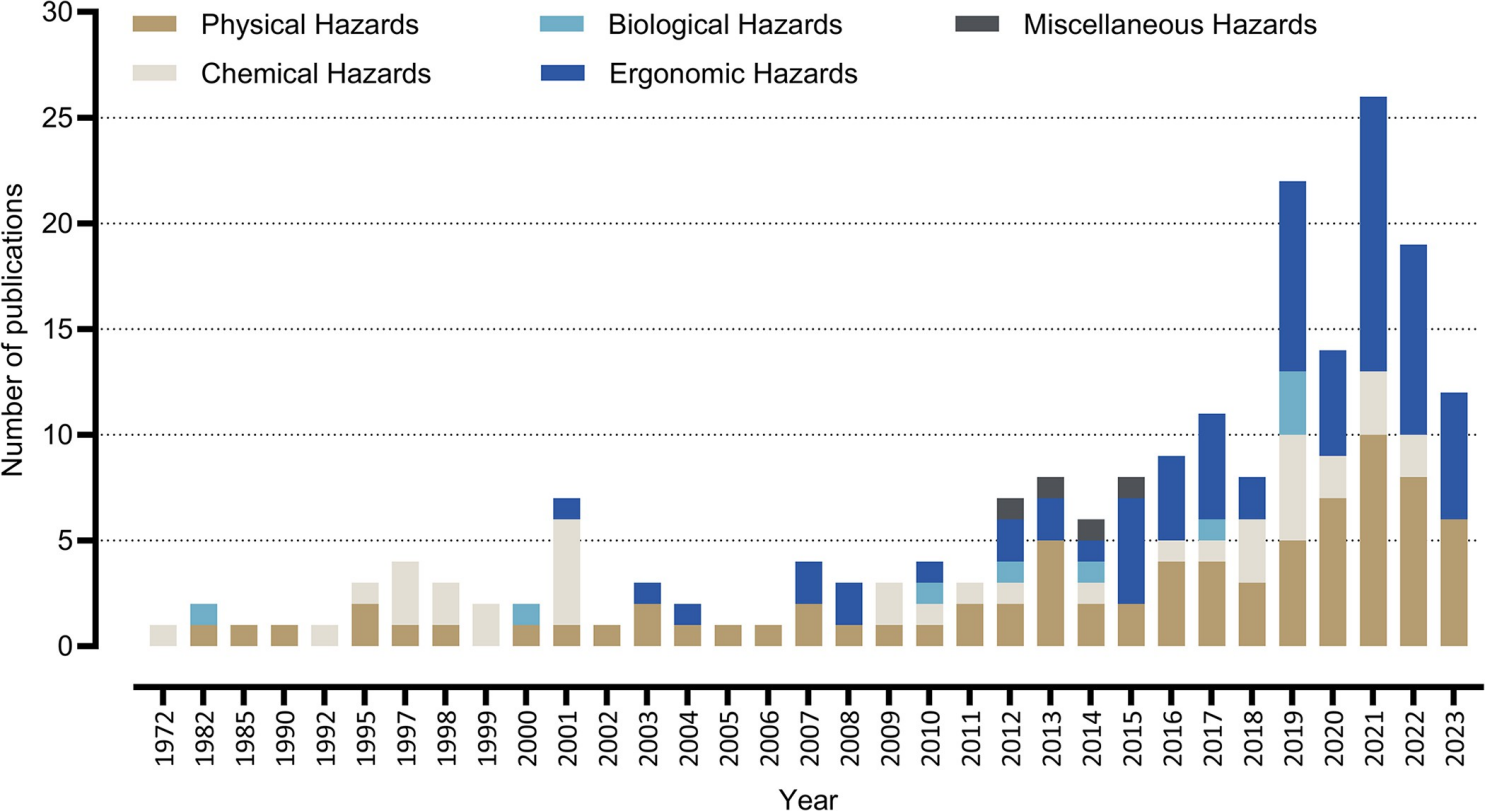
Annual distribution of articles included in the study, categorized by types of occupational hazards from 1972 to 2023 in the GCC.

Fig 3 illustrates the annual distribution of articles categorized by different occupational hazard types. There has been a steady rise in the number of studies starting from 2007, with a significant increase from 2019 onwards. Physical and ergonomic hazards have been extensively researched over the last seven years, while chemical hazards were more prominent in earlier studies, particularly during the 1990s. Biological hazards have gained more attention in recent years, though they still represent a smaller portion of the overall research.

A clear upward trend in occupational hazard research is visible, especially after 2015. The marked increase in ergonomic hazards may reflect a growing concern over workplace conditions that contribute to musculoskeletal disorders. The rise in studies on physical hazards starting from 2013–2015 underscores ongoing concerns about workplace safety, particularly regarding noise, heat, and vibration exposure. Initially, chemical hazards were a focal point, likely due to concerns over exposure to harmful substances in industrial settings. However, recent research has shifted to encompass a broader range of workplace risks. The relatively smaller focus on biological hazards suggests that these risks are less frequently studied, though they remain critical in specific sectors such as healthcare.

Overall, this analysis highlights the increasing recognition of physical and ergonomic risks in the workplace. The shift in research focus indicates growing awareness of non-chemical hazards and the need for specialized prevention strategies to address diverse occupational risks. The sharp rise in articles in recent years also reflects the continued importance of occupational health and safety across multiple sectors.

**Fig 4:**
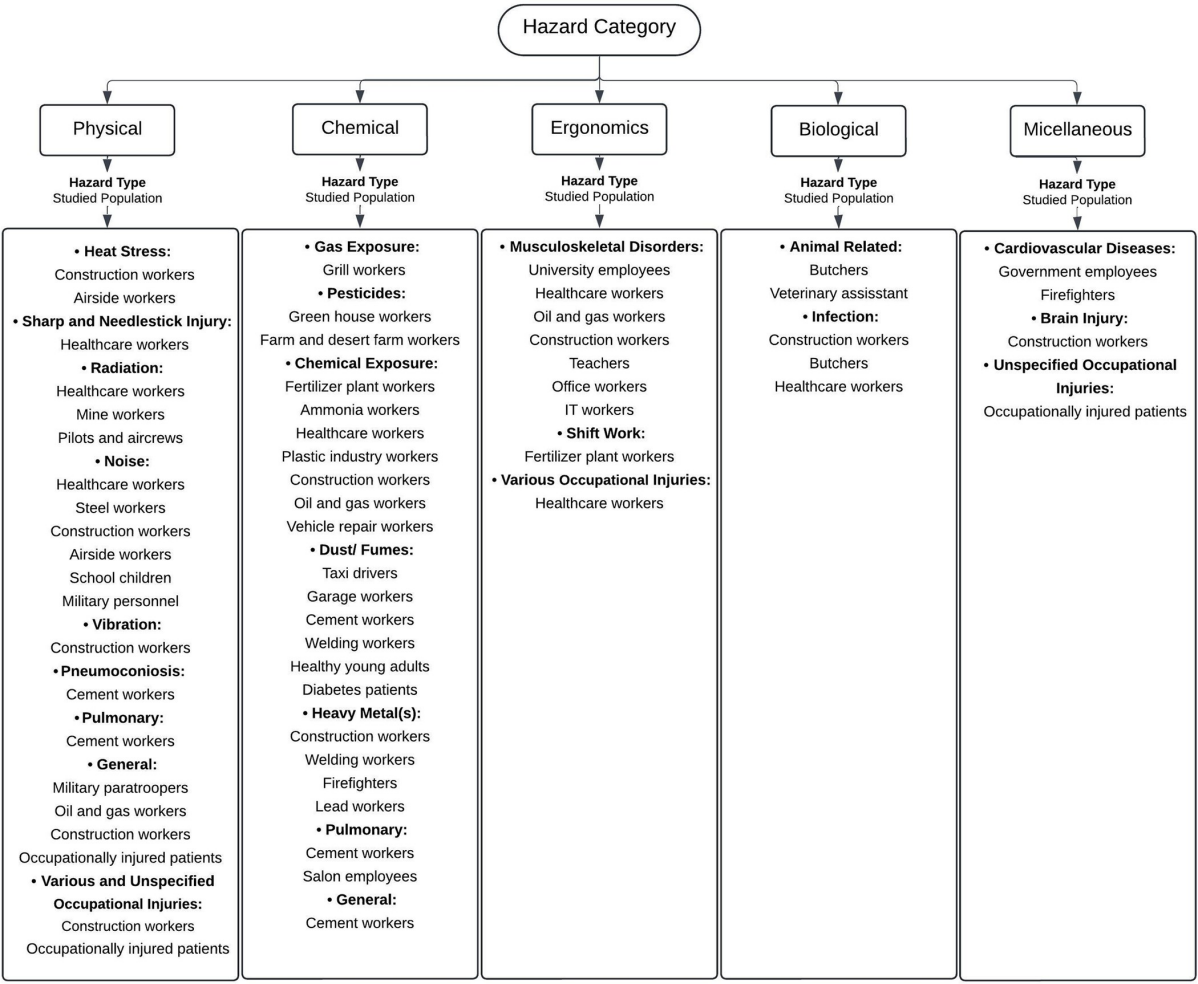
Overview of occupational hazard categories and affected occupations.

An overview of occupational hazard categories and affected occupations is illustrated in Fig 4. The flowchart outlines various hazard categories and the occupational groups affected. Chemical hazards, such as pesticides, gases, dust, and heavy metals, impact workers in industries like construction, manufacturing, and farming emphasizing the need for stringent safety measures. Physical hazards, including noise, radiation, and heat stress, are common in construction, healthcare, and military sectors, highlighting the importance of protective strategies. Ergonomic hazards affect a wide range of workers in healthcare, office, and teaching sectors. These hazards are linked to musculoskeletal disorders caused by long working hours, prolonged sitting or standing in awkward postures without breaks, and the extensive physical activity required to meet job demands. Biological hazards, including infections and animal-related injuries, primarily affect butchers and healthcare workers, stressing the importance of infection control. Miscellaneous hazards, such as cardiovascular risks, are observed in high-stress jobs like those of government staff, managers, and construction workers. The diagram illustrates that certain occupational groups face multiple hazards, with specialized safety protocols necessary to address specific risks in each sector.

**Fig 5:**
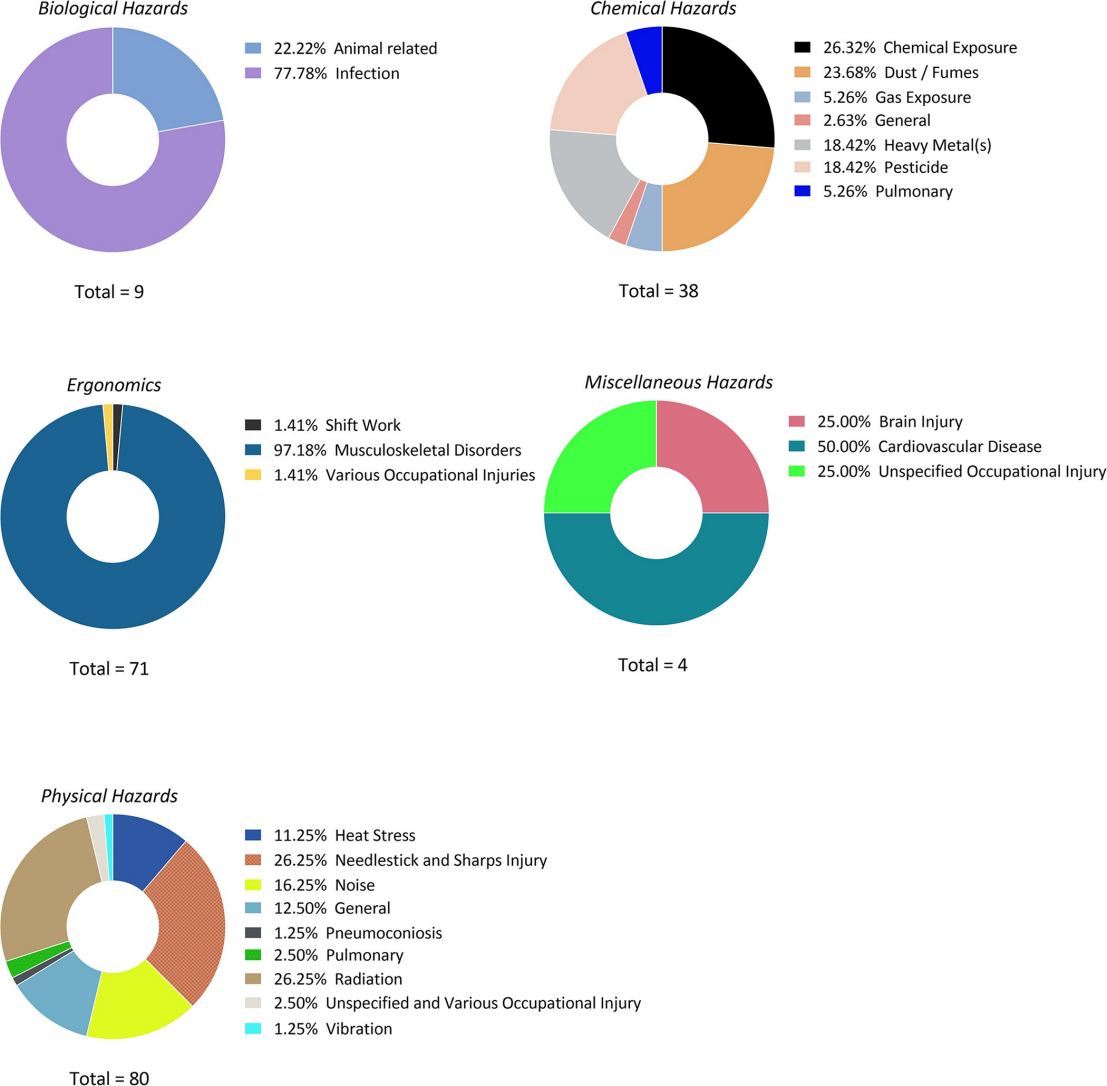
Distribution of occupational hazards across different categories.

Fig 5 illustrates the distribution of occupational hazards across four major categories: Physical, Chemical, Biological, and Ergonomic, with each pie chart showing the specific hazards and their relative prevalence within each category. Musculoskeletal disorders (97.18%) and infections (77.78%) represent the most significant ergonomic and biological hazards, respectively. This highlights the pressing need for targeted interventions in manual labor environments and healthcare settings, where workers are more vulnerable to these types of hazards. The widespread prevalence of these issues underscores the importance of focusing on preventative measures, such as ergonomic improvements and infection control protocols, to reduce the burden of workplace injuries and illnesses.

Additionally, the figure showed a broad range of occupational risks, varying significantly across different categories. In the chemical hazards category, chemical exposure (26.32%) and dust/fumes (23.68%) are the most prevalent risks, followed by heavy metal and pesticide exposure (both at 18.42%). In physical hazards, both radiation and needlestick and sharp injuries (26.25%) are the most prominent, while noise (16.25%) and general injuries (12.5%) also represent significant threats. Each of these hazards demands tailored safety interventions to protect workers in different industries and environments. One noteworthy observation is the low prevalence of shift work as an ergonomic risk, contributing only 1.41%. While musculoskeletal disorders dominate this category, the minimal impact of shift work suggests that most ergonomic injuries are linked to physical strain rather than irregular work schedules. This finding points to the importance of focusing on reducing physical stressors in the workplace, particularly for jobs that involve repetitive motions or heavy lifting.

Long-term health risks associated with hazards like chemical exposure, radiation, and heavy metal toxicity are another critical concern. These hazards often lead to chronic conditions that may not manifest immediately, illustrating the need for sustained protective measures in workplaces where such risks are prevalent.

The US NIOSH identifies the construction industry as having the highest heat stress-related fatalities, averaging 0.22 per million workers annually from 2000 to 2010 [221,222]. Studies from Japan [223], the US [224], and India [225] highlight kidney risks for construction workers, underscoring the importance of hydration. Findings from the GCC region support this, as studies involving migrant workers have reported that construction workers experience high heat stress, which may lead to cardiac mortality. NSSIs are frequent in healthcare, with over 1 million incidents annually, representing 8% of hospital injuries [226]. In sub-Saharan Africa, the lifetime prevalence of NSSIs ranges from 22% to 95% [227], yet these are preventable with proper safety protocols [228,229]. Globally, Occupational noise is linked to conditions like hypertension and type 2 diabetes [230–233], with dentists and construction workers particularly at risk for hearing loss [234–239]. Up to 50% of construction workers could develop HAVS due to high-vibration tools [240]. Radiation exposure in radiology and nuclear medicine affects up to 50% of workers, with many exceeding safety limits [241]. About 40% of uranium mine workers face increased lung cancer risks from radon [242]. Aircrew studies indicate elevated cancer risks among female cabin attendants [243,244]. Pneumoconiosis impacts around 9.3% of dust-exposed workers, influenced by age and smoking [245].

Chemical hazards significantly impact occupational exposure. A 2015 report by the European Agency for Safety and Health at Work highlighted that 17% of workers in the EU are exposed to hazardous chemicals for over a quarter of their working hours, particularly in industrial, agricultural, and laboratory settings [246]. Exposure to dusts and fumes, classified as a Group 1 carcinogen by the IARC, is linked to severe health outcomes like cardiovascular and respiratory diseases [247]. Additionally, pesticide exposure is a critical issue, especially in agriculture. The WHO reported approximately 3 million cases of pesticide poisoning annually, resulting in 220,000 deaths, mostly in developing regions where safety regulations are often inadequate [248–250]. About 44% of the global farming population suffers from pesticide poisoning each year [251]. Heavy metal exposure, notably to lead, poses significant risks, affecting over 1 million workers worldwide in industries like battery manufacturing and mining [252].

Ergonomic hazards, notably MSDs, pose significant risks globally. Over 50% of healthcare workers, particularly nurses, report work-related MSDs due to their physically demanding roles [253]. Dentists are similarly affected, with annual MSD prevalence in any body part reported between 68% to 100%, influenced by factors like gender, working postures, experience, and specialty [254]. Office workers, susceptible to MSDs from prolonged sitting and repetitive motions, show prevalence rates from 33.8% to 95.3%, with risks heightened by older age, gender, low education, and lack of exercise [255]. Construction workers face ergonomic challenges from heavy lifting, vibrating tools, and awkward positions, with MSD rates varying from 33% to 89% [256].

Miscellaneous hazards also impact health, with research affirming the link between occupation and cardiovascular health [257]. Approximately 80% of cardiovascular deaths occur in low- and middle-income countries, underscoring socioeconomic status as a significant factor influencing cardiovascular disease (CVD) prevalence [258].

Health and safety regulations have a long-established history in Great Britain, with origins tracing back to the 19th century. The current regulatory framework is built upon the Health and Safety at Work Act of 1974, which has contributed to the UK’s globally recognized safety record [259]. In the United States, the Occupational Safety and Health Act (OSH Act) was enacted in 1970, including a comprehensive set of requirements to protect workplace safety [260]. In the UAE, the Abu Dhabi Occupational Safety and Health (ADOSH) system, initiated in 2006 following guidelines from the American Conference of Governmental Industrial Hygienists (ACGIH), launched its first system framework in 2008. In July 2024, the system was updated with Version 4.0, which introduced new codes of practice for hazard and risk assessment [261]. Similarly, Qatar implemented its foundational legislation for occupational safety with Labor Laws No. 14 and No. 15 in 2004 [262]. Oman’s leading occupational safety regulation, the Labor HSE Code, was enacted in 2008 under Royal Decree No. 35/200. This regulation, along with additional ministerial decisions, governs critical areas such as chemical hazards, hazardous substances, noise exposure, and the establishment of OSH committees [263].

Although the approach to occupational health and safety across GCC countries emphasizes self-regulation, the methodologies for assessing workplace hazards vary significantly. This is in contrast to the United Kingdom and the United States, where enforcement agencies play a central role in ensuring compliance with safety regulations. Despite these differences in regulatory frameworks, the overarching objective across all regions remains consistent: to ensure the protection of workers’ health and safety in the workplace [259–263].

Occupational safety and health regulations vary across countries. For fall protection, for instance, the USA enforces 25 articles, while the UK has 19 articles and multiple schedules. Australia follows a detailed code of practice for working at height, and South Africa introduced "Work at Height" regulations in 2013. In contrast, Oman, as one of the GCC countries, despite falls being a major cause of accidents, only has a general occupational health and safety regulation from 2008, lacking comprehensive fall protection measures.

Likewise, occupational health and safety performance in construction sector is notably low in GCC due to factors such as limited safety awareness, weak regulations, and harsh environmental conditions. The hot and humid climate also impacts worker performance. Improving the safety climate requires strong management commitment, integration of safety as a core value, leadership by supervisors, worker involvement, better communication, and comprehensive training [264–266].

Chemical hazard communication regulations also differ. The USA’s 2012 regulations include 10 articles, while Australia’s Work Health and Safety Act contains 34 pages of guidelines. The UK, under EU law, adheres to an extensive regulation with 62 articles. South Africa has various Acts addressing chemical hazards, whereas Oman, as a GCC country, has less robust regulations, with only 12 sub-articles in a single section providing limited guidance [267]. This systematic review’s limitations include the exclusion of non-English studies, potentially introducing language bias, and the omission of gray literature. The focus was primarily on peer-reviewed articles. Future studies can further explore the results of this study by utilizing gray literature, i.e., governmental reports on occupational injuries. These reports or statistics could possibly provide additional insights about how different populations are affected by occupational hazards in each of the six GCC countries. Due to the limited availability, it can be included only if accessible. The review suggests future directions should target the development and implementation of interventions tailored to regional needs, enhance safety protocols, and deepen research into specific challenges within the GCC.

Investigations should probe the ergonomic challenges distinct to each GCC country, considering cultural and environmental impacts on workplace ergonomics. A study conducted in the GCC region exploring the impact of culture on application of ergonomics at workplace by employees showed that factors such as attitude, perceived behavioral control along with social influence had significant effects on employees’ intention to implement ergonomics at their workplaces. Although the cultures in GCC countries have minimal variances, a considerable percentage of workers in these countries are expatriates which increases the impact of culture on application of ergonomics at workplaces [268]. There’s an opportunity to assess the efficacy of ergonomic interventions and training programs suited to various professions, including healthcare, construction, and office work. Longitudinal studies are needed to evaluate the sustained benefits of such improvements on health and productivity.

Emphasis should also be placed on comprehensive safety training, especially for healthcare to mitigate NSIs, and in industrial sectors to bolster eye safety and mitigate risks from environmental conditions. Exploring innovative protective technologies for high-risk settings and studying the long-term effects of physical hazards on health could inform policy decisions.

Research into the long-term impacts of chemical exposures, particularly in petrochemical, agricultural, and manufacturing industries, is crucial. Developing safer chemical alternatives and promoting handling best practices would benefit worker safety.

Efforts to tackle biological hazards should include studies on effective prevention measures, the efficacy of vaccination programs, hygiene practices, and post-exposure treatments to lower infection rates.

A multi-disciplinary approach, involving collaboration across the GCC, is vital for addressing occupational health hazards effectively. Shared learning and region-specific guidelines could enhance OSH. Continued investment in research, education, and technology is essential for improving OSH in the GCC.

## 5. Conclusion

The systematic review of occupational health hazards across Gulf Cooperation Council countries emphasizes a prevalent focus on physical and ergonomic risks, revealing a significant gap in the study of chemical and biological hazards. To enhance occupational health and safety, it is essential to develop and implement customized ergonomic solutions tailored to the specific occupational roles and environments identified in each GCC country. For example, interventions in Bahrain should concentrate on ergonomic improvements for university staff and nurses, while Oman could benefit from extended efforts in oil and gas sectors to mitigate noise-induced hearing loss and vibration exposure risks.

Safety training programs must be enhanced to address gaps in infection prevention, particularly among healthcare professionals in Bahrain where needlestick injuries are prevalent. Training should also cover proper pesticide handling in Oman and Qatar, and safe practices for handling hazardous chemicals in Kuwait and the UAE. Increasing the availability and use of protective gear, particularly for workers exposed to chemical hazards, and implementing regular health monitoring for workers exposed to high levels of hazardous substances are crucial to prevent long-term health issues.

Research should expand to include biological hazards which are currently underreported, investigating the effectiveness of existing vaccination programs, hygiene practices, and post-exposure treatments in mitigating risks associated with biological exposures, especially in high-risk settings. Moreover, comprehensive health policies incorporating recent study findings should be developed to address the specific needs and challenges of each GCC country, focusing on reducing occupational injuries and diseases through improved workplace safety standards and practices.

Longitudinal studies are needed to assess the long-term benefits of occupational health interventions on worker health and productivity. Collaboration across disciplines to integrate insights from ergonomics, industrial hygiene, public health, and occupational psychology can create a holistic approach to occupational health. Leveraging technological advancements to develop innovative protective equipment and safety monitoring tools, and investing in research to explore the potential of new technologies in reducing occupational health risks, particularly in high-risk industries such as construction and manufacturing, are also recommended.

Encouraging collaboration among GCC countries to share best practices, research findings, and technological solutions tailored to the region’s specific occupational health challenges should aim to standardize safety protocols and improve regulatory frameworks across the region. Initiating educational campaigns targeting both workers and employers to raise awareness about the importance of occupational health and safety, focusing on the dangers of inadequate protective measures and the benefits of adhering to recommended safety practices, will further contribute to enhancing the safety and well-being of the workforce, thereby supporting the overall productivity and economic growth of the region.

## Supporting information

S1 Checklist (DOCX)

S1 Table A summary of included studies [Country, hazard categories, hazard types, sample size, study design, main findings, and references]. The current systematic review follows PRISMA guidelines and focuses on occupational health research in the six GCC countries. A comprehensive English-language search was conducted in Scopus, PubMed, and Web of Science, yielding relevant studies published up to October 2023. Only primary research published in peer-reviewed journals was included, with a focus on physical, chemical, biological, and ergonomic factors in occupational health. Studies specifically related to COVID-19, psychosocial hazards, and gray literature were excluded. The review protocol was registered with PROSPERO, and the inclusion process involved two independent reviewers (K.A. and M.M.) between November 2023 and January 2024. S1 Table provides a detailed summary of the findings from the selected studies.
(DOCX)

S2 Table NIH quality assessment of Included observational cohort, cross-sectional, case control case series studies. The methodological soundness of the included studies was evaluated using the National Institutes of Health (NIH) checklist criteria. Studies were rated as poor, fair, or good based on specific questions tailored to study type. Observational cohorts and cross-sectional studies were assessed on a 0–14 scale, case-control studies on a 0–12 scale, and case series on a 0–9 scale. Detailed checklist results for all studies are available in S2 Table.
(DOCX)

S3 Table Applying the NIH quality assessment tool for all the included studies. (DOCX)

S4 Table A Summary of excluded studies and reasons for exclusion. (DOCX)
