# Supplementary material for: Occupational health in the Gulf Cooperation Council (GCC): A systematic review and call for comprehensive policy development
Source: PLoS One. 2024 Dec 10;19(12):e0312251. doi: 10.1371/journal.pone.0312251 (PMC11630603; doi:10.1371/journal.pone.0312251)
Supplement: S3 Table — (DOCX) [file pone.0312251.s004.docx]

Table S3: Applying the NIH quality assessment tool for all the included studies.

| **Study** | **Total score** | **Quality rating (good, fair, or poor)** | **Study** | **Total score** | **Quality rating (good, fair, or poor)** |
| --- | --- | --- | --- | --- | --- |
| **Observational Cohort and Cross-Sectional Studies (n=187)** | | | | | |
| [25] | 8 | FAIR | [219] | 7 | FAIR |
| [24] | 4 | POOR | [186] | 8 | FAIR |
| [26] | 9 | FAIR | [105] | 6 | FAIR |
| [22] | 5 | FAIR | [106] | 7 | FAIR |
| [23] | 7 | FAIR | [152] | 8 | FAIR |
| [21] | 6 | FAIR | [107] | 6 | FAIR |
| [20] | 8 | FAIR | [108] | 8 | FAIR |
| [40] | 7 | FAIR | [188] | 9 | FAIR |
| [27] | 5 | FAIR | [111] | 6 | FAIR |
| [31] | 7 | FAIR | [201] | 12 | GOOD |
| [28] | 6 | FAIR | [109] | 5 | FAIR |
| [30] | 12 | GOOD | [110] | 5 | FAIR |
| [33] | 8 | FAIR | [153] | 10 | FAIR |
| [39] | 7 | FAIR | [172] | 8 | FAIR |
| [34] | 9 | FAIR | [115] | 5 | FAIR |
| [32] | 7 | FAIR | [189] | 6 | FAIR |
| [35] | 9 | FAIR | [112] | 5 | FAIR |
| [37] | 10 | FAIR | [116] | 5 | FAIR |
| [36] | 11 | GOOD | [114] | 5 | FAIR |
| [29] | 5 | FAIR | [202] | 8 | FAIR |
| [45] | 8 | FAIR | [113] | 5 | FAIR |
| [42] | 7 | FAIR | [154] | 7 | FAIR |
| [46] | 8 | FAIR | [155] | 8 | FAIR |
| [47] | 8 | FAIR | [117] | 5 | FAIR |
| [53] | 9 | FAIR | [171] | 5 | FAIR |
| [52] | 8 | FAIR | [203] | 8 | FAIR |
| [43] | 8 | FAIR | [204] | 7 | FAIR |
| [54] | 8 | FAIR | [123] | 6 | FAIR |
| [44] | 8 | FAIR | [208] | 7 | FAIR |
| [48] | 8 | FAIR | [173] | 5 | FAIR |
| [49] | 7 | FAIR | [121] | 7 | FAIR |
| [51] | 6 | FAIR | [207] | 8 | FAIR |
| [50] | 5 | FAIR | [221] | 9 | FAIR |
| [62] | 7 | FAIR | [119] | 6 | FAIR |
| [55] | 7 | FAIR | [120] | 5 | FAIR |
| [56] | 7 | FAIR | [190] | 8 | FAIR |
| [57] | 6 | FAIR | [118] | 6 | FAIR |
| [58] | 6 | FAIR | [205] | 8 | FAIR |
| [63] | 9 | FAIR | [122] | 4 | POOR |
| [64] | 6 | FAIR | [209] | 8 | FAIR |
| [67] | 10 | FAIR | [206] | 7 | FAIR |
| [65] | 7 | FAIR | [124] | 6 | FAIR |
| [74] | 6 | FAIR | [156] | 11 | GOOD |
| [59] | 6 | FAIR | [220] | 10 | FAIR |
| [68] | 6 | FAIR | [125] | 5 | FAIR |
| [66] | 7 | FAIR | [211] | 9 | FAIR |
| [60] | 6 | FAIR | [129] | 6 | FAIR |
| [71] | 6 | FAIR | [157] | 8 | FAIR |
| [90] | 6 | FAIR | [126] | 5 | FAIR |
| [96] | 8 | FAIR | [194] | 8 | FAIR |
| [86] | 8 | FAIR | [127] | 5 | FAIR |
| [87] | 8 | FAIR | [130] | 7 | FAIR |
| [94] | 7 | FAIR | [210] | 8 | FAIR |
| [85] | 6 | FAIR | [195] | 7 | FAIR |
| [82] | 8 | FAIR | [128] | 5 | FAIR |
| [88] | 8 | FAIR | [191] | 11 | GOOD |
| [99] | 6 | FAIR | [192] | 11 | GOOD |
| [83] | 8 | FAIR | [159] | 8 | FAIR |
| [81] | 8 | FAIR | [135] | 7 | FAIR |
| [77] | 6 | FAIR | [134] | 8 | FAIR |
| [98] | 8 | FAIR | [133] | 7 | FAIR |
| [76] | 6 | FAIR | [136] | 7 | FAIR |
| [79] | 9 | FAIR | [138] | 8 | FAIR |
| [80] | 7 | FAIR | [143] | 7 | FAIR |
| [75] | 5 | FAIR | [160] | 8 | FAIR |
| [78] | 7 | FAIR | [212] | 8 | FAIR |
| [84] | 9 | FAIR | [131] | 6 | FAIR |
| [216] | 4 | POOR | [175] | 6 | FAIR |
| [164] | 5 | FAIR | [137] | 6 | FAIR |
| [179] | 6 | FAIR | [213] | 8 | FAIR |
| [180] | 10 | FAIR | [174] | 6 | FAIR |
| [196] | 8 | FAIR | [132] | 7 | FAIR |

| [181] | 10 | FAIR | [142] | 4 | POOR |
| --- | --- | --- | --- | --- | --- |
| [182] | 8 | FAIR | [158] | 9 | FAIR |
| [197] | 8 | FAIR | [176] | 6 | FAIR |
| [101] | 5 | FAIR | [215] | 9 | FAIR |
| [165] | 6 | FAIR | [146] | 4 | POOR |
| [166] | 6 | FAIR | [177] | 6 | FAIR |
| [183] | 8 | FAIR | [140] | 7 | FAIR |
| [187] | 9 | FAIR | [139] | 7 | FAIR |
| [167] | 6 | FAIR | [145] | 7 | FAIR |
| [184] | 7 | FAIR | [141] | 7 | FAIR |
| [169] | 6 | FAIR | [147] | 7 | FAIR |
| [198] | 9 | FAIR | [161] | 7 | FAIR |
| [199] | 8 | FAIR | [144] | 6 | FAIR |
| [168] | 5 | FAIR | [214] | 8 | FAIR |
| [217] | 7 | FAIR | [149] | 5 | FAIR |
| [200] | 7 | FAIR | [150] | 7 | FAIR |
| [218] | 6 | FAIR | [151] | 8 | FAIR |
| [103] | 4 | POOR | [178] | 5 | FAIR |
| [170] | 6 | FAIR | [162] | 7 | FAIR |
| [102] | 5 | FAIR | [163] | 8 | FAIR |
| [185] | 9 | FAIR | [148] | 6 | FAIR |
| [104] | 7 | FAIR |  |  |  |
| **Case-Control Studies (n=8)** |  |  |  |  |  |
| [73] | 7 | FAIR | [93] | 8 | FAIR |
| [95] | 7 | FAIR | [92] | 8 | FAIR |
| [89] | 8 | FAIR | [91] | 7 | FAIR |
| [97] | 8 | FAIR | [193] | 7 | FAIR |
| **Case Series Studies (7)** |  |  |  |  |  |
| [38] | 6 | FAIR | [69] | 6 | FAIR |
| [41] | 4 | FAIR | [70] | 9 | GOOD |
| [72] | 8 | GOOD | [100] | 8 | GOOD |
| [61] | 6 | FAIR |  |  |  |

**References:**

1. Tantawy SA, Halwachi B, Ghanim A, Mansoori A, Kamel DM. Work-related musculoskeletal disorders among employees with different tasks: an Ahlia University case study. Physiotherapy Quarterly. 2022;30[4]:59-64. doi:10.5114/pq.2022.121146
2. Nasaif H, Alaradi M, Hammam RAA, Bucheeri M, Abdulla M, Abdulla H. Prevalence of self-reported musculoskeletal symptoms among nurses: a multicenter cross-sectional study in Bahrain. International Journal of Occupational Safety and Ergonomics. 2022;29[1]:192-198. doi:10.1080/10803548.2021.2025315
3. Matlab M, Cowman S, Al-Shagag A. Needle Stick Injuries and Compliance among Doctors and Nurses. Bahrain Medical Bulletin. 2017;39[4]:225-228. doi:10.12816/0047772
4. Almoosa A, Asal A, Atif M. Occupational Eye Injury : The neglected role of eye protection. Bahrain Medical Bulletin. 2017;39[2]:82-84. doi:10.12816/0047525
5. Alsayyad AS, Hamadeh RR. The Burden of climate-related conditions among laborers at Al-Razi Health Centre, Bahrain. Journal of the Bahrain Medical Society. 2014;25[1]. doi:10.26715/jbms.p25_2
6. Madani IM, Khalfan S, Khalfan HA, Jidah J, Aladin MN. Occupational exposure to carbon monoxide during charcoal meat grilling. Science of the Total Environment. 1992;114:141-147. doi:10.1016/0048-9697[92]90420-w
7. Al-Nooh AA, Alajmi AAA, Wood D. The Prevalence of Cardiovascular Disease Risk Factors among Employees in the Kingdom of Bahrain between October 2010 and March 2011: A Cross-Sectional Study from a Workplace Health Campaign. Cardiology Research and Practice. 2014;2014:1-9. doi:10.1155/2014/832421
8. Shikdar AA. Identification of ergonomic issues that affect workers in oilrigs in desert environments. International Journal of Occupational Safety and Ergonomics. 2004;10[2]:169-177. doi:10.1080/10803548.2004.11076605
9. Shikdar AA, Al-Kindi M. Office Ergonomics: Deficiencies in computer Workstation design. International Journal of Occupational Safety and Ergonomics. 2007;13[2]:215-223. doi:10.1080/10803548.2007.11076722
10. Palathoti S, Aghbari AHMA, Otitolaiye VO. Effect of long extended working hours on the occupational health and safety of oil and gas workers in the Sultanate of Oman. International Journal of Occupational Safety and Health. 2023;13[4]:419-428. doi:10.3126/ijosh.v13i4.48968
11. Shikdar AA, Khadem M, Al-Harthy S. An ergonomics intervention study of reducing health complaints among office employees. 2008 IEEE International Conference on Industrial Engineering and Engineering Management. Published online December 1, 2008. doi:10.1109/ieem.2008.4738215
12. Dhar D. Retrospective study of injuries in military parachuting. Medical Journal Armed Forces India. 2007;63[4]:353-355. doi:10.1016/s0377-1237[07]80014-1
13. Manjunath GT, Aravindhakshan R, Varghese S. Effect of fasting during Ramadan on thermal stress parameters. Eastern Mediterranean Health Journal. 2019;25[1]:34-39. doi:10.26719/emhj.18.013
14. Al-Rubaee FR, Al-Maniri A. Work Related Injuries in an Oil field in Oman. Oman Medical Journal. 2011;26[5]:315-318. doi:10.5001/omj.2011.79
15. Al-Aamria M, Al-Balushia N, Bailey DL. Estimation of radiation exposure to workers during [18F] FDG PET/CT procedures at Molecular Imaging Center, Oman. Journal of Medical Imaging and Radiation Sciences. 2019;50[4]:565-570. doi:10.1016/j.jmir.2019.05.009
16. Bouchareb Y, Al-Maimani N, Al-Maskery I, et al. ASSESSMENT OF OCCUPATIONAL RADIATION DOSES IN DIFFERENT DIAGNOSTIC, INTERVENTIONAL AND THERAPEUTIC RADIOLOGY AND MOLECULAR IMAGING SERVICES IN OMAN. Radiation Protection Dosimetry. 2021;197[1]:36-45. doi:10.1093/rpd/ncab152
17. Bouchareb Y, Al-Mabsali J, Al-Zeheimi H, Al-Jabri A, Tag N, Al-Dhuhli H. Evaluation of institutional whole-body and extremity occupational radiation doses in nuclear medicine. Radiation Protection Dosimetry. 2023;199[19]:2318-2327. doi:10.1093/rpd/ncad235
18. Al-Harthy NA, Abugad H, Zabeeri N, Alghamdi A, Yousif GFA, Darwish MA. Noise Mapping, Prevalence and Risk Factors of Noise-Induced Hearing Loss among Workers at Muscat International Airport. International Journal of Environmental Research and Public Health. 2022;19[13]:7952. doi:10.3390/ijerph19137952
19. Ramakrishnan, H. K., Pallavi, N., & Doolgindachbaporn, T. Occupational Health Hazard Identification and Risk Mitigation at Engineering Procurement and Construction Projects: Sultanate of Oman. EnvironmentAsia. 2022; 15[Special issue]: 56–67. https://doi.org/10.14456/ea.2022.22
20. Esechie JO, Ibitayo OO. Pesticide use and related health problems among greenhouse workers in Batinah Coastal Region of Oman. Journal of Forensic and Legal Medicine. 2011;18[5]:198-203. doi:10.1016/j.jflm.2011.02.009
21. Williams RJ, Al-Busaidy S, Mehta F, et al. Crimean‐Congo haemorrhagic fever: a seroepidemiological and tick survey in the Sultanate of Oman. Tropical Medicine & International Health. 2000;5[2]:99-106. doi:10.1046/j.1365-3156.2000.00524.x
22. Almayahi ZK. Failure of doxycycline postexposure prophylaxis in human brucellosis contracted through needlestick injury. Infectious Diseases in Clinical Practice. 2019;27[5]:301-302. doi:10.1097/ipc.0000000000000768
23. Ellingsen T, Bener A, Gehani AA. Study of shift work and risk of coronary events. Journal of the Royal Society for the Promotion of Health. 2007;127[6]:265-267. doi:10.1177/1466424007083702
24. Abolfotouh SM, Mahmoud KM, Faraj K, Moammer G, ElSayed A, Abolfotouh MA. Prevalence, consequences and predictors of low back pain among nurses in a tertiary care setting. International Orthopaedics. 2015;39[12]:2439-2449. doi:10.1007/s00264-015-2900-x
25. Hanna F, Daas RN, El-Shareif TJ, Al-Marridi HH, Al-Rojoub ZM, Adegboye OA. The relationship between sedentary behavior, back pain, and psychosocial correlates among university employees. Frontiers in Public Health. 2019;7. doi:10.3389/fpubh.2019.00080
26. Shah SFUH, Bener A, Al‐Kaabi S, Khal ALA, Samson S. The epidemiology of needle stick injuries among health care workers in a newly developed country. Safety Science. 2006;44[5]:387-394. doi:10.1016/j.ssci.2005.11.002
27. Bener A, Rahman YSA, Aleem EYA, Khalid MK. Trends and Characteristics of Head and Neck Injury from Falls: A hospital based study, Qatar. PubMed. Published online May 1, 2011. https://pubmed.ncbi.nlm.nih.gov/21969897
28. Bener A, Rahman YSA, Aleem EYA, Khalid MK. Trends and characteristics of injuries in the State of Qatar: hospital-based study. International Journal of Injury Control and Safety Promotion. 2012;19[4]:368-372. doi:10.1080/17457300.2012.656314
29. Pradhan B, Kjellström T, Atar D, et al. Heat stress impacts on cardiac mortality in Nepali migrant workers in Qatar. Cardiology. 2019;143[1-2]:37-48. doi:10.1159/000500853
30. Razzakh SS, Qureshi MFH. Needlestick injuries among healthcare personnel in Qatar: A retrospective study. Qatar Medical Journal. 2021;2021[2]. doi:10.5339/qmj.2021.35
31. Marji T, Syed MA. Primary care dental professionals’ experiences of sharp injuries in Qatar: A cross-sectional study. Frontiers in Oral Health. 2022;3. doi:10.3389/froh.2022.1014004
32. Consunji R, El‐Menyar A, Hirani N, et al. Work-related injuries in Qatar for 1 year: an initial report from the work-related injury unified registry for Qatar. Qatar Medical Journal. 2022;2022[4]. doi:10.5339/qmj.2022.58
33. Shomar B, Al‐Saad K, Nriagu JO. Mishandling and exposure of farm workers in Qatar to organophosphate pesticides. Environmental Research. 2014;133:312-320. doi:10.1016/j.envres.2014.06.010
34. Mochtar IA, Rw H. Assessment of the 10-year risk of coronary heart disease events for Qatar Petroleum’s firefighters and non-firefighter staff in Qatar. Eastern Mediterranean Health Journal. 2012;18[2]:127-131. doi:10.26719/2012.18.2.127
35. Al‐Thani H, El‐Menyar A, Consunji R, et al. Epidemiology of occupational injuries by nationality in Qatar: Evidence for focused occupational safety programmes. Injury-International Journal of the Care of the Injured. 2015;46[9]:1806-1813. doi:10.1016/j.injury.2015.04.023
36. Shehab D, Al-Jarallah K, Moussa M, Adham N. Prevalence of Low Back Pain among Physical Therapists in Kuwait. Medical Principles and Practice. 2003;12[4]:224-230. doi:10.1159/000072288
37. Landry MD, Raman SR, Sulway C, Golightly YM, Hamdan E. Prevalence and risk factors associated with low back pain among health care providers in a Kuwait hospital. Spine. 2008;33[5]:539-545. doi:10.1097/brs.0b013e3181657df7
38. Alrowayeh HN, Alshatti TA, Aljadi SH, Fares M, Alshamire MM, Alwazan SS. Prevalence, characteristics, and impacts of work-related musculoskeletal disorders: a survey among physical therapists in the State of Kuwait. BMC Musculoskeletal Disorders. 2010;11[1]. doi:10.1186/1471-2474-11-116
39. Raman SR, Al-Halabi B, Hamdan E, Landry MD. Prevalence and risk factors associated with self-reported carpal tunnel syndrome [CTS] among office workers in Kuwait. BMC Research Notes. 2012;5[1]. doi:10.1186/1756-0500-5-289
40. Alnaser MZ, Aljadi SH. Physical therapists with work-related musculoskeletal disorders in the State of Kuwait: A comparison across countries and health care professions. Work-a Journal of Prevention Assessment & Rehabilitation. 2019;63[2]:261-268. doi:10.3233/wor-192927
41. Alnaser MZ, Almaqsied AM, Alshatti SA. Risk factors for work-related musculoskeletal disorders of dentists in Kuwait and the impact on health and economic status. Work-a Journal of Prevention Assessment & Rehabilitation. 2021;68[1]:213-221. doi:10.3233/wor-203369
42. Fm EA. Assessment of radium-226 in two cases of radium luminizers using whole-body counting. Health Physics. 1982;43[4]:567-572. doi:10.1097/00004032-198210000-00010
43. Mustafa A, Sabol J, Janeczek J. Doses from Occupational Exposure. Health Physics. 1985;49[6]:1197-1204. doi:10.1097/00004032-198512000-00016
44. Al-Abdulsalam A, Brindhaban A. Occupational Radiation Exposure among the Staff of Departments of Nuclear Medicine and Diagnostic Radiology in Kuwait. Medical Principles and Practice. 2013;23[2]:129-133. doi:10.1159/000357123
45. Omar A, Abdo NM, Salama MF, Al-Mousa HH. Occupational Injuries Prone to Infectious Risks amongst Healthcare Personnel in Kuwait: A Retrospective Study. Medical Principles and Practice. 2014;24[2]:123-128. doi:10.1159/000369462
46. Alnaaimi M, Alkhorayef M, Omar MM, et al. Occupational radiation exposure in nuclear medicine department in Kuwait. Radiation Physics and Chemistry. 2017;140:233-236. doi:10.1016/j.radphyschem.2017.02.048
47. Alnaaimi M, Alduaij M, Shenawy F, et al. Assessment of eye doses to staff involved in interventional cardiology procedures in Kuwait. Radiation and Environmental Biophysics. 2021;60[4]:639-645. doi:10.1007/s00411-021-00929-3
48. Yassin MF, Almutairi H, Alhajeri NS, Al-Fadhli FM, Al-Rashidi M, Shatti T. Assessment of noise exposure and associated health risk in school environment. International Journal of Environmental Science and Technology. 2016;13[8]:2011-2024. doi:10.1007/s13762-016-1035-y
49. Buqammaz M, Gasana J, Alahmad B, Shebl M, Albloushi D. Occupational Noise-Induced Hearing Loss among Migrant Workers in Kuwait. International Journal of Environmental Research and Public Health. 2021;18[10]:5295. doi:10.3390/ijerph18105295
50. Mohamed IY. Asbestos-cement pneumoconiosis: First surgically confirmed case in kuwait. American Journal of Industrial Medicine. 1990;17[2]:241-245. doi:10.1002/ajim.4700170208
51. El-Sonbaty, Abdul-Ghaffar NU. Hypokalaemic paralysis precipitated by working in hot weather. Tropical Doctor. 1995;25[2]:64-66. doi:10.1177/004947559502500205
52. Alahmad B, Al-Hemoud A, Al-Bouwarthan M, et al. Extreme heat and work injuries in Kuwait’s hot summers. Occupational and Environmental Medicine. 2023;80[6]:347-352. doi:10.1136/oemed-2022-108697
53. Kanan MW. CEMENT DERMATITIS AND ATMOSPHERIC PARAMETERS IN KUWAIT*. British Journal of Dermatology. 1972;86[2]:155-159. doi:10.1111/j.1365-2133.1972.tb16079.x
54. Al-Shatti AKS, El-Desouky MA, Zaki RM, Al-Azem MA, Al-Lagani M. Health care for pesticide applicators in a locust eradication campaign in Kuwait [1988–1989]. Environmental Research. 1997;73[1-2]:219-226. doi:10.1006/enrs.1997.3735
55. Al-Ayyadhi N, Akhtar S. Prevalence and Risk Factors Associated with Self-Rated Morbidities Among South Asian Migrant Gas Station Workers in Kuwait. Journal of Immigrant and Minority Health. 2018;20[6]:1324-1331. doi:10.1007/s10903-018-0701-1
56. Hashim R, Salah A, Mayahi F, Haidary S. Prevalence of postural musculoskeletal symptoms among dental students in United Arab Emirates. BMC Musculoskeletal Disorders. 2021;22[1]. doi:10.1186/s12891-020-03887-x
57. Al‐Rawi NH, Yousef H, Khamis M, Belkadi O, Ahmed S, Ali S. Vertebral Malalignment among Male Dentists with Workrelated Musculoskeletal Pain in the United Arab Emirates. The Journal of Contemporary Dental Practice. 2018;19[7]:773-777. doi:10.5005/jp-journals-10024-2335
58. Al‐Rawi NH, Khatib HE, Lin R, et al. Work-related Musculoskeletal Pain among Different Dental Specialists in United Arab Emirates. The Journal of Contemporary Dental Practice. 2016;17[8]:639-644. doi:10.5005/jp-journals-10024-1904
59. Hussein A, Mando M, Radišauskas R. Work-Related Musculoskeletal Disorders among Dentists in the United Arab Emirates: A Cross-Sectional Study. Medicina-lithuania. 2022;58[12]:1744. doi:10.3390/medicina58121744
60. Elshami W, Abuzaid MM, Pekkarinen A, Kortesniemi M. ESTIMATION OF OCCUPATIONAL RADIATION EXPOSURE FOR MEDICAL WORKERS IN RADIOLOGY AND CARDIOLOGY IN THE UNITED ARAB EMIRATES: NINE HOSPITALS EXPERIENCE. Radiation Protection Dosimetry. 2020;189[4]:466-474. doi:10.1093/rpd/ncaa060
61. AlMahmoud T, Elkonaisi I, Grivna M, AlNuaimi G, Abu‐Zidan FM. Eye Injuries and Related Risk Factors among Workers in Small-scale Industrial Enterprises. Ophthalmic Epidemiology. 2020;27[6]:453-459. doi:10.1080/09286586.2020.1770302
62. Grivna M, Eid HO, Abu‐Zidan FM. Injuries from falling objects in the United Arab Emirates. International Journal of Injury Control and Safety Promotion. 2013;22[1]:68-74. doi:10.1080/17457300.2013.863784
63. Ahmed H. Noise exposure, awareness, practice and noise annoyance among steel workers in United Arab Emirates. The Open Public Health Journal. 2012;5[1]:28-35. doi:10.2174/1874944501205010028
64. Elmehdi HM, Emirates UA. Noise levels in UAE Dental clinics: Health impact on dental healthcare professionals. Public Health Frontier. Published online December 24, 2013:189-192. doi:10.5963/phf0204002
65. Nassar M, Islam MS, D’souza S, et al. Tinnitus Prevalence and Associated Factors among Dental Clinicians in the United Arab Emirates. International Journal of Environmental Research and Public Health. 2023;20[2]:1403. doi:10.3390/ijerph20021403
66. Jacob A, Newson-Smith M, Murphy EA, Steiner M, Dick F. Sharps injuries among health care workers in the United Arab Emirates. Occupational Medicine. 2010;60[5]:395-397. doi:10.1093/occmed/kqq039
67. Bener A, Almehdi AM, Alwash R, Al-Neamy FRM. A pilot survey of blood lead levels in various types of workers in the United Arab Emirates. Environment International. 2001;27[4]:311-314. doi:10.1016/s0160-4120[01]00061-7
68. Bener A, Obineche EN, Gillett MPT, Pasha M, Bishawi B. Association between blood levels of lead, blood pressure and risk of diabetes and heart disease in workers. International Archives of Occupational and Environmental Health. 2001;74[5]:375-378. doi:10.1007/s004200100231
69. Ahmed H, Abdullah AA. Dust Exposure and Respiratory Symptoms among Cement Factory Workers in the United Arab Emirates. Industrial Health. 2012;50[3]:214-222. doi:10.2486/indhealth.ms1320
70. Gomes J, Lloyd O, Revitt DM, Norman J. Erythrocyte cholinesterase activity levels in desert farm workers. Occupational Medicine. 1997;47[2]:90-94. doi:10.1093/occmed/47.2.90
71. Abou-Taleb ANM, Musaiger AO, Abdelmoneim RB. Health status of cement workers in the United Arab Emirates. The Journal of the Royal Society of Health. 1995;115[6]:378-381. doi:10.1177/146642409511500610
72. Lotah HNA, Agarwal AK, Khanam R. Heavy metals in hair and nails as markers of occupational hazard among welders working in United Arab Emirates. Toxicological Research. 2021;38[1]:63-68. doi:10.1007/s43188-021-00091-4
73. Al-Neamy FRM, Almehdi AM, Alwash R, Pasha MA, Ahmad I, Bener A. Occupational lead exposure and amino acid profiles and liver function tests in industrial workers. International Journal of Environmental Health Research. 2001;11[2]:181-188. doi:10.1080/09603120020047564
74. Beshwari MMM, Bener A, Ameen A, Almehdi AM, Ouda HZ, Pasha MA. Pesticide-related health problems and diseases among farmers in the United Arab Emirates. International Journal of Environmental Health Research. 1999;9[3]:213-221. doi:10.1080/09603129973182
75. Al-Neaimi YI, Gomes J, Lloyd O. Respiratory illnesses and ventilatory function among workers at a cement factory in a rapidly developing country. Occupational Medicine. 2001;51[6]:367-373. doi:10.1093/occmed/51.6.367
76. Bener A, Brebner JA, Atta MNS, Gomes J, Ozkaragoz F, Cheema M. Respiratory symptoms and lung function in taxi drivers and manual workers. Aerobiologia. 1997;13[1]:11-15. doi:10.1007/bf02694785
77. Bener A, Galadari I, Al-Mutawa JK, Al-Maskari F, Das M, Abuzeid MSO. Respiratory symptoms and lung function in garage workers and taxi drivers. Journal of the Royal Society for the Promotion of Health. 1998;118[6]:346-353. doi:10.1177/146642409811800613
78. Bener A, Lestringant GG, Beshwari MMM, Pasha MA. Respiratory symptoms, skin disorders and serum IgE levels in farm workers. PubMed. 1999;31[2]:52-56. https://pubmed.ncbi.nlm.nih.gov/10219428
79. Dafalla AIA, Almuhairi SASO, AlHosani MHJ, et al. Intestinal parasitic infections among expatriate workers in various occupations in Sharjah, United Arab Emirates. Revista Do Instituto De Medicina Tropical De Sao Paulo. 2017;59[0]. doi:10.1590/s1678-9946201759082
80. Al-Ali K, Hashim R. Occupational health problems of dentists in the United Arab Emirates. International Dental Journal. 2012;62[1]:52-56. doi:10.1111/j.1875-595x.2011.00091.x
81. Salem AM, Jaumally BA, Bayanzay K, Khoury KE, Torkaman A. Traumatic brain injuries from work accidents: a retrospective study. Occupational Medicine. 2013;63[5]:358-360. doi:10.1093/occmed/kqt037
82. Ka AW, Almas K, Shethri A, Mq AQ. Back &amp; Neck Problems Among Dentists and Dental Auxiliaries. The Journal of Contemporary Dental Practice. 2001;2[3]:1-10. doi:10.5005/jcdp-2-3-1
83. Meo SA, Alsaaran ZF, Alshehri M, et al. Work-Related musculoskeletal symptoms among building construction workers in Riyadh, Saudi Arabia. Pakistan Journal of Medical Sciences. 2013;29[6]. doi:10.12669/pjms.296.4052
84. Behisi M, Al-Otaibi ST, Beach J. Back pain among health care workers in a Saudi Aramco facility: prevalence and associated factors. Archives of Environmental & Occupational Health. 2013;68[1]:30-38. doi:10.1080/19338244.2011.627895
85. Attar SM. Frequency and risk factors of musculoskeletal pain in nurses at a tertiary centre in Jeddah, Saudi Arabia: a cross sectional study. BMC Research Notes. 2014;7[1]. doi:10.1186/1756-0500-7-61
86. Alghadir AH, Anwer S. Prevalence of musculoskeletal pain in construction workers in Saudi Arabia. The Scientific World Journal. 2015;2015:1-5. doi:10.1155/2015/529873
87. Aljanakh M, Shaikh S, Siddiqui AA, Al-Mansour M, Hassan SS. Prevalence of musculoskeletal disorders among dentists in the Ha’il Region of Saudi Arabia. Annals of Saudi Medicine. 2015;35[6]:456-461. doi:10.5144/0256-4947.2015.456
88. Al-Juhani MAM, Khandekar R, Al-Harby M, Al-Hassan A, Edward DP. Neck and upper back pain among eye care professionals. Occupational Medicine. Published online September 28, 2015:kqv132. doi:10.1093/occmed/kqv132
89. Ezzat HM, Al-Sultan A, Al-Shammari A, et al. Prevalence of neck pain among cabin crew of Saudi Airlines. Journal of Back and Musculoskeletal Rehabilitation. 2015;28[3]:425-431. doi:10.3233/bmr-140536
90. Homaid MB, Abdelmoety D, Alshareef W, et al. Prevalence and risk factors of low back pain among operation room staff at a Tertiary Care Center, Makkah, Saudi Arabia: a cross-sectional study. Annals of Occupational and Environmental Medicine. 2016;28[1]. doi:10.1186/s40557-016-0089-0
91. Muaidi QI, Shanb AA. Prevalence causes and impact of work related musculoskeletal disorders among physical therapists. Journal of Back and Musculoskeletal Rehabilitation. 2016;29[4]:763-769. doi:10.3233/bmr-160687
92. Al-Mohrej OA, AlShaalan NS, Al-Bani WM, Masuadi E, Almodaimegh H. Prevalence of musculoskeletal pain of the neck, upper extremities and lower back among dental practitioners working in Riyadh, Saudi Arabia: a cross-sectional study. BMJ Open. 2016;6[6]:e011100. doi:10.1136/bmjopen-2016-011100
93. Alshehri ZY, Al-Zoughool M. Self-reported musculoskeletal symptoms among dentists in Saudi Arabia. Industrial Health. 2017;55[4]:338-344. doi:10.2486/indhealth.2016-0174
94. Al-Shehri Z, Zoughool MA. RETRACTED ARTICLE: Prevalence and risk factors of musculoskeletal symptoms among dental students and dental practitioners in Riyadh City, Saudi Arabia. Archives of Environmental & Occupational Health. 2017;73[1]:56-63. doi:10.1080/19338244.2017.1299085
95. El-Helaly M, Balkhy HH, Vallenius L. Carpal tunnel syndrome among laboratory technicians in relation to personal and ergonomic factors at work. Journal of Occupational Health. 2017;59[6]:513-520. doi:10.1539/joh.16-0279-oa
96. Alghadir AH, Zafar H, Iqbal ZA, Al-Eisa E. Work-Related low back pain among physical therapists in Riyadh, Saudi Arabia. Workplace Health & Safety. 2017;65[8]:337-345. doi:10.1177/2165079916670167
97. Al-Rammah TY, Aloufi AS, Algaeed SK, Alogail NS. The prevalence of work-related musculoskeletal disorders among sonographers. Work-a Journal of Prevention Assessment & Rehabilitation. 2017;57[2]:211-219. doi:10.3233/wor-172558
98. Al-Shagga MA, Ibrahim IAA, Shahzad N, Al-Dubai SAR. PREVALENCE AND ASSOCIATED FACTORS OF MUSCOSKELETAL DISORDERS AMONG ARABIC CALLIGRAPHERS LIVING IN SAUDI... Malaysian Journal of Public Health Medicine. 2018;18[2]:39-44.
99. Al-Salameen AH, Abugad H, Al-Otaibi ST. Low back pain among workers in a paint factory. Saudi Journal of Medicine and Medical Sciences. 2019;7[1]:33. doi:10.4103/sjmms.sjmms_81_17
100. Alnaami I, Awadalla NJ, Alkhairy MA, et al. Prevalence and factors associated with low back pain among health care workers in southwestern Saudi Arabia. BMC Musculoskeletal Disorders. 2019;20[1]. doi:10.1186/s12891-019-2431-5
101. Alnefaie M, Alamri AA, Hariri AF, et al. Musculoskeletal Symptoms Among Surgeons at a Tertiary Care Center: a Survey Based Study. Medicinski Arhiv. 2019;73[1]:49. doi:10.5455/medarh.2019.73.49-54
102. Alhusain F, Almohrij M, Althukeir F, et al. Prevalence of carpal tunnel syndrome symptoms among dentists working in Riyadh. Annals of Saudi Medicine. 2019;39[2]:104-111. doi:10.5144/0256-4947.2019.07.03.1405
103. Gaowgzeh RA. Low back pain among nursing professionals in Jeddah, Saudi Arabia: Prevalence and risk factors. Journal of Back and Musculoskeletal Rehabilitation. 2019;32[4]:555-560. doi:10.3233/bmr-181218
104. Abdel-Salam DM, Almuhaisen AS, Alsubiti RA, et al. Musculoskeletal pain and its correlates among secondary school female teachers in Aljouf region, Saudi Arabia. Journal of Public Health. 2019;29[2]:303-310. doi:10.1007/s10389-019-01127-8
105. Shammari MA, Hassan A, Dandan OA, Gadeeb MA, Bubshait D. Musculoskeletal symptoms among radiologists in Saudi Arabia: a multi-center cross-sectional study. BMC Musculoskeletal Disorders. 2019;20[1]. doi:10.1186/s12891-019-2933-1
106. Aboalshamat K. Nordic assessment of occupational disorders among dental students and dentists in Saudi Arabia. Journal of International Society of Preventive and Community Dentistry. 2020;10[5]:561. doi:10.4103/jispcd.jispcd_142_20
107. AlNekhilan AF, AlTamimi AM, Alaqeel BY, AlHawery AA, Alfadhel S, Masuadi E. Work-related musculoskeletal disorders among clinical laboratory workers. Avicenna Journal of Medicine. 2020;10[1]:29. doi:10.4103/ajm.ajm_67_19
108. Dalboh A, Alshehri NA, Alrafie AA, Bakri KA. Prevalence and awareness of varicose veins among teachers in Abha, Saudi Arabia. Journal of Family Medicine and Primary Care. 2020;9[9]:4784. doi:10.4103/jfmpc.jfmpc_490_20
109. Tariah HA, Nafai S, Alajmi M, Almutairi FM, Alanazi B. Work-related musculoskeletal disorders in nurses working in the Kingdom of Saudi Arabia. Work-a Journal of Prevention Assessment & Rehabilitation. 2020;65[2]:421-428. doi:10.3233/wor-203094
110. Algarni FS, Kachanathu SJ, AlAbdulwahab SS. A Cross-Sectional Study on the Association of Patterns and Physical Risk Factors with Musculoskeletal Disorders among Academicians in Saudi Arabia. BioMed Research International. 2020;2020:1-7. doi:10.1155/2020/8930968
111. Kaleem SM, Asif SM, Kota MZ, Alam T, Assiri H, Zakirulla M. Ergonomic Considerations in the incidence of CTS in College of Dentistry, King Khalid University, Abha - Kingdom of Saudi Arabia. PubMed. 2020;18[1]:277-285. doi:10.3290/j.ohpd.a44031
112. Elmannan AAA, AlHindi HA, AlBaltan RI, et al. Non-specific low back pain among nurses in Qassim, Saudi Arabia. Cureus. Published online November 15, 2021. doi:10.7759/cureus.19594
113. Abolfotouh MA, Alomair F, Alangari D, Bushnak I, Aldebasi B, Almansoof AS. Epidemiology of work-related lower back pain among rehabilitation professionals in Saudi Arabia. Eastern Mediterranean Health Journal. 2021;27[4]:390-398. doi:10.26719/emhj.21.019
114. Althomali OW, Amin J, Alghamdi W, Shaik DH. Prevalence and Factors Associated with Musculoskeletal Disorders among Secondary Schoolteachers in Hail, Saudi Arabia: A Cross-Sectional Survey. International Journal of Environmental Research and Public Health. 2021;18[12]:6632. doi:10.3390/ijerph18126632
115. Aldukhayel A, Almeathem FK, Aldughayyim AA, et al. Musculoskeletal pain among school teachers in Qassim, Saudi Arabia: prevalence, pattern, and its risk factors. Cureus. Published online August 27, 2021. doi:10.7759/cureus.17510
116. Aldhafian OR, Alsamari F, Alshahrani NA, et al. Musculoskeletal pain among male faculty members of the College of Medicine and College of Dentistry. Medicine. 2021;100[21]:e26176. doi:10.1097/md.0000000000026176
117. Almaghrabi A, Alsharif F. Prevalence of Low Back Pain and Associated Risk Factors among Nurses at King Abdulaziz University Hospital. International Journal of Environmental Research and Public Health. 2021;18[4]:1567. doi:10.3390/ijerph18041567
118. Kakaraparthi VN, Vishwanathan K, Gadhavi B, et al. The prevalence, characteristics, and impact of work-related musculoskeletal disorders among physical therapists in the Kingdom of Saudi Arabia – a cross-sectional study. Medycyna Pracy. Published online August 27, 2021. doi:10.13075/mp.5893.01114
119. AlOmar RS, AlShamlan NA, Alawashiz S, Badawood Y, Ghwoidi BA, Abugad H. Musculoskeletal symptoms and their associated risk factors among Saudi office workers: a cross-sectional study. BMC Musculoskeletal Disorders. 2021;22[1]. doi:10.1186/s12891-021-04652-4
120. Alhasan AS, Aalam WA. Magnitude and determinants of computer vision syndrome among radiologists in Saudi Arabia: a national survey. Academic Radiology. 2022;29[9]:e197-e204. doi:10.1016/j.acra.2021.10.023
121. Alhakami AM, Madkhli A, Ghareeb M, et al. The Prevalence and Associated Factors of Neck Pain among Ministry of Health Office Workers in Saudi Arabia: A Cross Sectional Study. Healthcare. 2022;10[7]:1320. doi:10.3390/healthcare10071320
122. Althomali OW. Long-Term Prevalence and Risk Factors of Musculoskeletal Disorders among the Schoolteachers in Hail, Saudi Arabia: A Cross-Sectional Study. BioMed Research International. 2022;2022:1-7. doi:10.1155/2022/3610196
123. Shubayr N, Alashban Y. Musculoskeletal symptoms among radiation technologists in Saudi Arabia: prevalence and causative factors. Acta Radiologica. 2021;63[4]:497-503. doi:10.1177/02841851211004428
124. Alzayani MK, Salama KF, Zafar M. Work-related musculoskeletal disorders among dental staff in Armed Force Hospital in Dhahran, Saudi Arabia. DOAJ [DOAJ: Directory of Open Access Journals]. 2021;12:119. doi:10.4103/ijpvm.ijpvm_136_20
125. Ali SA, Najmi WK, Hakami FM, et al. Prevalence of varicose veins among nurses in different departments in Jazan Public Hospitals, Saudi Arabia: a Cross-Sectional study. Cureus. Published online April 25, 2022. doi:10.7759/cureus.24462
126. AlMaghlouth MK, Alserhani NM, Aldossary FA, Alabdulqader MA, Al-Dhafer BA. Prevalence, patterns, and risk factors of Work-Related Musculoskeletal Diseases among teachers in the Eastern Province, Saudi Arabia: A Community-Based Retrospective Cross-Sectional Survey. Cureus. Published online December 4, 2022. doi:10.7759/cureus.32178
127. Alghadir AH, Khalid S, Iqbal ZA. Work-related musculoskeletal disorders among information technology professionals in Riyadh, Saudi Arabia. Medycyna Pracy. 2022;73[5]:397-406. doi:10.13075/mp.5893.01281
128. Alzahrani AS, Baatiyyah EA, Bakry S, et al. The prevalence of back pain among male teachers in Makkah region, Saudi Arabia: An analytic cross-sectional study. Medical Science. 2022;26[125]:1-9. doi:10.54905/disssi/v26i125/ms318e2326
129. Saikhan LA. Prevalence, characteristics, consequences, and awareness of work-related musculoskeletal pain among cardiac sonographers compared with other healthcare workers in Saudi Arabia: A cross sectional study. PLOS ONE. 2023;18[5]:e0285369. doi:10.1371/journal.pone.0285369
130. Aldaheri A, Al-Juhani MAM, Aldaheri R. The prevalence and associated factors of lower back pain among surgeons in Makkah Region, Saudi Arabia. Journal of Family Medicine and Primary Care. 2023;12[7]:1308-1314. doi:10.4103/jfmpc.jfmpc_468_22
131. Alelyani M, Gameraddin M, Khushayl AMA, et al. Work-related musculoskeletal symptoms among Saudi radiologists: a cross-sectional multi-centre study. BMC Musculoskeletal Disorders. 2023;24[1]. doi:10.1186/s12891-023-06596-3
132. AlSahiem J, Alghamdi S, AlQahtani R, et al. Musculoskeletal disorders among dental students: a survey from Saudi Arabia. BMC Oral Health. 2023;23[1]. doi:10.1186/s12903-023-03469-y
133. Al-Haj AN, Lobriguito AM, Al-Gain I. Staff eye doses in a large medical centre in Saudi Arabia: are they meeting the new ICRP recommendations? Radiation Protection Dosimetry. 2015;165[1-4]:294-298. doi:10.1093/rpd/ncv073
134. Salama KF, AlObireed A, AlBagawi M, AlSufayan Y, AlSerheed M. Assessment of occupational radiation exposure among medical staff in health-care facilities in the Eastern Province, Kingdom of Saudi Arabia. Indian Journal of Industrial Medicine. 2016;20[1]:21. doi:10.4103/0019-5278.183832
135. Alashrah S, El-Taher A. Assessing Exposure Hazards and Metal Analysis Resulting from Bauxite Samples Collected from a Saudi Arabian Mine. Polish Journal of Environmental Studies. 2018;27[3]:959-966. doi:10.15244/pjoes/76177
136. Alkhorayef M, Sulieman A, Mohamed‐Ahmed MM, et al. Staff and ambient radiation dose resulting from therapeutic nuclear medicine procedures. Applied Radiation and Isotopes. 2018;141:270-274. doi:10.1016/j.apradiso.2018.07.014
137. Shhub AN. Monitoring radiation exposure of Saudi Aramco pilots. Health Physics. 2019;118[2]:162-169. doi:10.1097/hp.0000000000001162
138. Alkhorayef M, Mayhoub FH, Salah H, et al. Assessment of occupational exposure and radiation risks in nuclear medicine departments. Radiation Physics and Chemistry. 2020;170:108529. doi:10.1016/j.radphyschem.2019.108529
139. Shubayr N, Alashban Y, Almalki M, Aldawood S, Aldosari A. Occupational radiation exposure among diagnostic radiology workers in the Saudi ministry of health hospitals and medical centers: A five-year national retrospective study. Journal of King Saud University - Science. 2021;33[1]:101249. doi:10.1016/j.jksus.2020.101249
140. Alashban Y, Shubayr N, Almalki M, Albeshan S, Aldawood S, Aldosari A. Assessment of radiation dose for dental workers in Saudi Arabia [2015–2019]. Journal of King Saud University - Science. 2021;33[1]:101250. doi:10.1016/j.jksus.2020.101250
141. Al-Mohammed HI, Sulieman A, Mayhoub FH, et al. Occupational exposure and radiobiological risk from thyroid radioiodine therapy in Saudi Arabia. Scientific Reports. 2021;11[1]. doi:10.1038/s41598-021-93342-1
142. Johary YH, Aamry A, Albarakati S, et al. Staff radiation exposure at four radiology departments in the Aseer region of Saudi Arabia. Radiation Physics and Chemistry. 2022;200:110302. doi:10.1016/j.radphyschem.2022.110302
143. Khouqeer GA, Sulieman A, Mayhoub FH, et al. Staff occupational exposure and ambient doses resulting from patients undergoing nuclear medicine procedures’. Radiation Physics and Chemistry. 2023;212:111091. doi:10.1016/j.radphyschem.2023.111091
144. Omer H, Salah H, Tamam N, et al. Assessment of occupational exposure from PET and PET/CT scanning in Saudi Arabia. Radiation Physics and Chemistry. 2023;204:110642. doi:10.1016/j.radphyschem.2022.110642
145. Shanks NJ, Al-Kalai D. Occupation risk of needlestick injuries among health care personnel in Saudi Arabia. Journal of Hospital Infection. 1995;29[3]:221-226. doi:10.1016/0195-6701[95]90332-1
146. Memish ZA, Almuneef M, Dillon JAR. Epidemiology of needlestick and sharps injuries in a tertiary care center in Saudi Arabia. American Journal of Infection Control. 2002;30[4]:234-241. doi:10.1067/mic.2002.118841
147. Ghamdi SA, Al-Azraqi TA, Bello CSS, Gutierrez H, Hyde MR, Abdullah M. Needlestick and sharps injuries at Asir Central Hospital, Abha, Saudi Arabia. Annals of Saudi Medicine. 2003;23[6]:404-407. doi:10.5144/0256-4947.2003.404
148. Jahan S. Epidemiology of needlestick injuries among health care workers in a secondary care hospital in Saudi Arabia. Annals of Saudi Medicine. 2005;25[3]:233-238. doi:10.5144/0256-4947.2005.233
149. Mahfouz AA, Abdelmoneim I, Khan M, et al. Injection safety at primary health care level in south-western Saudi Arabia. Eastern Mediterranean Health Journal. 2009;15[2]:443-450. doi:10.26719/2009.15.2.443
150. El-Hazmi M, Almajid F. Needle stick and sharps injuries among health care workers: A 5-year surveillance in a teaching center in... ResearchGate. Published online May 1, 2008. https://www.researchgate.net/publication/228639578
151. Memish ZA, Assiri AM, Eldalatony MM, Hathout H, Alzoman H, Undaya M. Risk analysis of needle stick and sharp object injuries among health care workers in a tertiary care hospital [Saudi Arabia]. Journal of Epidemiology and Global Health. 2013;3[3]:123. doi:10.1016/j.jegh.2013.03.004
152. Khabour OF, Ali KHA, Mahallawi WH. Occupational infection and needle stick injury among clinical laboratory workers in Al-Madinah city, Saudi Arabia. Journal of Occupational Medicine and Toxicology. 2018;13[1]. doi:10.1186/s12995-018-0198-5
153. Samargandy S, Bukhari LM, Samargandy S, et al. Epidemiology and clinical consequences of occupational exposure to blood and other body fluids in a university hospital in Saudi Arabia. Saudi Medical Journal. 2016;37[7]:783-790. doi:10.15537/smj.2016.7.14261
154. Aldakhil L, Yenugadhati N, Al-Seraihi O, Al-Zoughool M. Prevalence and associated factors for needlestick and sharp injuries [NSIs] among dental assistants in Jeddah, Saudi Arabia. Environmental Health and Preventive Medicine. 2019;24[1]. doi:10.1186/s12199-019-0815-7
155. Makeen AM, Alharbi A, Mahfouz MS, et al. Needlestick and sharps injuries among secondary and tertiary healthcare workers, Saudi Arabia. Nursing Open. 2021;9[1]:816-823. doi:10.1002/nop2.1136
156. Fadil RA, Abdelmutalab NA, Abdelhafeez SA, et al. Pattern and risk factors of sharp object injuries among health care workers in two tertiary hospitals, Al Taif-Kingdom of Saudi Arabia 2016–2018. Saudi Journal of Biological Sciences. 2021;28[11]:6582-6585. doi:10.1016/j.sjbs.2021.07.031
157. Abalkhail A, Kabir R, Elmosaad YM, et al. Needle-Stick and Sharp Injuries among Hospital Healthcare Workers in Saudi Arabia: A Cross-Sectional Survey. International Journal of Environmental Research and Public Health. 2022;19[10]:6342. doi:10.3390/ijerph19106342
158. Alharazi R, Almutary H, Felemban O, et al. Prevalence of needle stick injuries among nurses in Jeddah, Saudi Arabia. Nursing. 2022;Volume 12:235-246. doi:10.2147/nrr.s376343
159. Al-Shehri S, Kayal M, Almshhad HA, et al. The incidence of needlestick and sharps injuries among healthcare workers in a tertiary care hospital: a Cross-Sectional study. Cureus. Published online April 25, 2023. doi:10.7759/cureus.38097
160. Ballal SG. Ocular trauma in an iron forging industry in the Eastern Province, Saudi Arabia. Occupational Medicine. 1997;47[2]:77-80. doi:10.1093/occmed/47.2.77
161. Ali BA, Ballal SG, Albar AA, Ahmed H. Post-shift changes in pulmonary function in a cement factory in eastern Saudi Arabia. Occupational Medicine. 1998;48[8]:519-522. doi:10.1093/occmed/48.8.519
162. Al-Dawood K. Non-fatal occupational injuries admitted to hospitals among general organization for social insurance workers in Al-Khobar city, Saudi Arabia: Experience of one year. Journal of Family and Community Medicine. 2000;7[2]:35. doi:10.4103/2230-8229.98179
163. Ahmed H, Dennis J, Badran OB, et al. Occupational noise exposure and hearing loss of workers in two plants in eastern Saudi Arabia. Annals of Occupational Hygiene. Published online July 1, 2001. doi:10.1093/annhyg/45.5.371
164. Noweir MH, Jamil A. Noise pollution in textile, printing and publishing industries in Saudi Arabia. Environmental Monitoring and Assessment. 2003;83[1]:103-111. doi:10.1023/a:1022418805827
165. Noweir MH, Bafail AO. Study of summer heat exposure at the ground services operations of a main international airport in Saudi Arabia. Environmental Monitoring and Assessment. 2007;145[1-3]:103-111. doi:10.1007/s10661-007-0019-2
166. Noweir MH, Zytoon MA. Occupational exposure to noise and hearing thresholds among civilian aircraft maintenance workers. International Journal of Industrial Ergonomics. 2013;43[6]:495-502. doi:10.1016/j.ergon.2013.04.001
167. Noweir MH, Bafail AO, Jomoah IM. Noise pollution in metalwork and woodwork industries in the Kingdom of Saudi Arabia. International Journal of Occupational Safety and Ergonomics. 2014;20[4]:661-670. doi:10.1080/10803548.2014.11077068
168. Ballal SG, Ahmed H, Ali BA, Albar AA, Alhasan AY. Pulmonary Effects of Occupational Exposure to Portland Cement: A Study from Eastern Saudi Arabia. International Journal of Occupational and Environmental Health. 2004;10[3]:272-277. doi:10.1179/oeh.2004.10.3.272
169. Al-Abdulwahhab B, Alduraiby RI, Ahmed MA, et al. Hearing loss and its association with occupational noise exposure among Saudi dentists: a cross-sectional study. BDJ Open. 2016;2[1]. doi:10.1038/bdjopen.2016.6
170. Alsabaani NA, Awadalla NJ, Saq IHA, et al. Occupational ocular incidents in dentists: a multicentre study in southwestern Saudi Arabia. International Dental Journal. 2017;67[6]:371-377. doi:10.1111/idj.12324
171. Al-Bouwarthan M, Quinn M, Kriebel D, Wegman DH. Assessment of Heat Stress Exposure among Construction Workers in the Hot Desert Climate of Saudi Arabia. Annals of Work Exposures and Health. 2019;63[5]:505-520. doi:10.1093/annweh/wxz033
172. Al-Bouwarthan M, Quinn M, Kriebel D, Wegman DH. A field evaluation of construction workers’ activity, hydration status, and heat strain in the extreme summer heat of Saudi Arabia. Annals of Work Exposures and Health. 2020;64[5]:522-535. doi:10.1093/annweh/wxaa029
173. Al-Bouwarthan M, Quinn M, Kriebel D, Wegman DH. Risk of Kidney Injury among Construction Workers Exposed to Heat Stress: A Longitudinal Study from Saudi Arabia. International Journal of Environmental Research and Public Health. 2020;17[11]:3775. doi:10.3390/ijerph17113775
174. Eman AS. Prevalence of Noise Induced Hearing Loss and Other Associated Risk Factors among Saudi Dental Professionals. DOAJ [DOAJ: Directory of Open Access Journals]. Published online August 1, 2020. https://doaj.org/article/ad8528475d3c4671978801243dfa53c6
175. Alsaab F, Alaraifi AK, Alhomaydan WA, Ahmed AZ, Elzubair AG. Hearing impairment in military personnel in Eastern Saudi Arabia. Journal of Family and Community Medicine. 2021;28[2]:110. doi:10.4103/jfcm.jfcm_501_20
176. Razik M, Altuwayhir A, Almihmadi M, et al. Pattern of traumatic occupational injuries in Saudi Arabia: A cross-sectional study. Journal of Family Medicine and Primary Care. 2022;11[12]:7907. doi:10.4103/jfmpc.jfmpc_2009_22
177. Ballal SG, Ali B, Albar AA, Ahmed H, Alhasan AY. Bronchial asthma in two chemical fertilizer producing factories in eastern Saudi Arabia. PubMed. 1998;2[4]:330-335. https://pubmed.ncbi.nlm.nih.gov/9559405
178. Ali BA, Ahmed H, Ballal SG, Albar AA. Pulmonary function of workers exposed to ammonia: a study in the eastern province of Saudi Arabia. International Journal of Occupational and Environmental Health. Published online January 1, 2001. doi:10.1179/107735201800339669
179. Al‐Sarar AS, Bakr YA, Al-Erimah GS, Hussein HA, Bayoumi AE. Hematological and biochemical alterations in occupationally Pesticides-Exposed workers of Riyadh municipality, Kingdom of Saudi Arabia. Research Journal of Environmental Toxicology. 2009;3[4]:179-185. doi:10.3923/rjet.2009.179.185
180. Al-Malki AL. Serum heavy metals and hemoglobin related compounds in Saudi Arabia firefighters. Journal of Occupational Medicine and Toxicology. 2009;4[1]:18. doi:10.1186/1745-6673-4-18
181. Balkhyour MA, Goknil MK. Total Fume and Metal Concentrations during Welding in Selected Factories in Jeddah, Saudi Arabia. International Journal of Environmental Research and Public Health. 2010;7[7]:2978-2987. doi:10.3390/ijerph7072978
182. El-Helaly M, Balkhy HH, Khan W, Khawaja S. Respiratory symptoms and ventilatory function among health-care workers exposed to cleaning and disinfectant chemicals, a 2-year follow-up study. Toxicology and Industrial Health. 2016;32[12]:2002-2008. doi:10.1177/0748233715610043
183. Salama KF, Bashawri L. Biochemical and hematological changes among saudi firefighters in the eastern province. International Journal of Environmental Health Engineering. 2017;6[1]:2. doi:10.4103/ijehe.ijehe_12_15
184. Meo SA, Almutairi FJ, Alasbali MM, et al. Men’s Health in Industries: Plastic Plant Pollution and Prevalence of Pre-diabetes and Type 2 Diabetes Mellitus. American Journal of Men’s Health. 2018;12[6]:2167-2172. doi:10.1177/1557988318800203
185. Rahmani AH, Al-Hurabi AA, Joseph RJ, Babiker AY. STUDY OF WORK RELATED RESPIRATORY SYMPTOMS AMONG WELDING WORKERS. Asian Journal of Pharmaceutical and Clinical Research. 2018;11[2]:97. doi:10.22159/ajpcr.2018.v11i2.22767
186. Carrieri M, Pigini D, Martinelli A, et al. Effect of benzene exposure on the urinary biomarkers of nucleic acid oxidation in two cohorts of gasoline pump attendants. International Journal of Environmental Research and Public Health. 2019;16[1]:129. doi:10.3390/ijerph16010129
187. Shaik AP, Alsaeed AH, Faiyaz‐Ul‐Haque M, Alsaeed MA, Shaik AS. Research Article Human Serum Paraoxonase [PON-1] and hemochromatosis gene [HFE] gene polymorphisms in occupationally exposed lead workers from Saudi Arabia. Genetics and Molecular Research. 2019;18[2]. doi:10.4238/gmr18317
188. Aljohaney AA, Daali SM, Al-Juaid H, Ageel M, Sukkar S, Alhejaili F. RESPIRATORY SYMPTOMS AND PULMONARY FUNCTION TEST AMONG SALON EMPLOYEES IN JEDDAH, SAUDI ARABIA: CROSS-SECTIONAL STUDY. Chest. 2018;154[4]:791A. doi:10.1016/j.chest.2018.08.712
189. Ahmad MS. Work Related Respiratory Complications among the Worker of Qassim Cement Industry of Qassim Region, Saudi Arabia. https://www.jrmds.in/abstract/work-related-respiratory-complications-among-the-worker-of-qassim-cement-industry-of-qassim-region-saudi-arabia-44604.html
190. Sa M, Yab M, Na B, Ma A, Rf H, As A. Prevalence of Pre Diabetes and Type 2 Diabetes Mellitus among cement industry workers. Pakistan Journal of Medical Sciences. 2019;36[2]. doi:10.12669/pjms.36.2.1266
191. Meo SA, Al-Khlaiwi T, Abukhalaf AA, et al. The Nexus between Workplace Exposure for Wood, Welding, Motor Mechanic, and Oil Refinery Workers and the Prevalence of Prediabetes and Type 2 Diabetes Mellitus. International Journal of Environmental Research and Public Health. 2020;17[11]:3992. doi:10.3390/ijerph17113992
192. Ahmad I, Balkhyour MA. Occupational exposure and respiratory health of workers at small scale industries. Saudi Journal of Biological Sciences. 2020;27[3]:985-990. doi:10.1016/j.sjbs.2020.01.019
193. Barradah RK, Ahmad MS, Shaik RA, Ahmad R, Al-Mutairi A, Alghuyaythat WK. Assessment of hand or foot eczema and contact dermatitis among car mechanics. DOAJ [DOAJ: Directory of Open Access Journals]. 2021;25[10]:3737-3744. doi:10.26355/eurrev_202105_25941
194. Khathlan NA, Al‐dabbus Z, Al‐khdir N, Al‐Matar M, Al‐Nusaif S, Yami BA. Incense [bakhour] smoke exposure is associated with respiratory symptoms and impaired lung function among adults: A cross‐sectional study in Eastern Province of Saudi Arabia. Indoor Air. 2021;31[5]:1577-1582. doi:10.1111/ina.12833
195. Al-Khlaiwi T, Meo SA, Habib SS, Meo IMU, Alqhtani MS. Incense Burning Indoor Pollution: Impact on the prevalence of prediabetes and Type-2 Diabetes Mellitus. Pakistan Journal of Medical Sciences. 2022;38[7]. doi:10.12669/pjms.38.7.6189
196. Adly HM, Saleh SA. The association of increased oxidative stress and tumor biomarkers related to polyaromatic hydrocarbons exposure for different occupational workers in Makkah, Saudi Arabia. Cureus. Published online December 26, 2022. doi:10.7759/cureus.32981
197. Mergler D, Vézina N, Beauvais A. Warts among workers in poultry slaughterhouses. PubMed. 1982;8 Suppl 1:180-184. https://pubmed.ncbi.nlm.nih.gov/7100850
198. Ahmad S. The prevalence of Staphylococcus aureus colonization among healthcare workers at a specialist hospital in... Journal of Clinical and Diagnostic Research. 2010;4[3]:2438-2441. http://www.jcdr.net/back_issues.asp?issn=0973-709x&year=2010
199. Mazi W, Senok A, Assiri AM, Kazem N, Abato AT. Occupational exposure to Blood-Borne pathogens in a tertiary hospital. Asia Pacific Journal of Public Health. 2012;27[2]:NP1727-NP1732. doi:10.1177/1010539512450608
200. Balkhy HH, Miller TL, Ali S, et al. Compliance with Postexposure Screening and Treatment of Latent Tuberculosis Infection among Healthcare Workers in a Tertiary Care Hospital in Saudi Arabia. Infection Control and Hospital Epidemiology. 2014;35[2]:176-181. doi:10.1086/674855
201. Yezli S, Alotaibi BM, Al-Abdely HM, et al. Acquisition of respiratory and gastrointestinal pathogens among health care workers during the 2015 Hajj season. American Journal of Infection Control. 2019;47[9]:1071-1076. doi:10.1016/j.ajic.2019.02.033
202. Almasri M, Ahmed QA, Turkestani A, Memish ZA. Hajj abattoirs in Makkah: risk of zoonotic infections among occupational workers. Veterinary Medicine and Science. 2019;5[3]:428-434. doi:10.1002/vms3.169
