# Supplementary material for: Occupational health in the Gulf Cooperation Council (GCC): A systematic review and call for comprehensive policy development
Source: PLoS One. 2024 Dec 10;19(12):e0312251. doi: 10.1371/journal.pone.0312251 (PMC11630603; doi:10.1371/journal.pone.0312251)
Supplement: S4 Table — (DOCX) [file pone.0312251.s005.docx]

| Table S4: comprehensive table exhibiting the results of the selected studies. | | | | | | | |
| --- | --- | --- | --- | --- | --- | --- | --- |
| **#** | **Country** | **Title** | **Authors** | **Published Year** | **Journal** | **DOI** | **Reason for Exclusion** |
| 1 | Bahrain | Burden of musculoskeletal disorders in the gulf cooperation council countries,  1990-2019: Findings from the global burden of disease study 2019. | Alzahrani H; Alshehri MA; Alotaibi M; Alhowimel A; Alodaibi F; Alamam D; Zheng Y; Tyrovolas S | 2022 | Front Med (Lausanne) | 10.3389/fmed.2022.855414 | Wrong outcomes |
| 2 |  | A survey of burnout of the mental health occupational therapy staff in  the Psychiatric Hospital, Bahrain | Jahrami, H. | 2009 | British Journal of Occupational Therapy | 10.1177/030802260907201008 | Psychological outcomes |
| 3 |  | The examination of sleep quality for frontline healthcare workers during the outbreak of COVID-19 | Jahrami, H.; BaHammam, A.S.; AlGahtani, H.; Ebrahim, A.; Faris, M.A.I.; AlEid, K.; Saif, Z.; Haji, E.; Dhahi, A.; Marzooq, H.; Hubail, S.; Hasan, Z. | 2021 | Sleep and Breathing | 10.1007/s11325-020-02135-9 | Covid related study |
| 4 |  | Prevalence of stress, anxiety and depression among expatriate workers | Latif Sarwani, S.A.A.; Abdulla, K.B.; Jafar Mandeel, M.A. | 2013 | Bahrain Medical Bulletin |  | Psychological outcomes |
| 5 |  | Stress and psychological resilience among general surgery residents during COVID-19 pandemic | Aljehani, Y.M.; Othman, S.A.; Telmesani, N.K.; Alghamdi, R.A.; AlBuainain, H.M.; Alghamdi, Z.M.; Zakaria, H.M.; Alreshaid, F.T.; Busbait, S.A.; Alqarzaie, A.A.; Alharbi, T.M.; Alnajim, R.K. | 2020 | Saudi Medical Journal | 10.15537/smj.2020.12.25577 | Covid related study |
| 6 |  | The Impact of Occupational Stress and Burnout among Employees of Different Age Groups on Job Satisfaction: Evidence from Saudi Arabia and Bahrain | George, S.; Al Saffar, E.M.; Almohammedsaleh, Z. | 2021 | 2021 International Conference on Sustainable Islamic Business and Finance, SIBF 2021 | 10.1109/IEEECONF53626.2021.9686330 | Psychological outcomes |
| 7 |  | Pesticide handlers' knowledge, attitude and practice | Al-Haddad, S.A.; Al-Sayyad, A.S. | 2013 | Bahrain Medical Bulletin | 10.12816/0000508 | Wrong outcomes |
| 8 |  | OCCUPATIONAL INJURIES IN BAHRAIN | ALARRAYED, A; HAMZA, A | 1995 | OCCUPATIONAL MEDICINE-OXFORD | 10.1093/occmed/45.5.231 | Wrong outcomes |
| 9 |  | A qualitative investigation of the occupational stress in the hospitality and tourism industry in Bahrain during COVID-19 | Ayari, A | 2023 | ORGANIZATSIONNAYA PSIKOLOGIYA | 10.17323/2312-5942-2023-13-2-64-77 | Covid related study |
| 10 | Oman | Workplace bullying, occupational burnout, work-life imbalance and perceived medical errors among nurses in Oman: A cluster analysis. | Chan MF; Al Balushi AA; Al-Adawi S; Alameddine M; Al Saadoon M; Bou-Karroum K | 2022 | J Nurs Manag | 10.1111/jonm.13432 | Psychological outcomes |
| 11 |  | Burnout Syndrome Among Primary Care Physicians in Oman. | Al-Hashemi T; Al-Huseini S; Al-Alawi M; Al-Balushi N; Al-Senawi H; Al-Balushi M; Jose S; Al-Adawi S | 2019 | Oman Med J | 10.5001/omj.2019.40 | Psychological outcomes |
| 12 |  | Implemented occupational health surveillance limits the spread of SARS-CoV-2 Omicron at the workplace. | Gesto JSM; Cabanelas A; Farjun B; Dos Santos MC; Fidalgo-Neto AA; Kuriyama SN; Souza TML | 2022 | Front Med (Lausanne) | 10.3389/fmed.2022.910176 | Covid related study |
| 13 |  | Prevalence and determinants of burnout Syndrome and Depression among medical students at Sultan Qaboos University: A cross-sectional analytical study from Oman. | Al-Alawi M; Al-Sinawi H; Al-Qubtan A; Al-Lawati J; Al-Habsi A; Al-Shuraiqi M; Al-Adawi S; Panchatcharam SM | 2019 | Arch Environ Occup Health | 10.1080/19338244.2017.1400941 | Psychological outcomes |
| 14 |  | Serological, cultural and molecular evidence of Brucella melitensis infection in goats in Al Jabal Al Akhdar, Sultanate of Oman. | ElTahir Y; Al Toobi AG; Al-Marzooqi W; Mahgoub O; Jay M; Corde Y; Al Lawati H; Bose S; Al Hamrashdi A; Al Kharousi K; Al-Saqri N; Al Busaidi R; Johnson EH | 2018 | Vet Med Sci | 10.1002/vms3.103 | Out of the scope |
| 15 |  | Risk Factors Associated with COVID-19 Infected Healthcare Workers in Muscat Governorate, Oman. | Al Abri ZGH; Al Zeedi MASA; Al Lawati AA | 2021 | J Prim Care Community Health | 10.1177/2150132721995454 | Covid related study |
| 16 |  | Erysipelothrix rhusiopathiae endocarditis diagnosed by broad range 16s rRNA PCR gene sequencing. | Balkhair A; Al Lawati H; Al Riyami M; Alameddine T; Al Amin M; Al Adawi B | 2019 | IDCases | 10.1016/j.idcr.2019.e00584 | Out of the scope |
| 17 |  | Adverse Skin Reactions to Personal Protective Equipment Among Healthcare Workers in Oman During the Coronavirus Disease 2019 Pandemic. | Al Badri F; Al Ali A; Al Saidi Y; Al Bahri Z; Al Hashimi S | 2023 | Cureus | 10.7759/cureus.33223 | Covid related study |
| 18 |  | Identification of Asymptomatic Severe Acute Respiratory Syndrome Coronavirus 2 Infections Among Healthcare Workers at Sultan Qaboos University Hospital, Oman. | Al Shibli A; Al Jufaili M; Al Alawi A; Balkhair A; Al Zakwani I; Al Azri F; Al Maamari K; Ba Alawi F; Al Qayoudhi A; Al Ghafri H | 2023 | Sultan Qaboos Univ Med J | 10.18295/squmj.1.2023.008 | Covid related study |
| 19 |  | Trends of sickness certifications in primary health care in muscat, sultanate of oman. | A'Rashdy F; Al-Hosni K; Al-Rawahi A; Theodorsson T | 2015 | Oman Med J | 10.5001/omj.2015.21 | Wrong outcomes |
| 20 |  | Occupational stress, coping strategies, and the impact of culture in the Middle East: A systematic review of evidence from Oman | Sadriwala, K.F.; Malik, M. | 2021 | Organizational Stress Around the World: Research and Practice |  | Psychological outcomes |
| 21 |  | Personal safety issues related to the use of pesticides in agricultural production in the Al-Batinah region of Northern Oman | Al Zadjali, S.; Morse, S.; Chenoweth, J.; Deadman, M. | 2015 | Science of the Total Environment | 10.1016/j.scitotenv.2014.09.044 | Out of the scope |
| 22 |  | Assessment of lead, zinc, copper, nickel and chromium in total suspended particulate matter from the workplace in Al-Rusayl Industrial Estate, Oman | Yaghi, B.; Abdul-Wahab, S.A. | 2003 | Journal of Environmental Monitoring | 10.1039/b306029m | Out of the scope |
| 23 |  | Prevalence and persistence of SARS-CoV2 antibodies among healthcare workers in Oman | Al-Naamani, K.; Al-Jahdhami, I.; Al-Tamtami, W.; Al-Amri, K.; Al-Khabori, M.; Sinani, S.A.; Said, E.A.; Omer, H.; Al-Bahluli, H.; Al-Ryiami, S.; Al-Hakmani, S.; Al-Naamani, N.; Al-Jahwari, R.; Al-Hinai, M.; AlWahaibi, J. | 2021 | Journal of Infection and Public Health | 10.1016/j.jiph.2021.09.006 | Covid related study |
| 24 |  | Briefing: Cost of accidents in the construction industry of Oman | Umar, T. | 2017 | Proceedings of the Institution of Civil Engineers: Municipal Engineer | 10.1680/jmuen.16.00032 | Out of the scope |
| 25 |  | Heat stress, a hidden cause of accidents in construction | Umar, T.; Egbu, C. | 2020 | Proceedings of the Institution of Civil Engineers: Municipal Engineer | 10.1680/jmuen.18.00004 | Out of the scope |
| 26 |  | Demography of orthopaedic admissions in a secondary care hospital in oman | Chandrashekara, C.M.; George, M.A.; Al-Marboi, B.S.K. | 2013 | Brunei International Medical Journal |  | Out of the scope |
| 27 |  | Safe injection practices in a primary health care setting in Oman | Al Awaidy, S.; Bawikar, S.; Duclos, P. | 2006 | Eastern Mediterranean Health Journal |  | Wrong setting |
| 28 |  | Modeling, Investigating, and Quantification of the Hot Weather Effects on Construction Projects in Oman | Balushi, H.A.; AL-Alawi, M.; Shahri, M.A. | 2020 | Journal of Engineering Research | 10.24200/tjer.vol17iss2pp89-99 | Wrong outcomes |
| 29 |  | A Comparative Study of Occupational Safety and Health (OS&H) Regulations in United States, United Kingdom, Australia, South Africa, and Oman | Umar, T.; Umeokafor, N.; Honnur Vali, M.S.; Zia, A. | 2022 | IOP Conference Series: Earth and Environmental Science | 10.1088/1755-1315/1101/3/032016 | Out of the scope |
| 30 |  | Replantation of nine fingers in a patient: A case report | Patil, R.K.; Malhotra, G.; Venugopal, S.; Salah, E.; Ramadan, A. | 2019 | Sultan Qaboos University Medical Journal | 10.18295/squmj.2019.19.03.012 | Wrong setting |
| 31 |  | Briefing: Occupational safety and health regulations in Oman | Umar, T.; Egbu, C.; Wamuziri, S.; Honnurvali, M.S. | 2018 | Proceedings of Institution of Civil Engineers: Management, Procurement and Law | 10.1680/jmapl.18.00007 | Out of the scope |
| 32 |  | Are Indian expatriates in Sultanate of Oman under stress? | Deosthalee, P.G. | 2002 | Journal of Managerial Psychology | 10.1108/02683940210439423 | Psychological outcomes |
| 33 |  | Impacts on ambient air quality due to flaring activities in one of Oman's oilfields | Abdul-Wahab, S.; Ali, S.; Sardar, S.; Irfan, N. | 2012 | Archives of Environmental and Occupational Health | 10.1080/19338244.2011.573021 | Out of the scope |
| 34 |  | The role of supporting services in driving SARS-CoV-2 transmission within healthcare settings: A multicenter seroprevalence study | Al-Maani, A.; Al Wahaibi, A.; Al-Sooti, J.; Al Abri, B.; Al Shukri, I.; AlRisi, E.; Al Abri, L.; AlDaghari, K.; Al Subhi, M.; AlMaqbali, S.; AlBurtamani, S.; AlAbri, A.; Al Salami, A.; Al-Beloushi, I.; Al-Zadjali, N.; Alqayoudhi, A.; Al-Kindi, H.; Al Shaqsi, K.; Al-Jardani, A.; Al-Abri, S. | 2021 | International Journal of Infectious Diseases | 10.1016/j.ijid.2021.04.071 | Covid related study |
| 35 |  | Middle East respiratory syndrome coronavirus in the last two years: Health care workers still at risk | Al-Tawfiq, J.A.; Memish, Z.A. | 2019 | American Journal of Infection Control | 10.1016/j.ajic.2019.04.007 | Covid related study |
| 36 |  | Total suspended dust and heavy metal levels emitted from a workplace compared with nearby residential houses | Abdul-Wahab, S.A.; Yaghi, B. | 2004 | Atmospheric Environment | 10.1016/j.atmosenv.2003.10.017 | Out of the scope |
| 37 |  | Burnout and work stress among disability centers staff in Oman | Mohamed, A.H.H. | 2015 | International Journal of Special Education |  | Psychological outcomes |
| 38 |  | Construction SMEs safety challenges in water sector in Oman | Al Mawli, B.; Al Alawi, M.; Elazouni, A.; Al-Mamun, A. | 2021 | Safety Science | 10.1016/j.ssci.2020.105156 | Wrong outcomes |
| 39 | Qatar | Occupational Safety and Work-Related Injury Control Efforts in Qatar: Lessons Learned from a Rapidly Developing Economy. | Consunji RJ; Mehmood A; Hirani N; El-Menyar A; Abeid A; Hyder AA; Al-Thani H; Peralta R | 2020 | Int J Environ Res Public Health | 10.3390/ijerph17186906 | Out of the scope |
| 40 |  | Occupational Exposure to Dromedaries and Risk for MERS-CoV Infection, Qatar, 2013-2014. | Reusken CB; Farag EA; Haagmans BL; Mohran KA; Godeke GJ 5th; Raj S; Alhajri F; Al-Marri SA; Al-Romaihi HE; Al-Thani M; Bosch BJ; van der Eijk AA; El-Sayed AM; Ibrahim AK; Al-Molawi N; M√ºller MA; Pasha SK; Drosten C; AlHajri MM; Koopmans MP | 2015 | Emerg Infect Dis | 10.3201/eid2108.150481 | Covid related study |
| 41 |  | Work related injuries in Qatar: a framework for prevention and control. | Mehmood A; Maung Z; Consunji RJ; El-Menyar A; Peralta R; Al-Thani H; Hyder AA | 2018 | J Occup Med Toxicol | 10.1186/s12995-018-0211-z | Out of the scope |
| 42 |  | Occupational Heat Stress: Multi-Country Observations and Interventions. | Ioannou LG; Mantzios K; Tsoutsoubi L; Nintou E; Vliora M; Gkiata P; Dallas CN; Gkikas G; Agaliotis G; Sfakianakis K; Kapnia AK; Testa DJ; Amorim T; Dinas PC; Mayor TS; Gao C; Nybo L; Flouris AD | 2021 | Int J Environ Res Public Health | 10.3390/ijerph18126303 | Out of the scope |
| 43 |  | ICOH Statement on Protecting the Occupational Safety and Health of Migrant Workers. |  | 2022 | Saf Health Work | 10.1016/j.shaw.2022.06.004 | Out of the scope |
| 44 |  | Job satisfaction and stress among healthcare workers in public hospitals in Qatar. | Yehya A; Sankaranarayanan A; Alkhal A; Alnoimi H; Almeer N; Khan A; Ghuloum S | 2020 | Arch Environ Occup Health | 10.1080/19338244.2018.1531817 | Psychological outcomes |
| 45 |  | Health Status and Working Condition of Migrant Workers: Major Public Health Problems. | Bener A | 2017 | Int J Prev Med | 10.4103/ijpvm.IJPVM_396_16 | Out of the scope |
| 46 |  | Epidemiology of workplace-related fall from height and cost of trauma care in Qatar. | Tuma MA; Acerra JR; El-Menyar A; Al-Thani H; Al-Hassani A; Recicar JF; Al Yazeedi W; Maull KI | 2013 | Int J Crit Illn Inj Sci | 10.4103/2229-5151.109408 | Out of the scope |
| 47 |  | SARS-CoV-2 infection in mortuary and cemetery workers. | Alishaq M; Jeremijenko A; Nafady-Hego H; Al Ajmi JA; Elgendy M; Fadel RAA; Thomas AG; Alahmed MAA; Ammar A; Bensaad M; Al-Barghouthi B; Coyle P; Elgendy H; Abou-Samra AB; Butt AA | 2021 | Int J Infect Dis | 10.1016/j.ijid.2021.03.012 | Covid related study |
| 48 |  | Direct Healthcare Costs of Moderate and Severe Work-Related Injuries: Estimates from the National Trauma Center of Qatar. | Consunji RJ; Mekkodathil A; El-Menyar A; Mehmood A; Sathian B; Hyder AA; Hirani N; Abeid A; Al-Thani H; Peralta R | 2022 | Int J Environ Res Public Health | 10.3390/ijerph19031609 | Out of the scope |
| 49 |  | COVID-19 infection across workplace settings in Qatar: a comparison of COVID-19 positivity rates of screened workers from March 1st until July 31st, 2020. | Al-Kuwari MG; Al-Nuaimi AA; Abdulmajeed J; Semaan S; Al-Romaihi HE; Kandy MC; Swamy S | 2021 | J Occup Med Toxicol | 10.1186/s12995-021-00311-5 | Covid related study |
| 50 |  | Workplace-related traumatic injuries: insights from a rapidly developing Middle Eastern country. | Al-Thani H; El-Menyar A; Abdelrahman H; Zarour A; Consunji R; Peralta R; Asim M; El-Hennawy H; Parchani A; Latifi R | 2014 | J Environ Public Health | 10.1155/2014/430832 | Out of the scope |
| 51 |  | Occupational Prevention of COVID-19 Among Healthcare Workers in Primary Healthcare Settings: Compliance and Perceived Effectiveness of Personal Protective Equipment. | Abed Alah MTT; Abdeen S; Selim N; Tayar E; Bougmiza I | 2022 | J Patient Saf | 10.1097/PTS.0000000000001004 | Covid related study |
| 52 |  | Characteristics and predictors of burnout among healthcare professionals: a cross-sectional study in two tertiary hospitals. | El-Menyar A; Ibrahim WH; El Ansari W; Gomaa M; Sathian B; Hssain AA; Wahlen B; Nabir S; Al-Thani H | 2021 | Postgrad Med J | 10.1136/postgradmedj-2020-137547 | Psychological outcomes |
| 53 |  | Epidemiology Characteristics of COVID-19 Infection Amongst Primary Health Care Workers in Qatar: March-October 2020. | Al-Kuwari MG; AbdulMalik MA; Al-Nuaimi AA; Abdulmajeed J; Al-Romaihi HE; Semaan S; Kandy M | 2021 | Front Public Health | 10.3389/fpubh.2021.679254 | Covid related study |
| 54 |  | Thinner burns in Qatar | Al-Tamimi, N.; Tawfiq, F.A.; Al-Ghoul, A. | 2008 | Journal of Emergency Medicine, Trauma and Acute Care |  | Full text unavailable |
| 55 |  | Trauma caused by falling objects at construction sites | Atique, S.; Zarour, A.; Siddiqui, T.; El-Menyar, A.; Maull, K.; Al Thani, H.; Latifi, R. | 2012 | Journal of Trauma and Acute Care Surgery | 10.1097/TA.0b013e31825472d7 | Out of the scope |
| 56 |  | Middle East respiratory syndrome coronavirus in the last two years: Health care workers still at risk | Al-Tawfiq, J.A.; Memish, Z.A. | 2019 | American Journal of Infection Control | 10.1016/j.ajic.2019.04.007 | Covid related study |
| 57 |  | Sickness absenteeism among primary health care workers in Qatar before and during the COVID-19 pandemic | Al-Nuaimi, A.A.; Abdeen, S.; Abed Alah, M.; AlHajri, S.; Semaan, S.; Al-Kuwari, M.G. | 2023 | Journal of Occupational Medicine and Toxicology | 10.1186/s12995-023-00369-3 | Covid related study |
| 58 |  | Performance, productivity, and safety & health among employee of oil & gas company in Qatar | Syarnubi, A.; Sembiring, R.A.; Siswaya, T.; Zuraida, R. | 2018 | IOP Conference Series: Earth and Environmental Science | 10.1088/1755-1315/195/1/012075 | Wrong outcomes |
| 59 |  | Tuberculosis among garment workers in an Arabian developing country: State of Qatar | Al-Khal, A.L.; Bener, A.; Enarson, D.A. | 2005 | Archives of Environmental and Occupational Health | 10.3200/AEOH.60.6.295-298 | Out of the scope |
| 60 |  | Safety in the oil and gas industries in Qatar | Mannan, M.S.; Olewski, T.; Waldram, S. | 2009 | Institution of Chemical Engineers Symposium Series |  | Wrong outcomes |
| 61 |  | Patterns and outcomes of traumatic neck injuries: A population-based observational study | Al-Thani, H.; El-Menyar, A.; Mathew, S.; Khawar, M.; Asim, M.; Abdelrahman, H.; Peralta, R.; Parchani, A.; Zarour, A. | 2015 | Journal of Emergencies, Trauma and Shock | 10.4103/0974-2700.160723 | Out of the scope |
| 62 |  | Safety of healthcare workers undertaking transport and retrieval of patients on extracorporeal membrane oxygenation during the peak of COVID-19 pandemic in the State of Qatar | Shehatta, A.L.; Racela, B.; Howard, I.; Alinier, G.; Jaouni, H.; Hassan, I. | 2021 | Journal of Emergency Medicine, Trauma and Acute Care | 10.5339/jemtac.2021.qhc.12 | Covid related study |
| 63 |  | Patterns of infection with intestinal parasites in Qatar among food handlers and housemaids from different geographical regions of origin | Abu-Madi, M.A.; Behnke, J.M.; Ismail, A. | 2008 | Acta Tropica | 10.1016/j.actatropica.2008.03.010 | Out of the scope |
| 64 | Kuwait | Occupational Stressors and Safety Behaviour among Oil and Gas Workers in Kuwait: The Mediating Role of Mental Health and Fatigue. | Alroomi AS; Mohamed S | 2021 | Int J Environ Res Public Health | 10.3390/ijerph182111700 | Psychological outcomes |
| 65 |  | Assessing occupational stress, strain, and coping for North American teachers in Kuwait. | Alkhadher O; Al-Naser H | 2006 | Psychol Rep | 10.2466/PR0.99.3.681-690 | Psychological outcomes |
| 66 |  | The Prevalence of Burnout and Its Associated Factors Among Surgical Specialists in Kuwait Ministry of Health Hospitals. | Akl A; Mohiyaldeen I; Alshatti R; Alenezi O; Dougherty R; Al-Raihan A; Alotaibi S; Tadros N; Longenecker JC | 2022 | Front Public Health | 10.3389/fpubh.2022.679834 | Psychological outcomes |
| 67 |  | Effectiveness of BNT162b2 and ChAdOx1 Vaccines against Symptomatic COVID-19 among Healthcare Workers in Kuwait: A Retrospective Cohort Study. | Alali WQ; Ali LA; AlSeaidan M; Al-Rashidi M | 2021 | Healthcare (Basel) | 10.3390/healthcare9121692 | Covid related study |
| 68 |  | Physical isolation and safety behaviour among oil and gas workers in Kuwait: The mediating role of mental health | Alroomi, A.S.; Mohamed, S. | 2022 | Journal of Loss Prevention in the Process Industries | 10.1016/j.jlp.2021.104692 | Psychological outcomes |
| 69 |  | THE IMPACT OF QUALITY OF WORK-LIFE ON REDUCING OCCUPATIONAL BURNOUT AMONG WORKERS | Alabduljader, S.A. | 2022 | Polish Journal of Management Studies | 10.17512/pjms.2022.26.2.02 | Psychological outcomes |
| 70 |  | Does fatigue mediate the relation between physical isolation and safety behaviour among isolated oil and gas workers? | Alroomi, A.S.; Mohamed, S. | 2022 | Safety Science | 10.1016/j.ssci.2021.105639 | Out of the scope |
| 71 |  | Gender differences in quality of life, physical activity, and risk of hypertension among sedentary occupation workers | Badr, H.E.; Rao, S.; Manee, F. | 2021 | Quality of Life Research | 10.1007/s11136-020-02741-w | Out of the scope |
| 72 |  | Pesticide risk behaviors and factors influencing pesticide use among farmers in Kuwait | Jallow, M.F.A.; Awadh, D.G.; Albaho, M.S.; Devi, V.Y.; Thomas, B.M. | 2017 | Science of the Total Environment | 10.1016/j.scitotenv.2016.09.085 | Out of the scope |
| 73 |  | Fatalities and injuries in the Kuwaiti construction industry | Kartam, N.A.; Bouz, R.G. | 1998 | Accident Analysis and Prevention | 10.1016/S0001-4575(98)00033-5 | Wrong outcomes |
| 74 |  | Epidemiology of mercurialism in Kuwait | Hassan, R.Z.; Rahim, M.Y.A. | 1978 | Journal of the Kuwait Medical Association |  | Full-text unavailable |
| 75 |  | Lack of transmission of hepatitis C virus following needlestick accidents | Hasan, F.; Askar, H.; Al Khalidi, J.; Al Shamali, M.; Al Kalaoui, M.; Al Nakib, B. | 1999 | Hepato-Gastroenterology |  | Full-text unavailable |
| 76 |  | Preliminary assessment of indoor industrial noise pollution in kuwait | Khuraibet, A.M.; Al-Attar, F. | 2000 | Environmentalist | 10.1023/A:1006717631649 | Wrong outcomes |
| 77 |  | Prevalence of posttraumatic stress disorder among Kuwaiti firefighters. | al-Naser, F.; Everly Jr., G.S. | 1999 | International journal of emergency mental health |  | Psychological outcomes |
| 78 | United Arab Emirates | Estimate of Occupational Exposure to Carcinogens among Migrant Workers in the United Arab Emirates: A Cross-Sectional Study. | Elbarazi I; El-Zaemey S; Saddik B; √Åd√°m B; El Sadig M; Abdullahi AS; Fritschi L; Sheek-Hussein M | 2022 | Int J Environ Res Public Health | 10.3390/ijerph192013012 | Out of the scope |
| 79 |  | SARS-CoV-2 and COVID-19 Research Trend during the First Two Years of the Pandemic in the United Arab Emirates: A PRISMA-Compliant Bibliometric Analysis. | Al-Omari B; Ahmad T; Al-Rifai RH | 2022 | Int J Environ Res Public Health | 10.3390/ijerph19137753 | Covid related study |
| 80 |  | Occupational Health of Frontline Healthcare Workers in the United Arab Emirates during the COVID-19 Pandemic: A Snapshot of Summer 2020. | Ajab S; √Ådam B; Al Hammadi M; Al Bastaki N; Al Junaibi M; Al Zubaidi A; Hegazi M; Grivna M; Kady S; Koornneef E; Neves R; Uva AS; Sheek-Hussein M; Loney T; Serranheira F; Paulo MS | 2021 | Int J Environ Res Public Health | 10.3390/ijerph182111410 | Covid related study |
| 81 |  | Estimated burden of disease attributable to selected occupational exposures in the United Arab Emirates. | Folley TJ; Nylander-French LA; Joubert DM; Gibson JM | 2012 | Am J Ind Med | 10.1002/ajim.22043 | Out of the scope |
| 82 |  | Epidemiology of spinal injuries in the United Arab Emirates. | Grivna M; Eid HO; Abu-Zidan FM | 2015 | World J Emerg Surg | 10.1186/s13017-015-0015-8 | Out of the scope |
| 83 |  | Epidemiology of isolated hand injuries in the United Arab Emirates. | Grivna M; Eid HO; Abu-Zidan FM | 2016 | World J Orthop | 10.5312/wjo.v7.i9.570 | Out of the scope |
| 84 |  | An outbreak of Crimean-Congo hemorrhagic fever in the United Arab Emirates, 1994-1995. | Khan AS; Maupin GO; Rollin PE; Noor AM; Shurie HH; Shalabi AG; Wasef S; Haddad YM; Sadek R; Ijaz K; Peters CJ; Ksiazek TG | 1997 | Am J Trop Med Hyg | 10.4269/ajtmh.1997.57.519 | Out of the scope |
| 85 |  | Occupational noise-induced hearing loss among dental professionals. | Al-Rawi NH; Al Nuaimi AS; Sadiqi A; Azaiah E; Ezzeddine D; Ghunaim Q; Abbas Z | 2019 | Quintessence Int | 10.3290/j.qi.a41907 | Out of the scope |
| 86 |  | Noise levels, noise annoyance, and hearing-related problems in a dental college. | Ahmed HO; Ali WJ | 2017 | Arch Environ Occup Health | 10.1080/19338244.2016.1179169 | Wrong population |
| 87 |  | Performance of the Hamilton Depression Rating Scale in depressed patients in the United Arab Emirates. | Hamdi E; Amin Y; Abou-Saleh MT | 1997 | Acta Psychiatr Scand | 10.1111/j.1600-0447.1997.tb09942.x | Psychological outcomes |
| 88 |  | Personal protective equipment-related dermatoses in COVID-19 frontline health workers. A lesson learned from 1-year single center in the UAE. | Al Zaabi A; Abdelhadi S; Ruszczak Z | 2022 | Dermatol Ther | 10.1111/dth.15624 | Covid related study |
| 89 |  | Early retirement intentions among Abu Dhabi Police: investigating the role of psychosocial work factors and sickness absenteeism. | Alkaabi FA; Maghelal PK | 2023 | BMC Public Health | 10.1186/s12889-023-16129-1 | Psychological outcomes |
| 90 |  | Prevalence of Helicobacter pylori infection among low socio-economic workers. | Bener A; Uduman SA; Ameen A; Alwash R; Pasha MA; Usmani MA; AI-Naili SR; Amiri KM | 2002 | J Commun Dis |  | Out of the scope |
| 91 |  | Preliminary Study: Environmental Assessment of Perchloroethylene in Dry-Cleaning Facilities in the UAE | Habib, S.; Ahmed, H.O.; Al-Muhairi, N.; Ziad, R. | 2018 | Journal of Environmental and Public Health | 10.1155/2018/1732906 | Out of the scope |
| 92 |  | The COVID-19 pandemic reduced the trauma incidence and modified its pattern in Al-Ain City, United Arab Emirates | Alao, D.O.; Cevik, A.A.; Yasin, Y.J.; Jaiganesh, T.; Abu-Zidan, F. | 2022 | European Journal of Trauma and Emergency Surgery | 10.1007/s00068-022-01897-z | Covid related study |
| 93 |  | Epidemiology of eye injuries in a high-income developing country: An observational study | AlMahmoud, T.; Al Hadhrami, S.M.; Elhanan, M.; Alshamsi, H.N.; Abu-Zidan, F.M. | 2019 | Medicine | 10.1097/MD.0000000000016083 | Out of the scope |
| 94 |  | Psychological Stress of Teachers of People with Learning Difficulties and its Relation to Some Variables: A Field Study in the United Arab Emirates | Rabou, I.A.; Al Salihi, N.R.; Alqawasmi, A.A.; Al-Qatawneh, S.; Alghazo, E.M. | 2022 | Eurasian Journal of Applied Linguistics | 10.32601/ejal.803023 | Psychological outcomes |
| 95 |  | Risk factors for MERS-CoV seropositivity among animal market and slaughterhouse workers, Abu Dhabi, United Arab Emirates, 2014‚Äì2017 | Khudhair, A.; Killerby, M.E.; Al Mulla, M.; Elkheir, K.A.; Ternanni, W.; Bandar, Z.; Weber, S.; Khoury, M.; Donnelly, G.; Al Muhairi, S.; Khalafalla, A.I.; Trivedi, S.; Tamin, A.; Thornburg, N.J.; Watson, J.T.; Gerber, S.I.; Al Hosani, F.; Hall, A.J. | 2019 | Emerging Infectious Diseases | 10.3201/eid2505.181728 | Covid related study |
| 96 |  | Middle East respiratory syndrome coronavirus in the last two years: Health care workers still at risk | Al-Tawfiq, J.A.; Memish, Z.A. | 2019 | American Journal of Infection Control | 10.1016/j.ajic.2019.04.007 | Covid related study |
| 97 |  | Are welders more at risk of respiratory infections? Findings from a cross-sectional survey and analysis of medical records in shipyard workers: The WELSHIP project | Marongiu, A.; Hasan, O.; Ali, A.; Bakhsh, S.; George, B.; Irfan, N.; Minelli, C.; Canova, C.; Schofield, S.; De Matteis, S.; Cullinan, P. | 2016 | Thorax | 10.1136/thoraxjnl-2015-207912 | Location of the study is missing |
| 98 |  | Impact of Age and Sex on COVID-19 Severity Assessed From Radiologic and Clinical Findings | Statsenko, Y.; Al Zahmi, F.; Habuza, T.; Almansoori, T.M.; Smetanina, D.; Simiyu, G.L.; Neidl-Van Gorkom, K.; Ljubisavljevic, M.; Awawdeh, R.; Elshekhali, H.; Lee, M.; Salamin, N.; Sajid, R.; Kiran, D.; Nihalani, S.; Loney, T.; Bedson, A.; Dehdashtian, A.; Al Koteesh, J. | 2022 | Frontiers in Cellular and Infection Microbiology | 10.3389/fcimb.2021.777070 | Covid related study |
| 99 |  | Epidemiology of asymptomatic and symptomatic Coronavirus Disease 2019 confirmed cases in the Emirate of Abu Dhabi, United Arab Emirates: Observational study | Hosani, F.A.; Aden, B.; Memari, S.A.; Mazrouei, S.A.; Ajab, S.; Abid, M.; Alsuwaidi, A.R.; Grivna, M.; Paulo, M.S.; Sheek-Hussein, M. | 2021 | Medicine (United States) | 10.1097/MD.0000000000025219 | Covid related study |
| 100 |  | Self-Report Assessment of Nurses‚Äô Risk for Infection After Exposure to Patients With Coronavirus Disease (COVID-19) in the United Arab Emirates | Bani-Issa, W.A.; Al Nusair, H.; Altamimi, A.; Hatahet, S.; Deyab, F.; Fakhry, R.; Saqan, R.; Ahmad, S.; Almazem, F. | 2021 | Journal of Nursing Scholarship | 10.1111/jnu.12625 | Covid related study |
| 101 |  | Confirmatory factor analysis of work-related accidents in UAE | Al Zarooni, M.; Awad, M.; Alzaatreh, A. | 2022 | Safety Science | 10.1016/j.ssci.2022.105813 | Out of the scope |
| 102 |  | Evaluation of scattered radiation dose received by medical staff during uterine artery embolization in the operating room | Chiang, H.-W.; Chiang, H.-J.; Li, J.-H.; Tsang, L.L.-C. | 2020 | Technology and Health Care | 10.3233/THC-209002 | Out of the scope |
| 103 |  | Pesticide exposures, cholinesterase levels and symptoms among farmers | Almehdi, A.M.; Bener, A.; Dogan, M.; Pasha, M.A.H.; Usmani, M.A. | 2000 | Environmental Epidemiology and Toxicology |  | No full text |
| 104 |  | Preliminary study: Formaldehyde exposure in laboratories of Sharjah university in UAE | Ahmed, H.O. | 2011 | Indian Journal of Occupational and Environmental Medicine | 10.4103/0019-5278.82997 | Out of the scope |
| 105 |  | Hypertension among workers occupationally exposed to hydrocarbons and organic solvents | Bener, A.; Gomes, J.; Hamouda, M.F.B. | 1996 | Journal of Environmental Science and Health - Part A Toxic/Hazardous Substances and Environmental Engineering | 10.1080/10934529609376358 | Psychological outcomes |
| 106 |  | Use of the Haddon matrix as a tool for assessing risk factors for sharps injury in emergency departments in the United Arab Emirates | Ganczak, M.; Barss, P.; Al-Marashda, A.; Al-Marzouqi, A.; Al-Kuwaiti, N. | 2007 | Infection Control and Hospital Epidemiology | 10.1086/518317 | Out of the scope |
| 107 |  | Silicosis in a housewife | Safa, W.F.; Machada, J.L. | 2003 | Saudi Medical Journal |  | Out of the scope |
| 108 | Kingdom of Saudi Arabia | Prevalence of Bruxism and Associated Occupational Stress in Saudi Arabian ‚Ä®Fighter Pilots. | Al-Khalifa KS | 2022 | Oman Med J | 10.5001/omj.2022.47 | Psychological outcomes |
| 109 |  | Occupational Fatigue and Associated Factors among Saudi Nurses Working 8-Hour Shifts at Public Hospitals. | Alsayed SA; Abou Hashish EA; Alshammari F | 2022 | SAGE Open Nurs | 10.1177/23779608221078158 | Psychological outcomes |
| 110 |  | Occupational Stress and Stress Busters Used Among Saudi Dental Practitioners During the COVID-19 Pandemic Outbreak. | Aldhuwayhi S; Shaikh SA; Mallineni SK; Kumari VV; Thakare AA; Ahmed Khan AR; Mustafa MZ; Manva MZ | 2022 | Disaster Med Public Health Prep | 10.1017/dmp.2021.215 | Psychological outcomes |
| 111 |  | OCCUPATIONAL DOSE ASSESSMENT FOR NUCLEAR MEDICINE AND RADIOTHERAPY TECHNOLOGISTS IN SAUDI ARABIA. | Alashban Y; Shubayr N | 2021 | Radiat Prot Dosimetry | 10.1093/rpd/ncab112 | Out of the scope |
| 112 |  | Psychosocial and occupational factors associated with low back pain among¬†nurses in Saudi Arabia. | Jradi H; Alanazi H; Mohammad Y | 2020 | J Occup Health | 10.1002/1348-9585.12126 | Psychological outcomes |
| 113 |  | Assessment of Occupational Stress Among Oral and Maxillofacial Surgeons and Residents in Saudi Arabia: A Cross-Sectional Study. | Alkindi M; Alghamdi O; Alnofaie H; AlHammad Z; Badwelan M; Albarakati S | 2020 | Adv Med Educ Pract | 10.2147/AMEP.S268430 | Psychological outcomes |
| 114 |  | Occupational discomfort and injuries among automotive technicians in Jeddah, Saudi Arabia: A cross-sectional study. | Hafez KA | 2022 | Work | 10.3233/WOR-210522 | Out of the scope |
| 115 |  | Experiences, Perceptions, and Coping Patterns of Emergency Department Nurses with Occupational Stressors in Saudi Arabian Hospitals: Mixed-Method Study. | Alruwaili MM; Abuadas FH; Maude P; Ross A | 2022 | Healthcare (Basel) | 10.3390/healthcare10081504 | Psychological outcomes |
| 116 |  | The prevalence and the most significant sources of occupational burnout syndrome amongst anesthetic technicians in Saudi Arabia: A cross-sectional survey. | Almodibeg BA; Smith HL | 2021 | Saudi J Anaesth | 10.4103/sja.sja_1220_20 | Psychological outcomes |
| 117 |  | Assessment of job satisfaction, lifestyle behaviors, and occupational burnout symptoms during the COVID-19 pandemic among radiologic technologists in Saudi Arabia. | Shubayr N; Faraj H; Hurbush M; Khormi M; Alyami A; Majrashi N; Alomairy N | 2022 | Radiography (Lond) | 10.1016/j.radi.2022.07.015 | Covid related study |
| 118 |  | Prevalence and Risk Factors of Occupational Health Hazards among Health Care Workers of Northern Saudi Arabia: A Multicenter Study. | Thirunavukkarasu A; Alrawaili KAH; Al-Hazmi AH; Dar UF; ALruwaili B; Mallick A; Wani FA; Alsirhani AIE | 2021 | Int J Environ Res Public Health | 10.3390/ijerph182111489 | Out of the scope |
| 119 |  | Occupational-Related Contact Dermatitis: Prevalence and Risk Factors Among Healthcare Workers in the Al'Qassim Region, Saudi Arabia During the COVID-19 Pandemic. | Alluhayyan OB; Alshahri BK; Farhat AM; Alsugair S; Siddiqui JJ; Alghabawy K; AlQefari GB; Alolayan WO; Abu Hashem IA | 2020 | Cureus | 10.7759/cureus.10975 | Covid related study |
| 120 |  | Sleep and Mental Health among Paramedics from Australia and Saudi Arabia: A Comparison Study. | Khan WAA; Conduit R; Kennedy GA; Abdullah Alslamah A; Ahmad Alsuwayeh M; Jackson ML | 2020 | Clocks Sleep | 10.3390/clockssleep2020019 | Psychological outcomes |
| 121 |  | A cross-sectional survey to explore the prevalence and causes of occupational burnout syndrome among perioperative nurses in Saudi Arabia. | Almodibeg BA; Smith H | 2021 | Nurs Open | 10.1002/nop2.637 | Psychological outcomes |
| 122 |  | Occupational Stress among Orthodontists in Saudi Arabia. | Alqahtani ND; Aljajji S; Alshalan N; Aljabaa A; Aldosari M; Albarakati S | 2020 | J Int Soc Prev Community Dent | 10.4103/jispcd.JISPCD_376_19 | Psychological outcomes |
| 123 |  | Effect of Occupational Stress on Pharmacists' Job Satisfaction in Saudi Arabia. | Aldaiji L; Al-Jedai A; Alamri A; Alshehri AM; Alqazlan N; Almogbel Y | 2022 | Healthcare (Basel) | 10.3390/healthcare10081441 | Psychological outcomes |
| 124 |  | Occupational accidents and injuries in construction industry in Jeddah city. | Abukhashabah E; Summan A; Balkhyour M | 2020 | Saudi J Biol Sci | 10.1016/j.sjbs.2020.06.033 | Out of the scope |
| 125 |  | Depressed, anxious, and stressed: What have healthcare workers on the frontlines in Egypt and Saudi Arabia experienced during the COVID-19 pandemic? | Arafa A; Mohammed Z; Mahmoud O; Elshazley M; Ewis A | 2021 | J Affect Disord | 10.1016/j.jad.2020.09.080 | Covid related study |
| 126 |  | The Effect of Occupational Stress on the Quality of Life of Pharmacists in Saudi Arabia. | Almogbel Y | 2021 | Risk Manag Healthc Policy | 10.2147/RMHP.S281317 | Psychological outcomes |
| 127 |  | Mental Well-Being and Self-Efficacy of Healthcare Workers in Saudi Arabia During the COVID-19 Pandemic. | Abo-Ali EA; Al-Rubaki S; Lubbad S; Nchoukati M; Alqahtani R; Albraim S; Ghareeb WA; Al-Haffashi B; Alghamdi F; Zaytoun S | 2021 | Risk Manag Healthc Policy | 10.2147/RMHP.S320421 | Covid related study |
| 128 |  | Occupational stress in healthcare workers at a university hospital, Jeddah, Saudi Arabia. | AlMuammar SA; Shahadah DM; Shahadah AO | 2022 | J Family Community Med | 10.4103/jfcm.jfcm_157_22 | Psychological outcomes |
| 129 |  | Are dentists more prone to metabolic syndrome and occupational stress? | Alshiddi IF; Habib SR; Sattar K; Alshahrani A; Almufleh RS; Basuhail S; Andejani A | 2021 | Work | 10.3233/WOR-205134 | Psychological outcomes |
| 130 |  | Association between role conflict and ambiguity and stress among nurses in primary health care centers in Saudi Arabia during the coronavirus disease 2019 pandemic: A cross-sectional study. | Alyahya SA; Al-Mansour KA; Alkohaiz MA; Almalki MA | 2021 | Medicine (Baltimore) | 10.1097/MD.0000000000027294 | Psychological outcomes |
| 131 |  | Assessment of the Association Between Use of WhatsApp for Work-Related Purposes and Levels of Depression, Anxiety, and Stress Among Healthcare Workers from Jazan, Saudi Arabia. | Ryani MA; Alshahrani A; Khmees RA; Gosadi IM | 2023 | Psychol Res Behav Manag | 10.2147/PRBM.S402720 | Psychological outcomes |
| 132 |  | Occupational risk of hepatitis B and C infections in Saudi medical staff. | al-Sohaibani MO; al-Sheikh EH; al-Ballal SJ; Mirghani MA; Ramia S | 1995 | J Hosp Infect | 10.1016/0195-6701(95)90169-8 | Out of the scope |
| 133 |  | Prevalence and Determinants of Work-Related Injuries Among Healthcare Workers in Jeddah, Saudi Arabia. | Alameer DS; Noor Elahi IR | 2023 | Cureus | 10.7759/cureus.36679 | Out of the scope |
| 134 |  | Injury and infection in dental clinics: Risk factors and prevention. | Al-Zoughool M; Al-Shehri Z | 2018 | Toxicol Ind Health | 10.1177/0748233718769553 | Wrong patient population |
| 135 |  | Burnout and its correlates in Saudi family medicine residents: An observational study from Aseer, Saudi Arabia. | Al-Ghamdi MA; Nahar S; Siddiqui AF; Al-Saleem SA | 2021 | J Family Med Prim Care | 10.4103/jfmpc.jfmpc_2146_20 | Psychological outcomes |
| 136 |  | Verbal and Physical Abuse Against Nurses Working in Hospitals and Health Centers in Buraidah, Saudi Arabia. | Sayed F; Alrasheeday AM; Alshammari B; Alonazi A; Alharbi A; Almotairi NA; Rabbani U | 2022 | Cureus | 10.7759/cureus.31792 | Psychological outcomes |
| 137 |  | Violence against health workers in Family Medicine Centers. | Al-Turki N; Afify AA; AlAteeq M | 2016 | J Multidiscip Healthc | 10.2147/JMDH.S105407 | Out of the scope |
| 138 |  | Investigation of the Radiographic Imaging Volume and Occupational Dose of Radiologic Technologists before and during the COVID-19 Pandemic. | Shubayr N | 2023 | Health Phys | 10.1097/HP.0000000000001728 | Covid related study |
| 139 |  | Work-Related Stressors among the Healthcare Professionals in the Fever Clinic Centers for Individuals with Symptoms of COVID-19. | Alyahya S; AboGazalah F | 2021 | Healthcare (Basel) | 10.3390/healthcare9050548 | Covid related study |
| 140 |  | INVESTIGATIONS OF OCCUPATIONAL RADIATION DOSE INCIDENTS RELATED TO MEDICAL PRACTICES IN SAUDI ARABIA. | Shubayr N; Alashban Y | 2021 | Radiat Prot Dosimetry | 10.1093/rpd/ncab109 | Out of the scope |
| 141 |  | Impact of emotional intelligence on work performance: The mediating role of occupational stress among nurses. | Alsufyani AM; Aboshaiqah AE; Alshehri FA; Alsufyani YM | 2022 | J Nurs Scholarsh | 10.1111/jnu.12790 | Psychological outcomes |
| 142 |  | Occupational Violence and Staff Safety in Health-Care: A Cross-Sectional Study in a Large Public Hospital. | Al-Shaban ZR; Al-Otaibi ST; Alqahtani HA | 2021 | Risk Manag Healthc Policy | 10.2147/RMHP.S305217 | Psychological outcomes |
| 143 |  | Stress and turnover intention among healthcare workers in Saudi Arabia during the time of COVID-19: Can social support play a role? | Al-Mansour K | 2021 | PLoS One | 10.1371/journal.pone.0258101 | Covid related study |
| 144 |  | Prevalence and Associated Factors of Burnout Among Saudi Resident Doctors: A Multicenter Cross-Sectional Study. | Karim Alenezi N; Hamad Alyami A; Omar Alrehaili B; Adnan Arruhaily A; Kareem Alenazi N; Abdo Radman Al-Dubai S | 2022 | Alpha Psychiatry | 10.5152/alphapsychiatry.2022.21361 | Psychological outcomes |
| 145 |  | Prevalence and Predictors of Musculoskeletal Pain Among Undergraduate Students at a Dental School in Saudi Arabia. | Felemban RA; Sofi RA; Alhebshi SA; Alharbi SG; Farsi NJ; Abduljabbar FH; Farsi JMA | 2021 | Clin Cosmet Investig Dent | 10.2147/CCIDE.S292970 | Wrong patient population |
| 146 |  | Workplace violence towards emergency nurses: A cross-sectional multicenter study. | Alsharari AF; Abu-Snieneh HM; Abuadas FH; Elsabagh NE; Althobaity A; Alshammari FF; Alshmemri MS; Aroury AM; Alkhadam AQ; Alatawi SS | 2022 | Australas Emerg Care | 10.1016/j.auec.2021.01.004 | Psychological outcomes |
| 147 |  | Magnitude and risk factors for burnout among primary health care physicians in Asir Province, Saudi Arabia. | Al-Sareai NS; Al-Khaldi YM; Mostafa OA; Abdel-Fattah MM | 2013 | East Mediterr Health J |  | Psychological outcomes |
| 148 |  | Work-Related Musculoskeletal Symptoms in Otorhinolaryngology-Head and Neck Surgery Residents. | Dahmash AB; Alkholaiwi F; Alahmari A; Shadid AM; Alharbi AM; Al Hussain O | 2020 | Sultan Qaboos Univ Med J | 10.18295/squmj.2020.20.02.011 | Wrong patient population |
| 149 |  | Prevalence of and factors associated with burnout among health care professionals in Arab countries: a systematic review. | Elbarazi I; Loney T; Yousef S; Elias A | 2017 | BMC Health Serv Res | 10.1186/s12913-017-2319-8 | Psychological outcomes |
| 150 |  | Association between blood lead levels and environmental exposure among Saudi schoolchildren in certain districts of Al-Madinah. | Zolaly MA; Hanafi MI; Shawky N; El-Harbi K; Mohamadin AM | 2012 | Int J Gen Med | 10.2147/IJGM.S28403 | Out of the scope |
| 151 |  | Epidemiological and clinical characteristics of COVID-19 mortality among healthcare workers in Saudi Arabia: A nationwide study. | Aljohar BA; Kilani MA; Bujayr AAA; Humayun T; Alsaffar MJ; Alanazi KH | 2022 | J Infect Public Health | 10.1016/j.jiph.2022.08.005 | Covid related study |
| 152 |  | Prevalence of Workplace Bullying and Its Associated Factors at a Multi-Regional Saudi Arabian Hospital: A Cross-Sectional Study. | Al-Surimi K; Al Omar M; Alahmary K; Salam M | 2020 | Risk Manag Healthc Policy | 10.2147/RMHP.S265127 | Psychological outcomes |
| 153 |  | Assessment of Occupational Burnout among Intensive Care Unit Staff in Jazan, Saudi Arabia, Using the Maslach Burnout Inventory. | Shbeer A; Ageel M | 2022 | Crit Care Res Pract | 10.1155/2022/1298887 | Psychological outcomes |
| 154 |  | Levels of Urinary Biomarkers of Oxidatively Generated Damage to DNA and RNA in Different Groups of Workers Compared to General Population. | Tranfo G; Paci E; Carrieri M; Marchetti E; Sisto R; Gherardi M; Costabile F; Bauleo L; Ancona C; Pigini D | 2019 | Int J Environ Res Public Health | 10.3390/ijerph16162995 | Out of the scope |
| 155 |  | Factors influencing hospital anxiety and depression among emergency department nurses during the COVID-19 pandemic: A multi-center cross-sectional study. | Alzahrani NS; Almarwani AM; Asiri SA; Alharbi HF; Alhowaymel FM | 2022 | Front Psychiatry | 10.3389/fpsyt.2022.912157 | Covid related study |
| 156 |  | Work-Related Challenges among Primary Health Centers Workers during COVID-19 in Saudi Arabia. | Al-Mansour K; Alfuzan A; Alsarheed D; Alenezi M; Abogazalah F | 2021 | Int J Environ Res Public Health | 10.3390/ijerph18041898 | Covid related study |
| 157 |  | The effect of the COVID-19 pandemic on front-line nurses' professional quality of life. | Shaheen MK; Eshah NF; Suliman MM; ALBashtawy MS | 2023 | Nurs Manag (Harrow) | 10.7748/nm.2023.e2074 | Covid related study |
| 158 |  | Musculoskeletal disorders among sonographers in secondary care hospitals in the city of Al-Ahsa, Saudi Arabia. | AlMubarek NA; Al-Otaibi ST; Herzallah HK | 2022 | Work | 10.3233/WOR-205145 | Out of the scope |
| 159 |  | The Prevalence of Depression and Its Associated Risk Factors Among Government Primary School Teachers in Dammam, Khobar, and Qatif (2019-2021): A Cross-Sectional Study. | Al Awwas MY; Alqasem OS; Alhussain HM; Alqahtani AM | 2023 | Cureus | 10.7759/cureus.36271 | Psychological outcomes |
| 160 |  | Exploring the Psychological Stress, Anxiety Factors, and Coping Mechanisms of Critical Care Unit Nurses During the COVID-19 Outbreak in Saudi Arabia. | Ali SAA; Diab SSEM; Elmahallawy EK | 2021 | Front Public Health | 10.3389/fpubh.2021.767517 | Covid related study |
| 161 |  | Prevalence of burnout syndrome and its related risk factors among physicians working in primary health care centers of the Ministry of Health, Al Ahsa region, Saudi Arabia, 2018-2019. | Al-Haddad A; Al-Omar F; Al-Khaleel A; Al-Khalaf A | 2020 | J Family Med Prim Care | 10.4103/jfmpc.jfmpc_743_19 | Psychological outcomes |
| 162 |  | Hoarseness among school teachers: A cross-sectional study from Dammam. | Alrahim AA; Alanazi RA; Al-Bar MH | 2018 | J Family Community Med | 10.4103/jfcm.JFCM_152_17 | Out of the scope |
| 163 |  | Injection safety among primary health care workers in Jazan Region, Saudi Arabia. | Ismail AA; Mahfouz MS; Makeen A | 2014 | Int J Occup Environ Med |  | Out of the scope |
| 164 |  | Preventive Behaviors Towards Covid-19 Pandemic Among Healthcare Providers in Saudi Arabia Using the Protection Motivation Theory. | Mortada E; Abdel-Azeem A; Al Showair A; Zalat MM | 2021 | Risk Manag Healthc Policy | 10.2147/RMHP.S289837 | Covid related study |
| 165 |  | The Effect of Online Teaching on Vocal Health Among Saudi Teachers During COVID-19 Pandemic. | Alarfaj A; Alyahya K; Alutaibi H; Alarfaj M; Alhussain F | 2022 | J Voice | 10.1016/j.jvoice.2022.04.006 | Covid related study |
| 166 |  | Electronic Health Record-Related Stress Among Nurses: Determinants and Solutions. | AlQahtani M; AlShaibani W; AlAmri E; Edward D; Khandekar R | 2021 | Telemed J E Health | 10.1089/tmj.2020.0059 | Psychological outcomes |
| 167 |  | Work-related stress among nursing staff working in government hospitals and primary health care centres. | Alenezi AM; Aboshaiqah A; Baker O | 2018 | Int J Nurs Pract | 10.1111/ijn.12676 | Psychological outcomes |
| 168 |  | Workplace violence among health-care workers in emergency departments of public hospitals in Dammam, Saudi Arabia. | Harthi M; Olayan M; Abugad H; Abdel Wahab M | 2020 | East Mediterr Health J | 10.26719/emhj.20.069 | Psychological outcomes |
| 169 |  | Assessment of biosafety measures in clinical laboratories of Al-Madinah city, Saudi Arabia. | Khabour OF; Al Ali KH; Aljuhani JN; Alrashedi MA; Alharbe FH; Sanyowr A | 2018 | J Infect Dev Ctries | 10.3855/jidc.10081 | Out of the scope |
| 170 |  | Burnout Among School Teachers During the COVID-19 Pandemic in Jazan Region, Saudi Arabia. | Alqassim AY; Shami MO; Ageeli AA; Ageeli MH; Doweri AA; Melaisi ZI; Wafi AM; Muaddi MA; El-Setouhy M | 2022 | Front Psychol | 10.3389/fpsyg.2022.849328 | Psychological outcomes |
| 171 |  | Assessing stress associated with temporomandibular joint disorder through Fonseca's anamnestic index among the Saudi physicians. | Al Hayek SO; Al-Thunayan MF; AlGhaihab AM; AlReshaid RM; Omair A | 2019 | Clin Exp Dent Res | 10.1002/cre2.157 | Psychological outcomes |
| 172 |  | Burnout Among Healthcare Providers in a Comprehensive Cancer Center in Saudi Arabia. | Bany Hamdan A; Alshammary S; Javison S; Tamani J; AlHarbi M | 2019 | Cureus | 10.7759/cureus.3987 | Psychological outcomes |
| 173 |  | Chromosomal aberration analysis among underground water well workers in Saudi Arabia. | AlSuhaibani ES | 2011 | Radiat Prot Dosimetry | 10.1093/rpd/ncq348 | Out of the scope |
| 174 |  | Serum lead levels in civil servicemen and public transport drivers in Makkah City, Saudi Arabia. | Sawas AH; Eldeib AR | 2005 | East Afr Med J | 10.4314/eamj.v82i9.9333 | Full-text unavailable |
| 175 |  | The effect of COVID-19 pandemic on seizure control and self-reported stress on patient with epilepsy. | Alkhotani A; Siddiqui MI; Almuntashri F; Baothman R | 2020 | Epilepsy Behav | 10.1016/j.yebeh.2020.107323 | Covid related study |
| 176 |  | Work-related assaults on nursing staff in riyadh, saudi arabia. | Mohamed AG | 2002 | J Family Community Med |  | Psychological outcomes |
| 177 |  | Psychosocial assessment of voice problems among Saudi teachers. | Malki KH; Mesallam TA | 2012 | J Otolaryngol Head Neck Surg |  | Psychological outcomes |
| 178 |  | Occupational stress among paramedical staff working in primary health care centers in Abha, Saudi Arabia. | Abdelmoneim I | 2003 | J Egypt Public Health Assoc |  | Psychological outcomes |
| 179 |  | Characteristics of healthcare workers with COVID-19: A retrospective descriptive study in a quaternary care center in Riyadh, Saudi Arabia. | Amer HA; Abdallah HA; Alkheledan HS; Gul NS; Altayieb JA; Alsalam M; Tamim H; Alqahtani SA; Soule BM; Memish ZA | 2021 | Ann Med Surg (Lond) | 10.1016/j.amsu.2021.103069 | Covid related study |
| 180 |  | Assessment of noise levels in 200 Mosques in Riyadh, Saudi Arabia. | Al Shimemeri SA; Patel CB; Abdulrahman AF | 2011 | Avicenna J Med | 10.4103/2231-0770.90914 | Out of the scope |
| 181 |  | Emerging Occupational Therapy in Mental Health Practice in Saudi Arabia: A Qualitative Study | Alodan, HA; Squire, R | 2022 | OCCUPATIONAL THERAPY IN MENTAL HEALTH | 10.1080/0164212X.2022.2053634 | Psychological outcomes |
| 182 |  | Risk of occupational radiation exposure for radiation workers involved in interventional endourology in Saudi Arabia | Alashban, Y; Shubayr, N | 2022 | RADIATION PHYSICS AND CHEMISTRY | 10.1016/j.radphyschem.2022.110402 | Out of the scope |
| 183 |  | Occupational radiation doses among nurses working in several medical departments in Saudi Arabia: a five-year national study | Shubayr, N; Alashban, Y | 2021 | RADIOPROTECTION | 10.1051/radiopro/2021028 | Full-text unavailable |
| 184 |  | Annual whole-body occupational radiation exposure in the medical and industrial fields in Saudi Arabia | Shubayr, N; Alashban, Y | 2023 | RADIOPROTECTION | 10.1051/radiopro/2022042 | Full-text unavailable |
| 185 |  | Effects of Job Stress on Health of Saudi Nurses Working in Ministry of Health Hospitals in Qassim Region in KSA | Al Hosis, KF; Mersal, FA; Keshk, LI | 2013 | LIFE SCIENCE JOURNAL-ACTA ZHENGZHOU UNIVERSITY OVERSEAS EDITION |  | Psychological outcomes |
| 186 |  | Impact of emotional intelligence on the stress and safety of construction workers' in Saudi Arabia | Alsulami, H; Serbaya, SH; Rizwan, A; Saleem, M; Maleh, Y; Alamgir, Z | 2023 | ENGINEERING CONSTRUCTION AND ARCHITECTURAL MANAGEMENT | 10.1108/ECAM-06-2021-0481 | Psychological outcomes |
| 187 |  | A cross-sectional investigation of prevalence of occupational burnout in Saudi aviation industry | Ekore, JO; Allui, A; Al Shareef, S; Zawawi, R | 2020 | INTERNATIONAL JOURNAL OF ENGINEERING BUSINESS MANAGEMENT | 10.1177/1847979020946919 | Psychological outcomes |
| 188 |  | From the Prospective of Ergonomics: Estimating Overall Stressors and Task Demands in the Construction Sites in Saudi Arabia Using an Analytical Hierarchy Process (AHP) | Basahel, AM | 2019 | JOURNAL OF SCIENTIFIC & INDUSTRIAL RESEARCH |  | Psychological outcomes |
| 189 |  | Skin Manifestations of Personal Protection Devices and Sanitizers Among COVID-19 Healthcare Providers: A Survey at Southern Region Hospitals, Saudi Arabia | Alfahaad, HA; Alfataih, MTB; Al Adainan, BMM; Al Dundur, AAM; Alyami, AMJ; Alyami, AHM; Almunajjim, MMR; Al Khamsan, HMK; Al Abbas, AMZ; Balhareth, RSM; Alyami, BHH; Alzabin, AAH | 2022 | INTERNATIONAL JOURNAL OF LIFE SCIENCE AND PHARMA RESEARCH | 10.22376/ijpbs/lpr.2022.12.6.SP24.L11-16 | Covid related study |
| 190 |  | Prevalence of physical and verbal violence against physicians and nurses in primary health care centres, Buraidah, Qassim province | Almutairi, MR; Jahan, S | 2022 | WORLD FAMILY MEDICINE | 10.5742/MEWFM.2022.9525038 | Psychological outcomes |
| 191 |  | Exploring Occupational Stress Among Intensive Care Units Nurses in Saudi Arabia Using the Health and Safety Executive Management Standards Indicator Tool | Ageel, M; Shbeer, A | 2022 | NURSING-RESEARCH AND REVIEWS | 10.2147/NRR.S386670 | Psychological outcomes |
| 192 |  | Occupational injuries among radiologist in Saudi Arabia: A cross sectional study | Almutlq, M; Almotairy, F; Alhassoun, A; Al-Hussain, G; Alswayed, K; Alswayah, M; Sandougah, K | 2021 | MEDICAL SCIENCE |  | Out of the scope |
| 193 |  | Skin Damages among healthcare workers caused by enhanced infection-control measures during COVID-19 pandemic - A cross-sectional study from Saudi Arabia | Algaadi, SA; Alanazi, ASA; Alsaadoon, SAH; Alqabbani, AAA; Alomair, SAS | 2022 | MEDICAL SCIENCE | 10.54905/disssi/v26i126/ms333e2217 | Covid related study |
| 194 |  | Occupational stress, anxiety and depression among Egyptian teachers | Desouky, D; Allam, H | 2017 | JOURNAL OF EPIDEMIOLOGY AND GLOBAL HEALTH | 10.1016/j.jegh.2017.06.002 | Psychological outcomes |
| 195 |  | Skin Damages among healthcare workers caused by enhanced infection-control measures during COVID-19 pandemic - A cross-sectional from Saudi Arabia | Algaadi, SA; Alanazi, ASA; Alsaadoon, SAH; Alqabbani, AAA; Alomair, SAS | 2022 | MEDICAL SCIENCE | 10.54905/disssi/v26i126/ms339e2272 | Covid related study |
| 196 |  | Burnout syndrome among nurses working in critical care units at a government hospital, Saudi Arabia | Alanazi, SA; Aloofi, MM; Alsulami, SA; Bagadood, MH | 2022 | BIOSCIENCE RESEARCH |  | Psychological outcomes |
| 197 |  | PATTERN OF WRIST INJURIES IN RIYADH, SAUDI-ARABIA | ALKHAWASHKI, HMI | 1994 | SAUDI MEDICAL JOURNAL |  | Out of the scope |
| 198 |  | Professional exposure and the risk of covid-19 infection among Saudi dentists during the first semester of the pandemic: A cross-sectional study | Alnoury, A; Abuhaimed, T | 2022 | MEDICAL SCIENCE | 10.54905/disssi/v26i119/ms4e1908 | Covid related study |
| 199 |  | Psychological workplace violence against physicians in a large teaching hospital, Eastern Province, Saudi Arabia | AlShamlan, NA; Jayaseeli, N; Aljoudi, AS | 2022 | KUWAIT MEDICAL JOURNAL |  | Psychological outcomes |
| 200 |  | Staff radiation exposure at four radiology departments in the Aseer region of Saudi Arabia | Johary, YH; Aamry, A; Albarakati, S; AlSohaim, A; Aamri, H; Tamam, N; Sulieman, A; Omer, H; Babikir, E; Khandaker, MU; Bradley, D | 2022 | RADIATION PHYSICS AND CHEMISTRY | 10.1016/j.radphyschem.2022.110302 | Full-text unavailable |
| 201 |  | Prevalence of burnout syndrome and its relationship with GPA among medical students at Al Maarefa University, Saudi Arabia, 2022 | Asiri, S; Am, R; Alanazi, MM; Almutairi, AB; Almehmadi, BS; Al-Sharari, KK; Alzahrani, LA; Al-Hussain, LY; Alfawaz, RS; Abudeyah, RS | 2023 | MEDICAL SCIENCE |  | Psychological outcomes |
| 202 |  | Physical activity levels during Covid-19 among nurses at a Saudi teaching hospital: a cross sectional study | Ghamri, RA; Faidah, SO | 2022 | WORLD FAMILY MEDICINE | 10.5742/MEWFM.2022.9525104 | Covid related study |
| 203 |  | Occupational Exposure and Respiratory Tract Infections - At Risk Workers in the International Context | Aasen, TB | 2016 | CURRENT RESPIRATORY MEDICINE REVIEWS | 10.2174/1573398X11666151026221151 | Covid related study |
| 204 |  | Perceived Stress and its Associated Sociodemographic Factors among Physicians Working in Aseer Region of Saudi Arabia | Siddiqui, AF; Al Zaalah, MA; Alqahtani, AA; Alqahtani, MAS | 2017 | JOURNAL OF THE LIAQUAT UNIVERSITY OF MEDICAL AND HEALTH SCIENCES | 10.22442/jlumhs.171610498 | Psychological outcomes |
| 205 |  | Role of Community Pharmacies and Assessment of Occupational Violence Against Him in Saudi Arabia: A Cross Sectional Study Design | Ali, A. | 2023 | Latin American Journal of Pharmacy |  | Psychological outcomes |
| 206 |  | Burnout and Resilience among Respiratory Therapy (RT) Students during Clinical Training in Saudi Arabia: A Nationwide Cross-Sectional Study | Siraj, R.A.; Aldhahir, A.M.; Alqahtani, J.S.; Almarkhan, H.M.; Alghamdi, S.M.; Alqarni, A.A.; Alhotye, M.; Algarni, S.S.; Alahmadi, F.H.; Alahmari, M.A. | 2022 | International Journal of Environmental Research and Public Health | 10.3390/ijerph192013047 | Psychological outcomes |
| 207 |  | A web-based cross-sectional study assessing the impact of COVID-19 on the mental health of radiology staff in Saudi Arabia | Abdeen, R. | 2022 | PLoS ONE | 10.1371/journal.pone.0265873 | Covid related study |
| 208 |  | The Impact of Job Stress, Role Ambiguity and Work‚ÄìLife Imbalance on Turnover Intention during COVID-19: A Case Study of Frontline Health Workers in Saudi Arabia | Alblihed, M.; Alzghaibi, H.A. | 2022 | International Journal of Environmental Research and Public Health | 10.3390/ijerph192013132 | Covid related study |
| 209 |  | Assessment of occupational exposure from PET and PET/CT scanning in Saudi Arabia | Omer, H.; Salah, H.; Tamam, N.; Mahgoub, O.; Sulieman, A.; Ahmed, R.; Abuzaid, M.; Saad, I.E.; Almogren, K.S.; Bradley, D.A. | 2023 | Radiation Physics and Chemistry | 10.1016/j.radphyschem.2022.110642 | Full-text unavailable |
| 210 |  | Assessing compassion satisfaction, compassion fatigue, stress and job satisfaction among nurses | Shdaifat, E. | 2023 | SA Journal of Industrial Psychology | 10.4102/sajip.v49i0.2082 | Psychological outcomes |
| 211 |  | Prevalence, characteristics, contributing factors, work sector, and impact of work-related musculoskeletal disorders among otorhinolaryngology practitioners in Saudi Arabia: A cross-sectional study | Alshehri, S. | 2022 | Work | 10.3233/WOR-211054 | Out of the scope |
| 212 |  | Factors influencing the mental health of caregivers of children with cerebral palsy | Kouther, D.A.; Shakir, M.O.; Alhumaidah, R.A.; Jamaluddin, H.A.; Jaha, A.Y.; Alshumrani, M.J.; Hakami, A.Y. | 2022 | Frontiers in Pediatrics | 10.3389/fped.2022.920744 | Psychological outcomes |
| 213 |  | Cyberloafing behaviours in nursing: The role of nursing stressors | Alqahtani, N.; Innab, A.; Alammar, K.; Alkhateeb, R.; Kerari, A.; Alharbi, M. | 2022 | International Journal of Nursing Practice | 10.1111/ijn.13079 | Out of the scope |
| 214 |  | Risk of COVID-19 in healthcare workers working in intensive care setting | Alshamrani, M.M.; El-Saed, A.; Arabi, Y.M.; Zunitan, M.A.; Farahat, F.M.; Bonnie, H.B.; Matalqa, M.; Othman, F.; Almohrij, S. | 2022 | American Journal of Infection Control | 10.1016/j.ajic.2022.01.003 | Covid related study |
| 215 |  | Physiological impacts on construction workers under extremely hot and humid weather | Moohialdin, A.; Trigunarsyah, B.; Islam, M.S.; Siddiqui, M.K. | 2022 | International Archives of Occupational and Environmental Health | 10.1007/s00420-021-01785-w | Full-text unavailable |
| 216 |  | Knowledge and practice of Protective Personal Equipment (PPE) among healthcare providers in Saudi Arabia during the early stages of COVID-19 pandemic in 2020 | Ashoor, M.; Alshammari, S.; Alzahrani, F.; Almulhem, N.; Almubarak, Z.; Alhayek, A.; Alrahim, A.; Alardhi, A. | 2021 | Journal of Preventive Medicine and Hygiene | 10.15167/2421-4248/jpmh2021.62.4.2177 | Covid related study |
| 217 |  | Psychological and Occupational Pressure Among Female Teachers in Light of the Coronavirus Disease Pandemic and Coping Strategies | Al-Rashidi, A.H. | 2022 | European Journal of Educational Research | 10.12973/eu-jer.11.1.533 | Covid related study |
| 218 |  | Changes in healthcare workers' knowledge, attitudes, practices, and stress during the COVID-19 pandemic | Temsah, M.-H.; Al Huzaimi, A.; Alrabiaah, A.; Alamro, N.; Al-Sohime, F.; Al-Eyadhy, A.; Alhasan, K.; Kari, J.A.; Alhaboob, A.; Alsalmi, A.; Almuhanna, W.; Almaghlouth, I.; Aljamaan, F.; Halwani, R.; Saddik, B.; Barry, M.; Al-Zamil, F.; Alhadi, A.N.; Al-Subaie, S.; Jamal, A.; Somily, A.M. | 2021 | Medicine (United States) | 10.1097/MD.0000000000025825 | Covid related study |
| 219 |  | COVID-19 psychological impact on health care workers in saudi arabia | Alamri, H.S.; Mousa, W.F.; Algarni, A.; Megahid, S.F.; Bshabshe, A.A.; Alshehri, N.N.; Alsamghan, A.; Alsabaani, A.; Mirdad, R.T.; Alshahrani, A.M.; Gadah, A.A.; Alshehri, A.A. | 2021 | International Journal of Environmental Research and Public Health | 10.3390/ijerph18116076 | Covid related study |
| 220 |  | Psychological impact of the COVID-19 pandemic on healthcare professionals in the Al Ahsa Region, Saudi Arabia | Almaqhawi, A.; Khan, A.S.; Albarqi, M.; Almulhim, A.; Ali, S.I.; Elbarbary, H.S. | 2022 | Family Medicine and Primary Care Review | 10.5114/fmpcr.2022.113010 | Covid related study |
| 221 |  | Assessment of anxiety level and sleep quality of medical staff treating patients with COVID-19 | Alboghdadly, A.; Saadh, M.J.; Kharshid, A.M.; Shaalan, M.S.; Alshawwa, S.Z. | 2022 | European Review for Medical and Pharmacological Sciences | 10.26355/eurrev_202201_27783 | Covid related study |
| 222 |  | Depression among physicians and other medical employees involved in the COVID-19 outbreak: A cross-sectional study | Alghasab, N.S.; Aljadani, A.H.; Almesned, S.S.; Hersi, A.S. | 2021 | Medicine (United States) | 10.1097/MD.0000000000025290 | Covid related study |
| 223 |  | Prevalence of workplace-related violence among otorhinolaryngology residents in Riyadh, Saudi Arabia | Alsaleh, A.S.; Almotairi, A.I.; Alim, B.M.; Alroqi, A.S. | 2021 | Journal of Nature and Science of Medicine | 10.4103/jnsm.jnsm_102_20 | Psychological outcomes |
| 224 |  | Prevalence of Work-Related Stress and its Associated Factors among Healthcare Professionals at Jazan Region, Saudi Arabia | Alrashidi, N.A.; Mahdi, G.H.A.; Abo Elmagd, M.H.; Alshmemri, M.S.; Alghabbashi, M.T. | 2022 | Journal of Pharmaceutical Negative Results | 10.47750/pnr.2022.13.S01.144 | Psychological outcomes |
| 225 |  | Healthcare Workers‚Äô SARS-CoV-2 Omicron Variant Uncertainty-Related Stress, Resilience, and Coping Strategies during the First Week of the World Health Organization‚Äôs Alert | Temsah, M.-H.; Alenezi, S.; Alarabi, M.; Aljamaan, F.; Alhasan, K.; Assiri, R.; Bassrawi, R.; Alshahrani, F.; Alhaboob, A.; Alaraj, A.; Alharbi, N.S.; Alrabiaah, A.; Halwani, R.; Jamal, A.; Abdulmajeed, N.; Alfarra, L.; Almashdali, W.; Al-Eyadhy, A.; Alzamil, F.; Alsubaie, S.; Barry, M.; Memish, Z.A.; Al-Tawfiq, J.A. | 2022 | International Journal of Environmental Research and Public Health | 10.3390/ijerph19041944 | Covid related study |
| 226 |  | The Influence of Cultural and Psychological Factors on Mental Health Status during COVID-19 in Saudi Arabia | Alqahtani, A. | 2022 | Open Psychology Journal | 10.2174/18743501-v15-e2202070 | Covid related study |
| 227 |  | ASSESSMENT OF WORKPLACE VIOLENCE AMONGST PSYCHIATRIC NURSES | Alharthi, M.S. | 2022 | New Armenian Medical Journal | 10.59936/18290825-2022.16.3-15 | Psychological outcomes |
| 228 |  | Stress and psychological resilience among general surgery residents during COVID-19 pandemic | Aljehani, Y.M.; Othman, S.A.; Telmesani, N.K.; Alghamdi, R.A.; AlBuainain, H.M.; Alghamdi, Z.M.; Zakaria, H.M.; Alreshaid, F.T.; Busbait, S.A.; Alqarzaie, A.A.; Alharbi, T.M.; Alnajim, R.K. | 2020 | Saudi Medical Journal | 10.15537/smj.2020.12.25577 | Covid related study |
| 229 |  | Latent tuberculosis infection among healthcare workers using Quantiferon-TB Gold-Plus in a country with a low burden for tuberculosis: Prevalence and risk factors | Almohaya, A.; Aldrees, A.; Akkielah, L.; Hashim, A.T.; Almajid, F.; Binmoammar, T.; Barry, M.A. | 2020 | Annals of Saudi Medicine | 10.5144/0256-4947.2020.191 | Out of the scope |
| 230 |  | The Impact of Occupational Stress and Burnout among Employees of Different Age Groups on Job Satisfaction: Evidence from Saudi Arabia and Bahrain | George, S.; Al Saffar, E.M.; Almohammedsaleh, Z. | 2021 | 2021 International Conference on Sustainable Islamic Business and Finance, SIBF 2021 | 10.1109/IEEECONF53626.2021.9686330 | Psychological outcomes |
| 231 |  | Sleep disturbances among physicians during COVID-19 pandemic | Alnofaiey, Y.H.; Alshehri, H.A.; Alosaimi, M.M.; Alswat, S.H.; Alswat, R.H.; Alhulayfi, R.M.; Alghamdi, M.A.; Alsubaie, R.M. | 2020 | BMC Research Notes | 10.1186/s13104-020-05341-6 | Covid related study |
| 232 |  | Healthcare workers experience in dealing with Coronavirus (COVID-19) pandemic | Almaghrabi, R.H.; Alfaradi, H.; Hebshi, W.A.A.; Albaadani, M.M. | 2020 | Saudi Medical Journal | 10.15537/SMJ.2020.6.25101 | Covid related study |
| 233 |  | Residents' perceived impact of covid-19 on saudi ophthalmology training programs-a survey | Alahmadi, A.S.; Alhatlan, H.M.; Helayel, H.B.; Khandekar, R.; Habash, A.A.; Al-Shahwan, S. | 2020 | Clinical Ophthalmology | 10.2147/OPTH.S283073 | Covid related study |
| 234 |  | Assessing healthcare Workers‚Äô knowledge, emotions and perceived institutional preparedness about COVID-19 pandemic at Saudi hospitals in the early phase of the pandemic | Alreshidi, N.M.; Haridi, H.K.; Alaseeri, R.; Garcia, M.; Gaspar, F.; Alrashidi, L. | 2020 | Journal of Public Health Research | 10.4081/jphr.2020.1936 | Covid related study |
| 235 |  | Evaluation of knowledge, attitude, and practices toward the outbreak pandemic (COVID-19) Virus Disease among ophthalmologists: A cross-sectional study | ALBalawi, H.B.; Alali, N.M. | 2020 | Middle East African Journal of Ophthalmology | 10.4103/meajo.MEAJO_219_20 | Covid related study |
| 236 |  | Perception and attitude of healthcare workers in Saudi Arabia with regard to Covid-19 pandemic and potential associated predictors | Abolfotouh, M.A.; Almutairi, A.F.; Banimustafa, A.A.; Hussein, M.A. | 2020 | BMC Infectious Diseases | 10.1186/s12879-020-05443-3 | Covid related study |
| 237 |  | Work-related musculoskeletal disorders among dental staff in Armed Force Hospital in Dhahran, Saudi Arabia | Alzayani, M.; Salama, K.; Zafar, M. | 2021 | International Journal of Preventive Medicine | 10.4103/ijpvm.IJPVM_136_20 | Out of the scope |
| 238 |  | Levels of Physical Activity and Prevalence of Musculoskeletal Disorders Among Physicians in Saudi Arabia Post COVID-19 Lockdown: An Epidemiological Cross-Sectional Analysis | AlOmar, R.S. | 2021 | Journal of Primary Care and Community Health | 10.1177/21501327211040359 | Covid related study |
| 239 |  | Stress and coping among consultant physicians working in Saudi Arabia | Alosaimi, F.D.; Alawad, H.S.; Alamri, A.K.; Saeed, A.I.; Aljuaydi, K.A.; Alotaibi, A.S.; Alotaibi, K.M.; Alfaris, E.A. | 2018 | Annals of Saudi Medicine | 10.5144/0256-4947.2018.214 | Psychological outcomes |
| 240 |  | Screening of latent tuberculosis infection among health care workers working in Hajj pilgrimage area in Saudi Arabia, using interferon gamma release assay and tuberculin skin test | Bukhary, Z.A.; Amer, S.M.; Emara, M.M.; Abdalla, M.E.; Ali, S.A. | 2018 | Annals of Saudi Medicine | 10.5144/0256-4947.2018.90 | Out of the scope |
| 241 |  | Perceived stress and coping strategies among ICU nurses in government tertiary hospitals in Saudi Arabia: A cross-sectional study | Alharbi, H.; Alshehry, A. | 2019 | Annals of Saudi Medicine | 10.5144/0256-4947.2019.48 | Psychological outcomes |
| 242 |  | Stress and coping strategies among Saudi nursing students during clinical education | Al-Gamal, E.; Alhosain, A.; Alsunaye, K. | 2018 | Perspectives in Psychiatric Care | 10.1111/ppc.12223 | Psychological outcomes |
| 243 |  | The impact of work-related stress on medication errors in Eastern Region Saudi Arabia | Salam, A.; Segal, D.M.; Abu-Helalah, M.A.; Gutierrez, M.L.; Joosub, I.; Ahmed, W.; Bibi, R.; Clarke, E.; Al Qarni, A.A. | 2019 | International Journal for Quality in Health Care | 10.1093/intqhc/mzy097 | Psychological outcomes |
| 244 |  | Incidence of needlestick injuries in oral surgery clinic among dental students: A retrospective study | Ali, F.M.; Al-Iryani, G.M.; Mdabesh, H.Y.; Essa, A.A.; Nammazi, A.M.; Kariri, M.A.; Somaili, D.M.Y. | 2018 | World Journal of Dentistry | 10.5005/jp-journals-10015-1502 | Wrong patient population |
| 245 |  | A multi-faceted approach of a nursing led education in response to MERS-CoV infection | Al-Tawfiq, J.A.; Rothwell, S.; Mcgregor, H.A.; Khouri, Z.A. | 2018 | Journal of Infection and Public Health | 10.1016/j.jiph.2017.08.006 | Covid related study |
| 246 |  | High Prevalence of MERS-CoV Infection in Camel Workers in Saudi Arabia | Alshukairi, A.N.; Zheng, J.; Zhao, J.; Nehdi, A.; Baharoon, S.A.; Layqah, L.; Bokhari, A.; Al Johani, S.M.; Samman, N.; Boudjelal, M.; Eyck, P.T.; Al-Mozaini, M.A.; Perlman, S.; Alagaili, A.N. | 2018 | mBio | 10.1128/MBIO.01985-18 | Covid related study |
| 247 |  | Assessment of neck pain and cervical mobility among female computer workers at Hail University | Mohammad, W.S.; Hamza, H.H.; ElSais, W.M. | 2015 | International Journal of Occupational Safety and Ergonomics | 10.1080/10803548.2015.1017952 | Out of the scope |
| 248 |  | Organizational role stress among university academic staff: Reflect and influence | Al-Kahtani, N.S. | 2017 | Man in India |  | Psychological outcomes |
| 249 |  | Sparse evidence of MERS-CoV infection among animal workers living in Southern Saudi Arabia during 2012 | Memish, Z.A.; Alsahly, A.; Masri, M.al.; Heil, G.L.; Anderson, B.D.; Peiris, M.; Khan, S.U.; Gray, G.C. | 2015 | Influenza and other Respiratory Viruses | 10.1111/irv.12287 | Covid related study |
| 250 |  | Healthcare workers emotions, perceived stressors and coping strategies during a MERS-CoV outbreak | Khalid, I.; Khalid, T.J.; Qabajah, M.R.; Barnard, A.G.; Qushmaq, I.A. | 2016 | Clinical Medicine and Research | 10.3121/cmr.2016.1303 | Covid related study |
| 251 |  | Investigation of anti-middle east respiratory syndrome antibodies in blood donors and slaughterhouse workers in Jeddah and Makkah, Saudi Arabia, Fall 2012 | Aburizaiza, A.S.; Mattes, F.M.; Azhar, E.I.; Hassan, A.M.; Memish, Z.A.; Muth, D.; Meyer, B.; Lattwein, E.; M√ºller, M.A.; Drosten, C. | 2014 | Journal of Infectious Diseases | 10.1093/infdis/jit589 | Covid related study |
| 252 |  | Stress, shift duty, and eating behavior among nurses in Central Saudi Arabia | Almajwal, A.M. | 2016 | Saudi Medical Journal | 10.15537/smj.2016.2.13060 | Psychological outcomes |
| 253 |  | Effect of exposure to cement dust on Fractional Exhaled Nitric Oxide (FeNO) in non-smoking cement mill workers | Meo, S.A.; Alsaaran, Z.F.; Alshehri, M.K. | 2014 | European Review for Medical and Pharmacological Sciences |  | Out of the scope |
| 254 |  | Hand dermatitis among nurses at a University Hospital in Saudi Arabia | Kokandi, A.A. | 2017 | Biomedical Research (India) |  | Out of the scope |
| 255 |  | Effect of duration of exposure to polluted air environment on lung function in subjects exposed to crude oil spill into sea water | Meo, S.A.; Al-Drees, A.M.; Rasheed, S.; Meo, I.M.; Khan, M.M.; Al-Saadi, M.M.; Alkandari, J.R. | 2009 | International Journal of Occupational Medicine and Environmental Health | 10.2478/v10001-009-0007-6 | Wrong patient population |
| 256 |  | Violence against primary health care workers in Al-Hassa, Saudi Arabia | El-Gilany, A.-H.; El-Wehady, A.; Amr, M. | 2010 | Journal of Interpersonal Violence | 10.1177/0886260509334395 | Psychological outcomes |
| 257 |  | Electronic Health Record-Related Stress Among Nurses: Determinants and Solutions. | AlQahtani M; AlShaibani W; AlAmri E; Edward D; Khandekar R | 2021 | Telemed J E Health | 10.1089/tmj.2020.0059 | Psychological outcomes |
| 258 |  | Epidemiology of H1N1 (2009) influenza among healthcare workers in a tertiary care center in Saudi Arabia: A 6-month surveillance study | Balkhy, H.H.; El-Saed, A.; Sallah, M. | 2010 | Infection Control and Hospital Epidemiology | 10.1086/656241 | Covid related study |
| 259 |  | Are orthopedic surgeons prone to burnout? | Sadat-Ali, M.; Al-Habdan, I.M.; Al-Dakheel, D.A.; Shriyan, D. | 2005 | Saudi Medical Journal |  | Psychological outcomes |
| 260 |  | Violence exposure among health care professionals in Saudi public hospitals: A preliminary investigation | Algwaiz, W.M.; Alghanim, S.A. | 2012 | Saudi Medical Journal |  | Psychological outcomes |
| 261 |  | Health impacts on workers in landfill in Jeddah City, Saudi Arabia. | Abdou, M.H. | 2007 | The Journal of the Egyptian Public Health Association |  | Out of the scope |
| 262 |  | Infectious diseases among food handlers. | Salem, K.A. | 1998 | The Journal of the Egyptian Public Health Association |  | Full-text unavailable |
| 263 |  | Prevalence of ocular injuries, conjunctivitis and use of eye protection among dental personnel in Riyadh, Saudi Arabia | Al Wazzan, K.A.; Almas, K.; Al Qahtani, M.Q.; Al Shethri, S.E.; Khan, N. | 2001 | International Dental Journal | 10.1002/j.1875-595x.2001.tb00828.x | Out of the scope |
| 264 |  | Hematological findings in male x-ray technicians | Meo, S.A. | 2004 | Saudi Medical Journal |  | Full-text unavailable |
| 265 |  | Occupational stress in different organizations: A Saudi Arabian survey | Ben-Bakr, K.A.; Al-Shammari, I.S.; Jefri, O.A. | 1995 | Journal of Managerial Psychology | 10.1108/02683949510085956 | Psychological outcomes |
| 266 |  | A study on health hazard of insecticides on insecticide sprayers in Mecca | El Sewefy, A.Z.; Ghali, M.A.; Sabry, M. | 1976 | Ain Shams Medical Journal |  | Full-text unavailable |
