# Supplementary material for: Occupational health in the Gulf Cooperation Council (GCC): A systematic review and call for comprehensive policy development
Source: PLoS One. 2024 Dec 10;19(12):e0312251. doi: 10.1371/journal.pone.0312251 (PMC11630603; doi:10.1371/journal.pone.0312251)
Supplement: S1 Table — The current systematic review follows PRISMA guidelines and focuses on occupational health research in the six GCC countries. A comprehensive English-language search was conducted in Scopus, PubMed, and Web of Science, yielding relevant studies published up to October 2023. Only primary research published in peer-reviewed journals was included, with a focus on physical, chemical, biological, and ergonomic factors in occupational health. Studies specifically related to COVID-19, psychosocial hazards, and gray literature were excluded. The review protocol was registered with PROSPERO, and the inclusion process involved two independent reviewers (K.A. and M.M.) between November 2023 and January 2024. S1 Table provides a detailed summary of the findings from the selected studies. (DOCX) [file pone.0312251.s002.docx]

Table S1: comprehensive table exhibiting the results of the selected studies.

The current systematic review follows PRISMA guidelines and focuses on occupational health research in the six GCC countries. A comprehensive English-language search was conducted in Scopus, PubMed, and Web of Science, yielding relevant studies published up to October 2023. Only primary research published in peer-reviewed journals was included, with a focus on physical, chemical, biological, and ergonomic factors in occupational health. Studies specifically related to COVID-19, psychosocial hazards, and gray literature were excluded. The review protocol was registered with PROSPERO, and the inclusion process involved two independent reviewers (K.A. and M.M.) Supplementary Table S1 provides a detailed summary of the findings from the selected studies.

| **Country** | **Hazard category** | **Hazard type** | **Population** | **Study Design** | **Main Findings** | **Reference** |
| --- | --- | --- | --- | --- | --- | --- |
| **Bahrain** | Physical | Heat Stress | 1111 | Cross sectional / Cohort | Majority of workers suffering from climate related conditions work outdoors.  Climate related conditions are not associated with age, nationality and place of work. | [24] |
|  |  | Physical Injury | 42 | Cross sectional / Cohort | Most of the injuries (95.2%) occurred due to workers not using safety eyewear.  Most injured workers were from construction and  welding occupations. | [23] |
|  |  | Needlestick Injury | 150 | Cross sectional / Cohort | Most needle stick injuries occurred among nurses.  Only half of the healthcare workers affected by needle stick injuries attended an annual training program.  27% of doctors thought that "I should not recap a  needle" (p = 0.030). | [22] |
|  | Chemical | Gas exposure | 100 | Cross sectional /  Cohort | Mean levels of Carboxyhemoglobin (%COHb) in smokers and non smokers after work exceeded  recommended levels by WHO and NIOSH. | [25] |
|  | Ergonomics | Musculoskeletal Disorders | 200 | Cross sectional / Cohort | Lower back pain and neck pain were most common affected body parts.  Work-related MSds were significantly corre- lated with occupation, number of years in the job, and age. | [20] |
|  |  | Musculoskeletal Disorders | 550 | Cross sectional / Cohort | Lower back pain, shoulder and neck pain were most prevalent.  The prevalence of low back symptoms was  significantly associated with age. | [21] |
|  | Miscellaneous | Cardiovascular Disease | 1139 | Cross sectional / Cohort | Half of the participants did not engage in any physical activity.  21.6% and 23.3% had elevated systolic and diastolic  blood pressures, respectively. | [26] |
| **Oman** | Physical | Physical Injury | 150 | Cross sectional /  Cohort | Most of the injuries were contusiuon and sprains. Injury rate increased with age. | [31] |
|  |  | Physical Injury | 170 | Cross sectional / Cohort | Foreign body to the eye was the most common type of injury.  Injury to the upper extremities accounted for the largest percentage.  The average injury rate per 1000 exposed workers per year was 19.8. | [33] |
|  |  | Heat Stress | 201 | Cross sectional / Cohort | Average heart rate and urine specific gravity increased in the first 4 hours of shift work.  In the second 4 hours of shift work, urine specific gravity stabilized compared  to the first 4 hours. | [32] |
|  |  | Radiation | 70 | Cross sectional / Cohort | The derived average effective dose for a technologist throughout the PET/CT procedure was 4.17 mSv per study or 5 mSv/y, which is below the 20 mSv/y annual limit recommended by the International  Commission on Radiological Protection for  professionals in medical imaging. | [34] |
|  |  | Radiation | 116 | Cross sectional /  Cohort | The findings revealed predominantly lower Dose Investigation Levels (DILs) in almost all groups,  except nuclear medicine. | [35] |
|  |  | Noise | 312 | Cross sectional / Cohort | The TWA recorded for the workers was above the accepted limit in some departments, namely, cabin appearance, ramp, line maintenance and hangar.  NIHL was common among participants aged 40 or above. | [37] |
|  |  | Vibration | 1459 | Case series | The evaluation revealed significant risks associated with workplace hazards, notably the exposure to vibration when operating pneumatic jack hammers and plate compactors.  These vibrations have the potential to cause Hand-  Arm vibration syndrome among exposed workers. | [38] |
|  |  | Radiation | 19 | Cross sectional / Cohort | The findings showed that the average annual effective whole-body and extremity doses were  considerably below the recommended annual dose  limits. | [36] |
|  | Chemical | Pesticide | 74 | Cross sectional / Cohort | The underutilization of Personal Protective Equipment was commonplace.  A considerable number of workers reported health  symptoms such as skin irritation (70.3%), burning | [39] |
|  |  |  |  |  | sensations (39.2%), headaches (33.8%), vomiting  (29.7%), and excessive salivation (21.6%). |  |
|  | Biological | Animal related | 282 | Cross sectional / Cohort | Butchers were notably more susceptible to developing Crimean-Congo hemorrhagic fever. A higher prevalence among non-Omani citizens  compared to Omani citizens. | [40] |
|  |  | Animal related | 1 | Case series | A veterinary assistant after a needlestick injury from a bull infected with brucellosis, exhibited symptoms of fever, weight loss, insomnia, and joint pain,  despite undergoing a 21-day course of doxycycline as  a postexposure prophylaxis. | [41] |
|  | Ergonomics | Musculoskeletal Disorders | 32 | Cross sectional /  Cohort | 94% of participants deemed the workdays long, 79% were discontent with schedules, and 61% found  summer conditions intolerable. | [27] |
|  |  | Musculoskeletal Disorders | 150 | Cross sectional / Cohort | 45% of participants were using non-adjustable chairs and 90% were using computers over 4 hours daily, with prevalent issues of eyestrain (58%), shoulder  pain (45%), and back pain (43%). | [28] |
|  |  | Musculoskeletal Disorders | 40 | Cross sectional / Cohort | Intervention results indicated notable declines in pain and various complaints, with pain reducing from 82.5% to 35%, headaches from 58% to 25%, and  wrist pain from 80% to 38% by the end of the study. | [30] |
|  |  | Musculoskeletal Disorders | 128 | Cross sectional / Cohort | Extended working hours have substantial impacts on family life (s=54%), performance (s=68%),  productivity, and health of the workers. | [29] |

| **Qatar** | Physical | Needlestick Injury | 1022 | Cross sectional / Cohort | 20.9% of the healthcare workers experienced needlestick injuries.  Female reported a higher rate of injuries at 69.2% compared to their male counterparts.  The highest incidences were in medicine (13.1%), followed by the laboratory (9.3%), emergency  (8.9%). | [45] |
| --- | --- | --- | --- | --- | --- | --- |
|  |  | Physical Injury | 1952 | Cross sectional / Cohort | Nearly half of the injuries occurred at work (49.4%), with neck injuries proving more severe (10.2%) than head injuries (7.3%). | [46] |
|  |  | Physical Injury | 53366 | Cross sectional / Cohort | Head injuries were the most common for both genders, with the highest occurrences linked to road traffic accidents (21.2%), industrial machinery  (16.4%), and construction incidents (15.5%). | [47] |
|  |  | Heat Stress | 1354 | Cross sectional / Cohort | A significant correlation between high afternoon heat levels (WBGT) and cardiovascular disease (CVD) mortality, with a Pearson correlation coefficient (r) of  0.54, p < 0.01, was reported. | [48] |
|  |  | Needlestick Injury | 130 | Cross sectional / Cohort | The majority of injuries occurred in operating theaters and involved hollow-bore needles, typically post-use or during surgical interventions.  Adequate HBV immunity was found in 95.4% of  healthcare personnel, and no seroconversion to HBV, HCV, or HIV was observed in the follow-up period. | [49] |
|  |  | Sharp Injury | 114 | Cross sectional / Cohort | 68.42% of the sample had experienced a sharp injury within the previous year, primarily due to dental  explorers and injection needles.  84.47% of the injured did not report them. | [50] |
|  |  | Unspecified Occupational Injury | 2174828 | Cross sectional / Cohort | There were 506 severe injuries, mainly from falls and heavy objects, marking 23.26 per 100,000 workers, and 37,601 mild to moderate injuries, representing  1,728.91 per 100,000 workers. | [51] |
|  | Chemical | Pesticide | 125 | Cross sectional / Cohort | Less than 2% of workers knew the names of the pesticides, and around a third were uncertain about the appropriate amounts to apply.  The majority mixed pesticides without utilizing protective clothing, and 18% were unaware of the potential health risks. | [52] |
|  | Ergonomics | Shift Work | 2562 | Cross sectional / Cohort | Incidence of CHD in shift workers at 13.5% compared to 7.1% in daytime workers, with a risk ratio (RR) of  1.65, 95% CI=(1.38–1.97), (p < 0.001).  Shift workers demonstrated a doubled prevalence of heart conditions at 4.1%, as opposed to 2.0% in daytime workers, RR=1.62(1.20–2.18); (p = 0.0042). | [42] |
|  |  | Musculoskeletal Disorders | 254 | Cross sectional / Cohort | Lower back pain significantly impacted daily  activities and work, with 76.8% of nurses reporting work stoppages due to lower back pain.  Sports practice, office work, and exposure to physical | [43] |

|  |  |  |  |  | stress was identified as significant predictors of lower back pain. |  |
| --- | --- | --- | --- | --- | --- | --- |
|  |  | Musculoskeletal Disorders | 479 | Cross sectional / Cohort | Sedentary behavior was found to be significantly associated with experiencing either lower or upper back pain [aOR = 1.74, 95% CI (1.19, 2.57)]  More than half (61.2%) of respondents reported back pain. | [44] |
|  | Miscellaneous | Cardiovascular Disease | 369 | Cross sectional / Cohort | Firefighters despite having a higher smoking  prevalence, demonstrated a significantly lower mean risk percentage for CHD than compared to non-  firefighters.  Non-firefighters showed a higher prevalence of diabetes. | [53] |
|  |  | Unspecified Occupational Injury | 6555 | Cross sectional / Cohort | 30.7% of the total injuries were related to occupational setting.  Non-fatal injury rate was 37.34 per 100,000 workers  and a fatal injury rate was 1.58 per 100,000 workers. | [54] |
| **Kuwait** | Physical | Radiation | 4 | Case series | Radium-226 activity decreased over time but its  byproducts' activity increased. | [61] |
|  |  | Radiation | 906 | Cross sectional / Cohort | Though annual doses for workers were relatively low, they remained higher than global averages.  Elevated exposures were especially observed among radiology professionals. | [62] |
|  |  | Pneumoconiosis | 1 | Case series | The first case of asbestosis in Kuwait was reported after medical examination of workers in one of the only asbestos-cement pipe factories.  This factory had an asbestosis prevalence rate of  0.32%. | [69] |
|  |  | Heat Stress | 22 | Case series | 22 male outdoor workers (aged 24-50), were diagnosed with hypokalemic paralysis.  Dehydration was an important precipitating factor in  all of the cases. | [70] |
|  |  | Radiation | 1780 | Cross sectional / Cohort | Average annual exposure for all roles was well below the ICRP limit.  Significant reductions in Hp(10) and Hp(0.07) were observed in 2009 for NM technologists and DR  technologists (p < 0.001). | [63] |
|  |  | Needlestick Injury | 249 | Cross sectional / Cohort | Nurses were most commonly affected followed by doctors.  Most common type pf exposure was needlestick injury.  Exposures mainly occurred in inpatient wards. | [64] |
|  |  | Noise  **School children** | 18 | Cross sectional / Cohort | Secondary schools were the most affected with noise related issues.  Most common health issues were hearing problems and headaches.  Kuwaiti classrooms had high levels of noise  compared to global standards. | [67] |
|  |  | Radiation | 11 | Cross sectional / Cohort | Average annual eye radiation dose was 4.5 mSv. Annual average wole-body and skin doses were measured at 4.0 mSv and 120 mSv respectively. All measurements were within the ICRP 2014  standards. | [65] |
|  |  | Noise | 3474 | Cross sectional / Cohort | Prevalence of Occupational Noise Induced Hearing Loss (ONIHL) was 20.4%.  Majority of the affected workers were men and non-  Kuwaitis. | [68] |
|  |  | Radiation | 60 | Cross sectional / Cohort | Recorded doses for right eye were between 12.6 -  23.6 μSv and left eye ranged from 18.5 to 30.3 μSv. The dose limit of 20 mSv/year to the eyes can be exceeded for interventional cardiologists in some  situations. | [66] |
|  |  | Heat Stress | - | Cross sectional / Cohort | When temperatures reached 39.4°C, the risk of injuries was increased by 44%.  Risk of heat related injuries was significant despite  the imposition of midday break. | [71] |
|  | Chemical | Chemical exposure | 209 | Case series | 77 of the 156dermatitis patients came in contact with cement for the first time in Kuwait.  Temperature and humidity can be linked to increase  in dermatitis prevalence. | [72] |
|  |  | Pesticide | 38 | Case control | Regular pesticide applicators showed lung function variations and decreased ChE activity. | [73] |

|  |  |  |  |  | RBC ChE activity of affected workers recovered over time. |  |
| --- | --- | --- | --- | --- | --- | --- |
|  |  | Gas exposure | 460 | Cross sectional / Cohort | Commonly prevalent morbidities were eeys related complaints, sneezing, tremors and skin complains. Workers were more likely to report health related issues if they were overweight, married and on  current job for more than one year. | [74] |
|  | Ergonomics | Musculoskeletal Disorders | 143 | Cross sectional / Cohort | The lifetime prevalence of work-related LBP was 70% (61.8% in males and 74.2% in females).  The main pain site was lower back and buttocks.  Half of the therapists reported work activities being affected by lower back pain. | [55] |
|  |  | Musculoskeletal Disorders | 344 | Cross sectional / Cohort | Lifetime prevalence of lower back pain (LBP) in the sample was 70.9%, and point prevalence of LBP was 21.5%.  Factors associated with acute LBP included direct patient contact (p = 0.015) | [56] |
|  |  | Musculoskeletal Disorders | 350 | Cross sectional / Cohort | Most common compliants were in the lower back and neck.  The incidence of WMSDs wasn't significantly linked  to factors like gender or age, except in specific areas. | [57] |
|  |  | Musculoskeletal Disorders | 470 | Cross sectional / Cohort | 88 (18.7%) of the respondents self reported carpal tunnel syndrome (CTS).  CTS was also associated with key symptoms and  impairment in daily activities (p < 0.05). | [58] |
|  |  | Musculoskeletal Disorders | 312 | Cross  sectional / Cohort | The lower back and muscle spasm were the most  common area of the body injured and type of injury, respectively. | [59] |
|  |  | Musculoskeletal Disorders | 186 | Cross sectional / Cohort | The results showed that 88 (47%) of responding dentists experienced WMSDs.  A significant association was found between rating of  pain and lost days from work (p = 0.021). | [60] |
| **United Arab Emirates** | Physical | Sharp Injury | 1420 | Cross sectional / Cohort | 190 of the 1420 healthcare workers sustained a sharps injury and 53% of them were from blood contaminated sharps.  Healthcare workers were found to be at a 4.3 times greater risk of sharps injuries compared to support  workers (95% CI: 2.9–6.4, P < 0.001). | [85] |
|  |  | Noise | 177 | Cross sectional / Cohort | 21% of dentists experienced hearing issues after joining their clinics.  Dental technicians were particularly impacted, with 37% reporting tinnitus-like symptoms, compared to only 7% of administrative staff. | [83] |
|  |  | Noise | 468 | Cross sectional / Cohort | Around 89% were exposed to noise levels above the permissible 85 dB(A).  45% of the workers never used any type of hearing  protection equipment. | [82] |
|  |  | Physical Injury | 2573 | Cross sectional / Cohort | 149 patients (6%) suffered from falling object injuries, predominantly occurring at work (88.6%). The most injured areas were the upper extremity (38.3%) and lower extremity (37.6%), followed by  head and neck injuries (19.5%). | [81] |
|  |  | Radiation | 1011 | Cross sectional / Cohort | Highest mean annual effective dose was observed in cardiologists at 0.78 mSv, while radiographers,  nurses, radiologists, and physicians experienced lower doses (ranging from 0.42 to 0.45 mSv).  A significant difference in average annual effective doses was found among hospitals (F = 3.16, p <  0.05), particularly for cardiologists. | [79] |
|  |  | Physical Injury | 500 | Cross sectional / Cohort | 175 (35%) of the workers reported eye injury.  Workers with eye injuries generally had lower education levels (p < .0001), less safety training (p <  .0001), and less work experience (p < .0001). | [80] |
|  |  | Noise | 150 | Cross sectional / Cohort | Clinicians with tinnitus were exposed to higher noise levels, averaging 76.12 ± 5.90 dBA, significantly more than 69.25 ± 7.29 dBA for those without tinnitus (p < 0.001).  Of the 29 clinicians experiencing tinnitus, 18 had  intermittent and 11 had constant symptoms. | [84] |
|  | Chemical | General | 304 | Cross sectional / Cohort | Older workers faced a significantly higher rate of chronic bronchitis (p < 0.007), eye problems (p < 0.002), and fatigue (p < 0.004).  Smoking increased occurrence of chronic cough (p < 0.03) and chronic bronchitis (p < 0.01). | [90] |

|  |  | Pesticide | 532 | Case control | Erythrocyte acetylcholinesterase activity (AChE) among farm workers was lower compared to the control group, with foremen exhibitingsignificantly lower levels than farmers (p = 0.0001).  The hemoglobin-adjusted erythrocyte cholinesterase  activity (HAChE) was lower in farm workers than in controls. | [89] |
| --- | --- | --- | --- | --- | --- | --- |
|  |  | Dust / Fumes | 330 | Cross sectional / Cohort | Industrial workers demonstrated significantly higher blood lead levels than their non-industrial  counterparts (p < 0.001).  This elevated level in the industrial group was closely associated with a range of health issues, including nausea (p = 0.014), muscular problems (p = 0.004), fatigue (p = 0.016), irritability (p = 0.029), memory disturbances (p = 0.013). | [95] |
|  |  | Dust / Fumes | 323 | Cross sectional / Cohort | Almost all forced spirometric tests in the exposed garage workers were lower than in taxi drivers.  Prevlance of acute respiratory symptoms were higher in garage workers for throat (p < 0.02), hearing problems (p <0.002) and lower back pain (p  < 0.05) | [96] |
|  |  | Pesticide | 208 | Case control | Farm workers experienced instanced symptoms of diarrhea (p < 0.016), nausea/vomiting (p < 0.003), rashes (p < 0.002), eye irritation, anxiety (p < 0.003), headaches (p < 0.024), muscular symptoms (p <  0.015), memory loss (p < 0.0001), drowsiness, dizziness (p < 0.003), dyspnea (p < 0.005), | [93] |
|  |  | Pesticide | 196 | Cross sectional /  Cohort | A high use of chemical substances (70.9%) and pesticide mixtures (60.2%) was reported among the  farmers. | [97] |
|  |  | Heavy Metal(s) | 200 | Case control | Industrial workers exposed to lead had a significantly higher average blood lead level (77.5 ± 42.8 µg/dl) compared to 100 non-industrial workers (19.8 ± 12.3 µg/dl).  Essential amino acids and non-essential amino acids were observed to be significantly higher in industrial  workers (p < 0.0001). | [94] |
|  |  | Heavy Metal(s) | 220 | Cross sectional / Cohort | The exposed group showed elevated body mass index (BMI), and increases in both systolic and diastolic blood pressures were noted.  Correlations were significant between blood lead levels and key health indicators: systolic blood pressure (r = 0.286, p < 0.01), total cholesterol (r = 0.239, p < 0.01), triglycerides (r = 0.369, p < 0.01), and fasting glucose (r = 0.396, p < 0.01). | [87] |
|  |  | Heavy Metal(s) | 200 | Cross  sectional / Cohort | Industrial workers demonstrated significantly higher blood lead levels than their non-industrial  counterparts (p<0.001). | [86] |
|  |  | Dust / Fumes | 201 | Cross  sectional / Cohort | Higher percentage of the exposed group reported prolonged cough (30%), phlegm (25%), wheezing  (8%) and dyspnoea (21%). | [92] |
|  |  | Dust / Fumes | 227 | Cross sectional / Cohort | Dust concentration exceeded safe limits, reaching up to 15.20 mg/m^3 in some areas.  Exposed workers had a significantly higher  occurrence of cough (19.5%; OR=4.5; 95% CI=1.5– 13.2) and phlegm (14.8%; OR=13.3; 95% CI=1.8–  100.9) | [88] |
|  |  | Heavy Metal(s) | 75 | Case control | Welders exposed for over 8 hours a day had  significantly higher levels of cadmium (Cd) in hair and nickel (Ni) in nails (p < 0.05).  Workers with more than 20 years of experience exhibited significantly higher concentrations of Cd  and Ni in their hair (p < 0.05). | [91] |
|  | Biological | Infection | 21347 | Cross sectional / Cohort | 3.3% of 21,347 expatriate workers, primarily food handlers and housemaids, were infected with intestinal parasites.  Significant association between infection rates and the workers' nationalities was observed, with Indian workers showing the highest infection rates. | [98] |
|  | Ergonomics | Musculoskeletal Disorders | 100+ | Cross sectional / Cohort | Prevalence of musculoskeletal pain was highest in periodontists (83.3%), conservative dentists (80%),  and endodontists (77.8%).  Neck pain was reported by 39% of general dental practitioners. | [77] |

|  |  | Musculoskeletal Disorders | 60 | Cross sectional / Cohort | 83% of the dentists suffered from musculoskeletal pain (MSP).  The correlation between the regions of deviation and the regions of pain showed that the shoulder region  was more significant than other regions. | [76] |
| --- | --- | --- | --- | --- | --- | --- |
|  |  | Musculoskeletal Disorders | 202 | Cross sectional / Cohort | Musculoskeletal symptoms (MSP) in at least one body site were experienced by 48.5% of the participants in the past week and 68.3% over the past year.  There was no significant association between MSP prevalence and gender (OR 1.19, 95% CI 0.62–2.29, p  = 0.608). | [75] |
|  |  | Musculoskeletal Disorders | 179 | Cross sectional / Cohort | 90.4% of the dentists reported work-related  musculoskeletal disorders, mostly due to workplace stress.  Female dentists reported broader WMSD impacts compared to their male counterparts. | [78] |
|  |  | Various  Occupational Injuries | 844 | Cross sectional / Cohort | Musculoskeletal pain was reported by 68% (497 dentists), and 42% (306 dentists) experienced percutaneous injuries.  Contact dermatitis mainly from latex gloves affected  18% (135 dentists). | [99] |
|  | Miscellaneous | Brain Injury | 581 | Case series | Falls and falling objects were the leading causes of Traumatic Brain Injury (TBI), accounitng for (63%) and (34%) of the total incidents reported  respectively. | [100] |
| **Kingdom of Saudi Arabia** | Physical | Needlestick Injury | 107 | Cross sectional / Cohort | Nurses were most commonly affected by needlestick injuries (65%).  None of the affected staff developed bloodborne infection (HIV, hepatitis B) in the one year follow up  period. | [164] |
|  |  | Physical Injury | 193 | Cross sectional / Cohort | Incident rate of ocular injury was 111.8 per 1000 exposed.  More than one third of the injured were not wearing  eye protection. | [179] |
|  |  | Pulmonary | 149 | Cross sectional /  Cohort | There is a significant relationship between post-shift changes and exposure to cement dust. | [180] |
|  |  | Physical Injury | 65915 | Cross sectional / Cohort | The injury incidence rate was 7.1 per 1,000 full-time workers.  Falls were the main cause of injury (33.4%), followed by tools-related injuries (23.9%), falling objects  (14.5%) and car accidents (12.0%). | [181] |
|  |  | Noise | 368 | Cross sectional / Cohort | 75% (202 subjects) from the exposed group were exposed to a daily Leq above the permissible level of 85 dB(A) and most (61%) of these did not and had  never used any form of hearing protecion. | [182] |
|  |  | Needlestick Injury | 11457 | Cross sectional / Cohort | Housekeeping staff injuries ranked third at King Fahad National Guard Hospital.  Injuries caused by devices discarded inappropriately  commonly occurred at this facility. | [165] |
|  |  | Needlestick Injury | 148 | Cross sectional / Cohort | Needle recapping, careless disposal of sharps and blood extraction were responsible for over 50% of sharps injuries. | [166] |
|  |  | Noise | 20 | Cross sectional / Cohort | Textile, publishing and paper products industries are the most noisy industries.  Noise levels over 85 dBA occur at frequen- cies  mostly below 1 KHz across all industries. | [183] |
|  |  | Pulmonary | 505 | Cross sectional / Cohort | Dust level was determined by the gravimetric  method. Concentrations of personal respirable dust ranged from 2.13 mg/m3 in the kilns to 59.52  mg/m3 in the quarry area.  Cough and phlegm were found to be related to cigarette smoking, while wheezing, shortness of  breath, and bronchial asthma were related to dust  levels. | [187] |
|  |  | Needlestick Injury | 73 | Cross sectional / Cohort | Nurses were involved in 66% of instances of  needlestick injuries, physicians in 19%, technicians in 10%.  The majority (53.4%) of the injuries occurred after  use and before disposal of the objects. | [167] |
|  |  | Heat Stress | - | Cross sectional / Cohort | The WBGT in the service stations around planes are considerably higher than the recommended TLVs for non-acclimatized and acclimatized operators. | [184] |

|  |  | Needlestick Injury | 133 | Cross sectional / Cohort | Nurses (45%) sustained the highest number of injuries followed by physician (26%).  No seroconvention to HBV, HCV or HIV infection was  observed among followed-up healthcare workers. | [169] |
| --- | --- | --- | --- | --- | --- | --- |
|  |  | Needlestick Injury | 156 | Cross sectional / Cohort | Needlestick injury in the previous year was reported by 14.9% of physicians and 16.5% of nurses (0.21 and 0.38 injuries/person/year respectively). | [168] |
|  |  | Needlestick Injury | 477 | Cross sectional / Cohort | Nurses were the most affected by needlestick injuries.  Most of the incidents were caused by needles with disposable needle and hands were the most affected  body parts. | [170] |
|  |  | Noise | 200 | Cross sectional / Cohort | The average Leq (8h) at most of the aircraft maintenance operations was considerably high and most of the workers (89.5%) were exposed to noise  levels above or equal to 85 dBA. | [185] |
|  |  | Noise | 28 | Cross sectional / Cohort | Noise levels varied according to the type and size of a factory, and the type and number of machines used.  The highest noise levels were observed while manufacturing cans and forming steel reinforcement  for concrete, where noise levels exceed 90 dB(A). | [186] |
|  |  | Radiation | 34 | Cross sectional / Cohort | Cardiologists tend to receive higher doses than the nurses by a factor of 2–4 and can exceed 5 mSv per year.  No correlation exists between the eye doses of nurses and the cardiologists. | [152] |
|  |  | Noise | 76 | Cross sectional / Cohort | No significant difference was found between the two groups in the pure tone audiometry test.  A statistically significant difference was found in distortion product otoacoustic emissions (DPOAE) between the two groups in the left ear (p = 0.002),  and between the right and left ears (p = 0.005). | [188] |
|  |  | Sharp Injury | 326 | Cross sectional / Cohort | Nursing staff / students had the highest rate of exposure (149, or 45.6%), followed by physicians (57,  or 17.5%).  Surgeons were found to have a significantly higher risk for sharp injuries compared with other  physicians (p < 0.005). | [172] |
|  |  | Radiation | 4 | Cross sectional / Cohort | There is a significant association between the levels of radiation exposure in all selected hospitals  concerning imaging and waiting X-ray and CT-scan operating room (p < 0.01).  Most hospitals have lead aprons and thyroid shields  in place, but only about 50% have lead glasses and lead shields. | [153] |
|  |  | Physical Injury | 233 | Cross sectional / Cohort | The risk of ocular incidents as a result of fluid splashing was significantly higher in the absence of postgraduate qualification [odds ratio (OR) = 3.04, 95% confidence interval (95% CI): 1.70–5.43].  The most frequent postexpo- sure practice was to rinse eyes under running water (87.5%).  Approximately 4.2% and 9.2% of dental practitioners reported incidents of ocular injury and infection, respectively, and 14% reported that they never wore  any eye protection. | [189] |
|  |  | Radiation | 69 | Cross sectional / Cohort | The annual dose (also measured in units of μSv), based on annual records of doses, for medical physicists, technologists and nurses were 604, 680 and 1000 μSv respectively. | [155] |
|  |  | Needlestick Injury | 234 | Cross sectional / Cohort | Approximately 33% of the sample had an  experienced occupational infection while 24% had experienced a needlestick injury.  Occupational infection, needlestick injury and  recapping needles after use were associated with lack of training on biosafety (p < 0.05). | [171] |
|  |  | Radiation | 40 | Cross sectional / Cohort | Average and range values of 226Ra, 232Th, and 40K radioactivity concentrations were 83.7±0.3 (164.2-  35.2), 107.3±1.3 (199.9-48.5), and 192.0±1.1 (487.1-  24.6), Bq/kg respectively.  Measured values for 226Ra and 232Th were higher than the recommended levels. | [154] |

|  |  | Needlestick Injury | 450 | Cross sectional / Cohort | Lack of adequate knowledge of infection control procedures and disease transmission, non-  compliance with infection control protocol of  vaccination against hepatitis B virus, and attending  12 or less number of patients daily were significantly  associated with increased risk of NSIs (p ≤ 0.05). | [173] |
| --- | --- | --- | --- | --- | --- | --- |
|  |  | Heat Stress | 10 | Cross sectional / Cohort | Construction workers were exposed to excessive heat stress, indoors and outdoors over a large part of the work day.  Complying with a midday outdoor work ban (12–3  p.m.) was not effective in reducing heat stress risk. | [190] |
|  |  | Radiation | 5 | Cross sectional / Cohort | Pilots and aircrew are occupationally exposed to ionizing radiation, it is not possible in general, for pilots and aircrew to reach the occupational dose limit. | [156] |
|  |  | Heat Stress | 23 | Cross sectional / Cohort | The WBGT-OEL was exceeded frequently, on 63 person-days indoors (44%) and 91(78%) outdoors. Workload intensity was generally moderate, with  light intensity work more common outdoors. | [191] |
|  |  | Noise | 136 | Case control | Among the risk factors that may lead to  sensorineural hearing loss in dental staff, the age and the number of years in practice were found to be statistically significant factors. | [193] |
|  |  | Heat Stress | 65 | Cross sectional /  Cohort | A significant increase in albumin-creatinine ratio  (ACR) occurred among most workers over the study period; incidence of kidney injury (KI) was 18%. | [192] |
|  |  | Radiation | 30 | Cross sectional / Cohort | The respective mean annual dose and range for H  (10), H (0.07) and extremities were 3.05 ± 2.1 (0.23 -  0.91), 3.15 ± 2.2 (0.1 - 9.9) and 28.25 (0.1 - 298.0).  Extremity dose values were well below the annual dose limit (500 mSv). | [157] |
|  |  | Needlestick Injury | 609 | Cross sectional / Cohort | The overall needlestick and sharps injuries incidence rate was 24%.  Healthcare workers working in tertiary hospitals were 61% less likely to have needlestick and sharps  injuries than those employed in secondary hospitals. | [174] |
|  |  | Radiation | 206 | Cross sectional / Cohort | The mean annual occupational doses were 1.2 mSv, that for ambient doses outside of the isolation room corridors were found to be 0.2 mSv, while ambient doses at the nursing station were below the lower  limit of detection. | [160] |
|  |  | Noise | 409 | Cross sectional / Cohort | More than half of the participants (54.3%) were unaware of the consequences of noise exposure and none used proper hearing protection.  Pure-tone audiometry (PTA) showed hearing  impairment in 71.6% of the participants. | [194] |
|  |  | Sharp Injury | 131 | Cross sectional / Cohort | Females were more affected by sharps injury [98 out of 131 (74.8%)] than male [33 out of 131 (25.2%)].  The most affected HCWs categories were nurses  74(56.5%), doctor 23(17.6%) and housekeeping 18  (13.7%). | [175] |
|  |  | Radiation | 45152 | Cross sectional / Cohort | The annual mean effective doses were found to be  0.88 ± 0.002 mSv.  95% of the workers received a radiation exposure dose below 1.60 mSv. | [158] |
|  |  | Radiation | 2128 | Cross sectional / Cohort | The annual average effective dose was in the ranges of 0.44–1.04 mSv.  There was not enough evidence to conclude that dentists’ scores (M = 0.79, SD = 0.49) were any  different from dental nurses’ scores (M = 0.77, SD =  0.46), t (2126) = 0.58, (p = 0.55). | [159] |
|  |  | Various  Occupational Injuries | 73 | Cross sectional / Cohort | The most common cause of occupational injury was falling from height (87.7%).  Compared to migrants, Saudi nationals had a 45%  lower median hospital stay (p < 0.01). | [195] |
|  |  | Needlestick Injury | 361 | Cross sectional / Cohort | More than half of the injury events (53.8%) were not reported to the authority by the healthcare workers. Incidence of NSIs was highest among the physicians (36%) and was followed by nurses (34.8%), dentists  (29.2%), and medical technologists (21.1%). | [176] |
|  |  | Needlestick Injury | 219 | Cross sectional / Cohort | The prevalence of NSIs among nurses in Jeddah, Saudi Arabia, was 19.7%. | [177] |

|  |  | Radiation | 106 | Cross sectional / Cohort | The overall annual dose for staff was 1.4 ±0.37 (0.67– 8.74).  Annual radiation exposure in the four-radiology  department is below the annual dose limits. | [161] |
| --- | --- | --- | --- | --- | --- | --- |
|  |  | Needlestick Injury | 389 | Cross sectional / Cohort | Most common cause of NSIs was handling the sharp object (38.8%), followed by disposing of the sharp object (19.3%).  Nurses were found to be the highest at-risk category of HCWs experiencing NSIs (49.9%), while medical waste handlers (1.5%) and dentists (1.3%) were least likely to incur injuries. | [178] |
|  |  | Radiation | 4 | Cross sectional / Cohort | Occupational exposure was below the annual dose limits (20.0 mSv) for all practitioners, as well the  extremities dose was below the annual dose limits of 500 mSv. | [162] |
|  |  | Radiation | 20 | Cross  sectional / Cohort | Staff working in PET or PET-CT departments receive  significant doses from the radiopharmaceuticals and the energetic gamma rays from the CT scanners. | [163] |
|  | Chemical | Chemical exposure | 516 | Cross sectional / Cohort | Bronchial asthma, chronic bronchitis, and a  combined diagnosis were significantly higher among those exposed to high cumulative ammonia levels. Logistic regression analysis showed that ammonia concentration was significantly related to cough,  phlegm, shortness of breath with wheezing and bronchial asthma. | [196] |
|  |  | Chemical exposure | 432 | Cross sectional / Cohort | 30% of the air samples had ammonia concentrations that exceeded the threshold limit value. | [197] |
|  |  | Pesticide | 53 | Cross sectional / Cohort | There was a significant decrease in AChE activity (p < 0.001) ni pesticide workers (n = 43) relative to the  control group (n = 10).  No significant differences were detected ni hematological parameters, except for WBC count which was significantly higher (p < 0.01). | [198] |
|  |  | Heavy Metal(s) | 49 | Cross sectional / Cohort | Blood carboxyhemoglobin and serum ferritin were statistically increased in Jeddah firefighters, (p < 0.05 and p < 0.05 respectively). | [199] |
|  |  | Dust / Fumes | 163 | Cross sectional /  Cohort | Out of the 8 factories samples, welders in factories 1, 2, 3, and 4 were exposed to welding fume  concentration above the SASO limit value | [200] |
|  |  | Chemical exposure | 56 | Cross sectional / Cohort | Only 10.7% of participants who were exposed to cleaning chemicals for more than 10 years had FEV1 and FVC less than their longitudinal normal limits at the end of the study.  Smoking and gender were associated with statistically significant decreases in some of the  spirometric parameters. | [201] |
|  |  | Heavy Metal(s) | 150 | Cross sectional / Cohort | No statistically significant differences in the levels of blood heavy metals in firefighters were noted as  compared to normal controls.  Results indicate that fire smoke mainly affects serum biochemical and hematological parameters but does not affect serum heavy metals levels. | [202] |
|  |  | Dust / Fumes | 44 | Cross sectional / Cohort | 15 subjects out of 44 showed acute inflammation when cytological analysis was conducted.  Acute cough and astham was noted in 4 and 5 of the total subjects respectively. | [204] |
|  |  | Chemical exposure | 278 | Cross sectional / Cohort | In plastic industry workers, the prevalence of pre- diabetes was 176 (63.30%) and T2DM was 66  (23.74%); however, 36 (12.95%) plastic plant workers were normal.  The prevalence of pre-diabetes and T2DM among plastic industry workers was significantly increased with duration of working exposure in plastic industry  (p = 0.0001). | [203] |
|  |  | Chemical exposure | 131 | Cross sectional / Cohort | At low exposure levels, SPMA and oxidation  biomarker levels were correlated among them and were associated with the smoking habit.  High levels of benzene exposure can cause an accumulation of SPMA and bring about the  formation of the oxidation biomarkers studied to  saturation. | [205] |

|  |  | Heavy Metal(s) | 127 | Cross sectional / Cohort | The low Blood Lead Level (BLL) group had a mean level of 3.94 μg/dL, while the high BLL group had levels of 15.33 μg/dL (P < 0.001). | [206] |
| --- | --- | --- | --- | --- | --- | --- |
|  |  | Pulmonary | 160 | Cross sectional / Cohort | 44 employees (28%) reported at least one respiratory symptom.  Shortness of breath was the most common symptom reported by 71 (44%) followed by wheezing in 40  (25%). | [207] |
|  |  | Pulmonary | 50 | Cross sectional / Cohort | 5 (10%) participants showed productive cough, 7  (14%) were having complication of asthma, 7 (14%) were having lungs infection. | [208] |
|  |  | Dust / Fumes | 186 | Cross sectional / Cohort | Among the cement mill personnel, 79 (42.47%) were non-diabetics, 28 (15.05%) were pre-diabetics, and  79 (42.47%) were diabetics.  The prevalence of pre-diabetes and T2DM among cement mill employees was considerably associated with the period of employment in the cement industry (p = 0.032). | [209] |
|  |  | Chemical exposure | 312 | Cross sectional / Cohort | The highest reported respiratory symptom was chest tightness and whistling among exposed (22.7%) and unexposed (10%).  The reported use of airway protection (masks) was very low. | [211] |
|  |  | Chemical exposure | 1609 | Cross sectional / Cohort | In wood industry workers, the prevalence of prediabetes (PD) was 64 (40.50%) and in type 2 diabetes mellitus (T2DM), it was 21 (13.29%) among welding workers.  The prevalence of prediabetes and T2DM among workers increased with the duration of working exposure in the wood, welding, motor mechanic,  and oil refinery industries. | [210] |
|  |  | Chemical exposure | 200 | Cross sectional / Cohort | The self-reported prevalence of eczema/contact dermatitis within the past year was 14.5% (n=29), with hand lesions being most common (63%).  Most of them wore some or the other safety equipment like safety shoes (20.9%), gloves (20.3%), face masks (20%), earmuffs (10%), and helmets  (2.8%). | [212] |
|  |  | Dust / Fumes | 125 | Cross sectional / Cohort | The overall prevalence of wheezing or whistling was 16.8%, with a higher prevalence in exposed group  35.5%. | [213] |
|  |  | Chemical exposure | 105 | Cross sectional / Cohort | Mean levels of malondialdehyde (MDA), glutathione S-transferase (GST), and lactate dehydrogenase (LDH) were significantly elevated in exposed workers  compared to the control group (p < 0.001). | [215] |
|  |  | Dust / Fumes | 265 | Cross sectional / Cohort | In incense sellers, the prevalence of pre-diabeties was 125 (47.2%) and diabetes was 75 (28.3%).  There was an increase in HbA1c levels as work  exposure in incense shops increased. | [214] |
|  | Biological | Infection | 1194 | Cross sectional / Cohort | The prevalence of warts (Papilloma virus) was found to be 28.5 % in slaughterhouses, higher than the general population.  The factors present in the work environment that showed a significant correlation (a <= 0.05) with the presence of warts were the following: steel-mesh gloves that were too large for the wearer, work with a saw, handling of cold objects, and high humidity  levels. | [216] |
|  |  | Infection | 352 | Cross sectional / Cohort | Of the total 352 samples, S. aureus was isolated from 112 (31.8%) specimens.  The rate of S. aureus in the medical staff, nurses, cleaners and administrative personnel were 35.5% and 2.6%, respectively. | [217] |
|  |  | Infection | 106 | Cross sectional / Cohort | The majority of episodes were recorded among the nursing staff (n/N = 87/106; 82%) followed by technicians (n/N = 10/106; 9.4%). | [218] |
|  |  | Infection | 298 | Cross sectional / Cohort | Exposed HCWs tended to be female (62.9%), non-  Saudi (83.9%), nurses (68.6%), or respiratory  therapists (24.0%) working in critical care locations (72.8%).  Only 48.9% (115/235) of exposed healthcare workers  who had negative or unknown preexposure | [219] |

|  |  |  |  |  | tuberculin skin test TST status had their TST tested after exposure. |  |
| --- | --- | --- | --- | --- | --- | --- |
|  |  | Infection | 80 | Cross sectional / Cohort | All workers tested negative for Crimean–Congo haemorrhagic fever (CCHF) and Rift Valley Fever  (RVF), one was positive for Alkhurma haemorrhagic fever (AHF) and six for Brucella.  Most of the butchers 98.75% (n = 79) never used eye protection, few occupational workers dressed in  personal protective clothing or footwear. | [221] |
|  |  | Infection | 211 | Cross sectional / Cohort | Symptoms were observed only in HCWs from the Mecca cohort, with 29.3% experiencing respiratory symptoms during the Hajj period or in the subsequent days and 3.3% having gastrointestinal symptoms.  Acquisition rates of at least 1 respiratory virus were 14.7% in the Mecca cohort and 3.4% in the Al-Ahsa  cohort (p = 0.003). | [220] |
|  | Ergonomics | Musculoskeletal Disorders | 204 | Cross sectional / Cohort | 111 (54.4%) of the subjects complained of neck pain and 150 (73.5%) complained of back pain.  Only 37% of those complaining of back pain sought medical help. | [101] |
|  |  | Musculoskeletal Disorders | 389 | Cross sectional / Cohort | A high number of construction workers experienced neck pain 29 (7.5%), shoulder pain 41(10.5%), upper  back pain 24(6.2%), lower back pain 64 (16.5%), legs  pain 93 (23.9%), feet pain 52 (13.4%), head  heaviness 44 (11.3%) and whole body fatigue 78  (20.1%).  These complaints were significantly associated with long-term duration-response in building construction industry (p < 0.05). | [102] |
|  |  | Musculoskeletal Disorders | 964 | Cross sectional / Cohort | Three significant predictors for the presence of back pain were identified: female gender (odds ratio [OR]  = 1.9, 95% confidence interval [CI] = 1.3–2.7), Saudi nationality (OR = 2.3, 95% CI = 1.4–3.9), and working as a surgeon (OR = 5.4, 95% CI = 1.4–21.5).  Saudi females being at particularly high risk of  reporting back pain (OR = 3.9, 95% CI = 1.8–8.5). | [103] |
|  |  | Musculoskeletal Disorders | 200 | Cross sectional / Cohort | 85% of the nurses reported experiencing at least one musculoskeletal symptom.  Prolonged working hours and being underweight were significantly associated with the development  of lower back and ankles/feet pain ( p = 0.018 and p  = 0.004, respectively). | [104] |
|  |  | Musculoskeletal Disorders | 165 | Cross sectional / Cohort | 80 (48.5%) of the responding workers had pain in neck, shoulders, lower back, hand, knee, or ankle.  There was an association between years of  experience, duration of break during work, and use of protective equipment with the prevalence of musculoskeletal pain in construction workers (p < 0.05). | [105] |
|  |  | Musculoskeletal Disorders | 80 | Cross sectional / Cohort | The prevalence of MSDs among respondents was 77.9% (n=53) with the most commonly affected  areas the lower back (73.5%) (39/53) followed by the neck (66%) (35/53) and the shoulders (43.3%)  (23/53). | [106] |
|  |  | Musculoskeletal Disorders | 165 | Cross sectional / Cohort | 70% (113) eye care professionals reported neck and back pain.  Pain appeared to be associated with reported  physical discomfort during professional activities (p < 0.01) but not with mental stress. | [107] |
|  |  | Musculoskeletal Disorders | 105 | Cross sectional / Cohort | 31 (30.09%) of 105 cabin crew staff of Saudi Airlines had neck pain. | [108] |
|  |  | Musculoskeletal Disorders | 120 | Cross sectional / Cohort | No statistically significant associations were detected between lower back pain and any of the general risk factors (p > 0.05).  most of the operating room risky activities were significantly associated with the occurrence of LBP (p  <0.05) | [109] |
|  |  | Musculoskeletal Disorders | 690 | Cross sectional / Cohort | Working hours/week and work-related musculoskeletal disorders (WRMDs) were significantly correlated (p = 0.005).  Lower back (46.5%) and neck (26.6%) pain were most commonly encountered by physical therapists. | [110] |
|  |  | Musculoskeletal Disorders | 224 | Cross sectional / Cohort | 184 (90.2%) respondents reported having  musculoskeletal pain, with lower back pain being most commonly reported musculoskeletal pain  (68.1%).  Older age was associated with lower back pain (OR 1.23; 95% CI 1.00 to 1.50) and women had double the risk of shoulder pain (OR 2.52; 95% CI 1.12 to 5.68). | [111] |
|  |  | Musculoskeletal Disorders | 515 | Cross sectional / Cohort | Pain in lower back (64%) and neck (60%) were the most common prevalent musculoskeletal symptoms (MS).  Female dentists were at more risk of MS than the  male dentists (OR 1.63, 95%CI 1.02-2.59). | [113] |
|  |  | Musculoskeletal Disorders | 279 | Cross sectional / Cohort | The prevalence of carpal tunnel syndrome (CTS) among the laboratory technicians was 9.7% (27 / 279). | [114] |
|  |  | Musculoskeletal Disorders | 600 | Cross sectional / Cohort | 89.65% of the therapists reported lower back pain (LBP) after beginning their PT practice.  The prevalence of work-related LBP among physical therapist in Riyadh was high, affecting patient care and daily activities of the therapists. | [115] |
|  |  | Musculoskeletal Disorders | 100 | Cross sectional / Cohort | 84% of respondents suffer from pain they associated with their ultrasound practice.  The shoulder, back, neck and right hand were the  main symptomatic body areas. | [116] |
|  |  | Musculoskeletal Disorders | 515 | Cross sectional /  Cohort | Working in the dental colleges’ clinics was associated with increased risk of musculoskeletal symptoms  (adjusted OR 2.30, 95% CI [1.27, 4.18]. | [112] |
|  |  | Musculoskeletal Disorders | 124 | Cross sectional /  Cohort | The most commonly reported MSD was back pain  (26.6%) followed by neck pain (21.0%) and shoulder pain (12.1%). | [117] |
|  |  | Musculoskeletal Disorders | 102 | Cross sectional / Cohort | The annual prevalence of low back pain was 44.1%, and it was more common among Saudi workers  (67.9%) compared with non-Saudi workers (35.1%). Spearman rank correlation showed correlation between the symptom pain scale and smoking (rs = 0.259; P = 0.008), and exercise was associated with  lower pain scale scores (rs = −0.241, P = 0.015). | [118] |
|  |  | Musculoskeletal Disorders | 740 | Cross sectional / Cohort | The prevalence of lower back pain (LBP)  necessitating medications and or physiotherapy was 40.5%, while the prevalence of LBP requiring medical consultation was 20%. | [119] |
|  |  | Musculoskeletal Disorders | 263 | Cross sectional / Cohort | Multivariate logistic regression analysis revealed being a female radiologist (OR=2.7; 95% CI: 1.2–6.5), aged 30–39years (OR=4.1; 95% CI: 1.1–15.3), and  predominantly reviewing computed tomography (CT) images (OR = 4.1; 95% CI: 1.4–12.3) or ultrasound  scans (OR = 5.9; 95% CI: 1.4–25.3) were associated  with higher prevalence of disabling musculoskeletal symptoms. | [124] |
|  |  | Musculoskeletal Disorders | 60 | Cross sectional / Cohort | Incidence of lower back pain among nurses was 61.7%.  66.7% of the nurses experienced lower back pain only after taking on a nursing job. | [122] |
|  |  | Musculoskeletal Disorders | 196 | Cross sectional / Cohort | Back and neck areas represented the most affected body parts (71.1% and 59.8%, respectively).  A significantly higher percentage of those with musculoskeletal manifestations practiced for 5-10  years (28.9%) (p = 0.006). | [120] |
|  |  | Musculoskeletal Disorders | 223 | Cross sectional / Cohort | Female dentists had a significantly greater risk of having CTS symptoms than male den- tists (OR 2.13; 95% CI 1.09–4.17).  There was no relationship between CTS symptoms and age, marital status, history of smoking, exercise, dental specialty, occupation period, or having other  educational degrees. | [121] |
|  |  | Musculoskeletal Disorders | 254 | Cross sectional / Cohort | The prevalence of musculoskeletal pain among  secondary school female teachers in Aljouf region, Saudi Arabia was 68.50%.  The main sites of musculoskeletal pain were lower back (46.5%) and neck (26.6%) pain were most commonly encountered by physical therapists. | [123] |
|  |  | Musculoskeletal Disorders | 322 | Cross sectional / Cohort | Among the respondents, only 4.82% could correctly explain ergonomics and 14.16% had attended a course or workshop on ergonomics. Females were significantly more aware of work-related musculoskeletal disorders (WRMSDs) than males. | [125] |
|  |  | Musculoskeletal Disorders | 306 | Cross sectional / Cohort | The overall prevalence of WMSDs in any body region was 82% in the last 12 months and it was mainly linked to lower back pain (61%). In the logistic regression, age <30 years, working in specific laboratories and working in hematology/flow-cytometry laboratory divisions were all found to be significant risk factors (p < 0.05). | [126] |
|  |  | Musculoskeletal Disorders | 391 | Cross sectional / Cohort | 42% of the teachers were found to have varicose veins most of which were females. Participants who did regular exercises were less prone to varicose than irregularly exercising participants (P = 0.0001). | [127] |
|  |  | Musculoskeletal Disorders | 94 | Cross sectional / Cohort | Most of them (63.8%) indicated that they experienced discomfort during the last 12 months in their lower back, followed by shoulders (50%), and upper back (48.9%). | [128] |
|  |  | Musculoskeletal Disorders | 1242 | Cross sectional / Cohort | The most common symptom noted in the study group was tingling and numbness of fingers (66.46%) followed by neck pain (66.34%). | [130] |
|  |  | Musculoskeletal Disorders | 207 | Cross sectional / Cohort | The overall prevalence rate was 42.5%, lower back was the most affected body part (31.9%) and hip/thigh region was the least common (2.4%). Musculoskeletal disorders (MSDs) were lower among the higher academic occupations. | [129] |
|  |  | Musculoskeletal Disorders | 323 | Cross sectional / Cohort | Prevalence of lower back pain (LBP) was 65.6% (n=212). Age and the type of ward were found significantly associated with LBP (p value = 0.007) & (p-value = 0.02), respectively. | [131] |
|  |  | Musculoskeletal Disorders | 259 | Cross sectional / Cohort | Rehabilitation professionals experienced 73.7% 1-year lower back pain prevalence. Lower back pain was responsible for stopping work in 32.5% of participants, with a mean 1.38 (±2.96) days off work during the last year. | [132] |
|  |  | Musculoskeletal Disorders | 251 | Cross sectional / Cohort | The overall prevalence of Musculoskeletal disorders (MSDs) was 87.3%. Female teachers (95.3%) suffered more than their male counterparts (81.4%). Smoking was predicted to be a significant risk factor for lower back disorders (p < 0.05). | [133] |
|  |  | Musculoskeletal Disorders | 503 | Cross sectional / Cohort | The proportion of musculoskeletal pain was 91%, and the most common site of pain was back (74.4%), followed by the shoulder (57.5%) and leg (51.5%). | [134] |
|  |  | Musculoskeletal Disorders | 462 | Cross sectional / Cohort | The prevalence of Work‐related musculoskeletal disorders (WMSD) was 87% (95% CL; 83.9% to 90.3%) among the respondents. Gender, study year, type of practice, having clinics for left‐handed, hours of clinical practice, sitting in the proper position while working, use of coping strategies were significantly associated with WMSD prevalence (p < 0.05). Males were OR = 10 times at higher risk of WMSD compared to females (p = 0.0001). | [151] |
|  |  | Musculoskeletal Disorders | 234 | Cross sectional / Cohort | Cumulative prevalence of LBP was 82.9%, annual prevalence was 85.5%, while one-week prevalence of LBP was 53.6%. The factor significantly associated with LBP over the past 12 months was manual lifting of patients (p = 0.030). | [136] |
|  |  | Musculoskeletal Disorders | 113 | Cross sectional / Cohort | The reported 12-month incidence of work-related musculoskeletal disorders (WMSDs) was 83.8%.  The low back (63.7%) was the most common site of these disorders, followed by the neck (59.2%), while the hip/thigh (4.4%) was the least involved body part. | [137] |
|  |  | Musculoskeletal Disorders | 381 | Cross sectional / Cohort | Musculoskeletal symptoms (MSS) were indicated by 326 responders (85.6%). An association between work-related MSS and gender was observed (p = 0.021). | [142] |
|  |  | Musculoskeletal Disorders | 130 | Cross sectional / Cohort | The results revealed that there was a high incidence of MSDs in the neck, shoulder, and lower back painamong dental personnel (72.6%). | [143] |
|  |  | Musculoskeletal Disorders | 451 | Cross sectional / Cohort | The prevalence of musculoskeletal symptoms in any region during the past 12 months preceding the study was 84.5%, and only 30% have sought medical advice. The most common area of complaint was the lower back (54.5%). Rapid Office Strain Assessment (ROSA) score was an independent risk factor (OR = 1.77, 95% CI = 1.05–2.96). | [138] |
|  |  | Musculoskeletal Disorders | 90 | Cross sectional / Cohort | The prevalence of musculoskeletal pain among faculty members was 77.8%, and the most common site of musculoskeletal pain occurred at two different sites of the three (low back, neck, and shoulder), with a prevalence of 38.9%. As for risk factors of musculoskeletal pain, only age group showed a significant correlation with the site of musculoskeletal pain (p = 0.024) | [135] |
|  |  | Musculoskeletal Disorders | 416 | Cross sectional / Cohort | The prevalence of computer vision syndrome (CVS) was 65.4% (95% CI: 60.8-70.0). Female sex (p < 0.001), work as a general radiologist (p = 0.05), and the use of eyeglasses (p = 0.001) were significant predictors of CVS. | [139] |
|  |  | Musculoskeletal Disorders | 413 | Cross sectional / Cohort | The prevalence of neck pain in participants was 64% during a twelve-month period. Reduced working hours were associated with a reduction in the likelihood of having neck pain (OR = 0.42, 95%CI [0.33,0.53]. | [140] |
|  |  | Musculoskeletal Disorders | 251 | Cross sectional / Cohort | The prevalence of MSDs in the last 12 months was 93.63% (235 of 251 teachers). The most affected site was the lower back (183 of 251 teachers, 72.91%), followed by the shoulders (168 of 251 teachers, 66.93%), and the least affected sites were the elbows (45 of 251 teachers, 17.93%). | [141] |
|  |  | Musculoskeletal Disorders | 202 | Cross sectional / Cohort | 62 (32%) reported that they have developed some type of musculoskeletal pain after joining their profession and 38 (61%) respondents further reported that it was so severe that they had to seek some sort of treatment for their pain. | [146] |
|  |  | Musculoskeletal Disorders | 404 | Cross sectional / Cohort | Back pain, shoulder pain, and knee pain were the most reported symptoms of WMSDs in the past 12 months, while elbow pain was the least experienced. Regression analysis revealed a relationship between moderate to severe pain and a pain duration of more than 12 months (p=0.001). | [145] |
|  |  | Musculoskeletal Disorders | 482 | Cross sectional / Cohort | The prevalence of varicose veins among the nurses of both hospitals was 76 (15.8%). Some of the main risk factors associated with varicose veins were ethnicity (p = 0.007), carrying heavy items (p = 0.001), lack of exercise (p = 0.031). | [144] |
|  |  | Musculoskeletal Disorders | 380 | Cross sectional / Cohort | A positive history of past back issues was found in 48.2 % of the participants. This study demonstrated a high prevalence of back disability among male teachers in Makkah, Saudi Arabia. | [147] |
|  |  | Musculoskeletal Disorders | 308 | Cross sectional / Cohort | Work-related musculoskeletal pain (WRMSP) was more prevalent among cardiac sonographers than controls(84.8% vs 64.7%, p < 0.0001). | [148] |
|  |  | Musculoskeletal Disorders | 208 | Cross sectional / Cohort | 38% of surgeons were found to have back pain.  The highest prevalence was found among general surgeons, followed by gynecologists. | [149] |
|  |  | Musculoskeletal Disorders | 814 | Cross sectional / Cohort | The prevalence of musculoskeletal symptoms (MSS) among the radiologists was 87.7%. The most common symptoms were neck pain (59.3%) and lower back pain (57.1%). | [150] |

**References:**

[20] Tantawy SA, Halwachi B, Ghanim A, Mansoori A, Kamel DM. Work-related musculoskeletal disorders among employees with different tasks: an Ahlia University case study. Physiotherapy Quarterly. 2022;30[4]:59-64. doi:10.5114/pq.2022.121146
[21] Nasaif H, Alaradi M, Hammam RAA, Bucheeri M, Abdulla M, Abdulla H. Prevalence of self-reported musculoskeletal symptoms among nurses: a multicenter cross-sectional study in Bahrain. International Journal of Occupational Safety and Ergonomics. 2022;29[1]:192-198. doi:10.1080/10803548.2021.2025315
[22] Matlab M, Cowman S, Al-Shagag A. Needle Stick Injuries and Compliance among Doctors and Nurses. Bahrain Medical Bulletin. 2017;39[4]:225-228. doi:10.12816/0047772
[23] Almoosa A, Asal A, Atif M. Occupational Eye Injury : The neglected role of eye protection. Bahrain Medical Bulletin. 2017;39[2]:82-84. doi:10.12816/0047525
[24] Alsayyad AS, Hamadeh RR. The Burden of climate-related conditions among laborers at Al-Razi Health Centre, Bahrain. Journal of the Bahrain Medical Society. 2014;25[1]. doi:10.26715/jbms.p25_2
[25] Madani IM, Khalfan S, Khalfan HA, Jidah J, Aladin MN. Occupational exposure to carbon monoxide during charcoal meat grilling. Science of the Total Environment. 1992;114:141-147. doi:10.1016/0048-9697[92]90420-w
[26] Al-Nooh AA, Alajmi AAA, Wood D. The Prevalence of Cardiovascular Disease Risk Factors among Employees in the Kingdom of Bahrain between October 2010 and March 2011: A Cross-Sectional Study from a Workplace Health Campaign. Cardiology Research and Practice. 2014;2014:1-9. doi:10.1155/2014/832421
[27] Shikdar AA. Identification of ergonomic issues that affect workers in oilrigs in desert environments. International Journal of Occupational Safety and Ergonomics. 2004;10[2]:169-177. doi:10.1080/10803548.2004.11076605
[28] Shikdar AA, Al-Kindi M. Office Ergonomics: Deficiencies in computer Workstation design. International Journal of Occupational Safety and Ergonomics. 2007;13[2]:215-223. doi:10.1080/10803548.2007.11076722
[29] Palathoti S, Aghbari AHMA, Otitolaiye VO. Effect of long extended working hours on the occupational health and safety of oil and gas workers in the Sultanate of Oman. International Journal of Occupational Safety and Health. 2023;13[4]:419-428. doi:10.3126/ijosh.v13i4.48968
[30] Shikdar AA, Khadem M, Al-Harthy S. An ergonomics intervention study of reducing health complaints among office employees. 2008 IEEE International Conference on Industrial Engineering and Engineering Management. Published online December 1, 2008. doi:10.1109/ieem.2008.4738215
[31] Dhar D. Retrospective study of injuries in military parachuting. Medical Journal Armed Forces India. 2007;63[4]:353-355. doi:10.1016/s0377-1237[07]80014-1
[32] Manjunath GT, Aravindhakshan R, Varghese S. Effect of fasting during Ramadan on thermal stress parameters. Eastern Mediterranean Health Journal. 2019;25[1]:34-39. doi:10.26719/emhj.18.013
[33] Al-Rubaee FR, Al-Maniri A. Work Related Injuries in an Oil field in Oman. Oman Medical Journal. 2011;26[5]:315-318. doi:10.5001/omj.2011.79
[34] Al-Aamria M, Al-Balushia N, Bailey DL. Estimation of radiation exposure to workers during [18F] FDG PET/CT procedures at Molecular Imaging Center, Oman. Journal of Medical Imaging and Radiation Sciences. 2019;50[4]:565-570. doi:10.1016/j.jmir.2019.05.009
[35] Bouchareb Y, Al-Maimani N, Al-Maskery I, et al. ASSESSMENT OF OCCUPATIONAL RADIATION DOSES IN DIFFERENT DIAGNOSTIC, INTERVENTIONAL AND THERAPEUTIC RADIOLOGY AND MOLECULAR IMAGING SERVICES IN OMAN. Radiation Protection Dosimetry. 2021;197[1]:36-45. doi:10.1093/rpd/ncab152
[36] Bouchareb Y, Al-Mabsali J, Al-Zeheimi H, Al-Jabri A, Tag N, Al-Dhuhli H. Evaluation of institutional whole-body and extremity occupational radiation doses in nuclear medicine. Radiation Protection Dosimetry. 2023;199[19]:2318-2327. doi:10.1093/rpd/ncad235
[37] Al-Harthy NA, Abugad H, Zabeeri N, Alghamdi A, Yousif GFA, Darwish MA. Noise Mapping, Prevalence and Risk Factors of Noise-Induced Hearing Loss among Workers at Muscat International Airport. International Journal of Environmental Research and Public Health. 2022;19[13]:7952. doi:10.3390/ijerph19137952
[38] Ramakrishnan, H. K., Pallavi, N., & Doolgindachbaporn, T. Occupational Health Hazard Identification and Risk Mitigation at Engineering Procurement and Construction Projects: Sultanate of Oman. EnvironmentAsia. 2022; 15[Special issue]: 56–67. https://doi.org/10.14456/ea.2022.22
[39] Esechie JO, Ibitayo OO. Pesticide use and related health problems among greenhouse workers in Batinah Coastal Region of Oman. Journal of Forensic and Legal Medicine. 2011;18[5]:198-203. doi:10.1016/j.jflm.2011.02.009
[40] Williams RJ, Al-Busaidy S, Mehta F, et al. Crimean‐Congo haemorrhagic fever: a seroepidemiological and tick survey in the Sultanate of Oman. Tropical Medicine & International Health. 2000;5[2]:99-106. doi:10.1046/j.1365-3156.2000.00524.x
[41] Almayahi ZK. Failure of doxycycline postexposure prophylaxis in human brucellosis contracted through needlestick injury. Infectious Diseases in Clinical Practice. 2019;27[5]:301-302. doi:10.1097/ipc.0000000000000768
[42] Ellingsen T, Bener A, Gehani AA. Study of shift work and risk of coronary events. Journal of the Royal Society for the Promotion of Health. 2007;127[6]:265-267. doi:10.1177/1466424007083702
[43] Abolfotouh SM, Mahmoud KM, Faraj K, Moammer G, ElSayed A, Abolfotouh MA. Prevalence, consequences and predictors of low back pain among nurses in a tertiary care setting. International Orthopaedics. 2015;39[12]:2439-2449. doi:10.1007/s00264-015-2900-x
[44] Hanna F, Daas RN, El-Shareif TJ, Al-Marridi HH, Al-Rojoub ZM, Adegboye OA. The relationship between sedentary behavior, back pain, and psychosocial correlates among university employees. Frontiers in Public Health. 2019;7. doi:10.3389/fpubh.2019.00080
[45] Shah SFUH, Bener A, Al‐Kaabi S, Khal ALA, Samson S. The epidemiology of needle stick injuries among health care workers in a newly developed country. Safety Science. 2006;44[5]:387-394. doi:10.1016/j.ssci.2005.11.002
[46] Bener A, Rahman YSA, Aleem EYA, Khalid MK. Trends and Characteristics of Head and Neck Injury from Falls: A hospital based study, Qatar. PubMed. Published online May 1, 2011. https://pubmed.ncbi.nlm.nih.gov/21969897
[47] Bener A, Rahman YSA, Aleem EYA, Khalid MK. Trends and characteristics of injuries in the State of Qatar: hospital-based study. International Journal of Injury Control and Safety Promotion. 2012;19[4]:368-372. doi:10.1080/17457300.2012.656314
[48] Pradhan B, Kjellström T, Atar D, et al. Heat stress impacts on cardiac mortality in Nepali migrant workers in Qatar. Cardiology. 2019;143[1-2]:37-48. doi:10.1159/000500853
[49] Razzakh SS, Qureshi MFH. Needlestick injuries among healthcare personnel in Qatar: A retrospective study. Qatar Medical Journal. 2021;2021[2]. doi:10.5339/qmj.2021.35
[50] Marji T, Syed MA. Primary care dental professionals’ experiences of sharp injuries in Qatar: A cross-sectional study. Frontiers in Oral Health. 2022;3. doi:10.3389/froh.2022.1014004
[51] Consunji R, El‐Menyar A, Hirani N, et al. Work-related injuries in Qatar for 1 year: an initial report from the work-related injury unified registry for Qatar. Qatar Medical Journal. 2022;2022[4]. doi:10.5339/qmj.2022.58
[52] Shomar B, Al‐Saad K, Nriagu JO. Mishandling and exposure of farm workers in Qatar to organophosphate pesticides. Environmental Research. 2014;133:312-320. doi:10.1016/j.envres.2014.06.010
[53] Mochtar IA, Rw H. Assessment of the 10-year risk of coronary heart disease events for Qatar Petroleum’s firefighters and non-firefighter staff in Qatar. Eastern Mediterranean Health Journal. 2012;18[2]:127-131. doi:10.26719/2012.18.2.127
[54] Al‐Thani H, El‐Menyar A, Consunji R, et al. Epidemiology of occupational injuries by nationality in Qatar: Evidence for focused occupational safety programmes. Injury-International Journal of the Care of the Injured. 2015;46[9]:1806-1813. doi:10.1016/j.injury.2015.04.023
[55] Shehab D, Al-Jarallah K, Moussa M, Adham N. Prevalence of Low Back Pain among Physical Therapists in Kuwait. Medical Principles and Practice. 2003;12[4]:224-230. doi:10.1159/000072288
[56] Landry MD, Raman SR, Sulway C, Golightly YM, Hamdan E. Prevalence and risk factors associated with low back pain among health care providers in a Kuwait hospital. Spine. 2008;33[5]:539-545. doi:10.1097/brs.0b013e3181657df7
[57] Alrowayeh HN, Alshatti TA, Aljadi SH, Fares M, Alshamire MM, Alwazan SS. Prevalence, characteristics, and impacts of work-related musculoskeletal disorders: a survey among physical therapists in the State of Kuwait. BMC Musculoskeletal Disorders. 2010;11[1]. doi:10.1186/1471-2474-11-116
[58] Raman SR, Al-Halabi B, Hamdan E, Landry MD. Prevalence and risk factors associated with self-reported carpal tunnel syndrome [CTS] among office workers in Kuwait. BMC Research Notes. 2012;5[1]. doi:10.1186/1756-0500-5-289
[59] Alnaser MZ, Aljadi SH. Physical therapists with work-related musculoskeletal disorders in the State of Kuwait: A comparison across countries and health care professions. Work-a Journal of Prevention Assessment & Rehabilitation. 2019;63[2]:261-268. doi:10.3233/wor-192927
[60] Alnaser MZ, Almaqsied AM, Alshatti SA. Risk factors for work-related musculoskeletal disorders of dentists in Kuwait and the impact on health and economic status. Work-a Journal of Prevention Assessment & Rehabilitation. 2021;68[1]:213-221. doi:10.3233/wor-203369
[61] Fm EA. Assessment of radium-226 in two cases of radium luminizers using whole-body counting. Health Physics. 1982;43[4]:567-572. doi:10.1097/00004032-198210000-00010
[62] Mustafa A, Sabol J, Janeczek J. Doses from Occupational Exposure. Health Physics. 1985;49[6]:1197-1204. doi:10.1097/00004032-198512000-00016
[63] Al-Abdulsalam A, Brindhaban A. Occupational Radiation Exposure among the Staff of Departments of Nuclear Medicine and Diagnostic Radiology in Kuwait. Medical Principles and Practice. 2013;23[2]:129-133. doi:10.1159/000357123
[64] Omar A, Abdo NM, Salama MF, Al-Mousa HH. Occupational Injuries Prone to Infectious Risks amongst Healthcare Personnel in Kuwait: A Retrospective Study. Medical Principles and Practice. 2014;24[2]:123-128. doi:10.1159/000369462
[65] Alnaaimi M, Alkhorayef M, Omar MM, et al. Occupational radiation exposure in nuclear medicine department in Kuwait. Radiation Physics and Chemistry. 2017;140:233-236. doi:10.1016/j.radphyschem.2017.02.048
[66] Alnaaimi M, Alduaij M, Shenawy F, et al. Assessment of eye doses to staff involved in interventional cardiology procedures in Kuwait. Radiation and Environmental Biophysics. 2021;60[4]:639-645. doi:10.1007/s00411-021-00929-3
[67] Yassin MF, Almutairi H, Alhajeri NS, Al-Fadhli FM, Al-Rashidi M, Shatti T. Assessment of noise exposure and associated health risk in school environment. International Journal of Environmental Science and Technology. 2016;13[8]:2011-2024. doi:10.1007/s13762-016-1035-y
[68] Buqammaz M, Gasana J, Alahmad B, Shebl M, Albloushi D. Occupational Noise-Induced Hearing Loss among Migrant Workers in Kuwait. International Journal of Environmental Research and Public Health. 2021;18[10]:5295. doi:10.3390/ijerph18105295
[69] Mohamed IY. Asbestos-cement pneumoconiosis: First surgically confirmed case in kuwait. American Journal of Industrial Medicine. 1990;17[2]:241-245. doi:10.1002/ajim.4700170208
[70] El-Sonbaty, Abdul-Ghaffar NU. Hypokalaemic paralysis precipitated by working in hot weather. Tropical Doctor. 1995;25[2]:64-66. doi:10.1177/004947559502500205
[71] Alahmad B, Al-Hemoud A, Al-Bouwarthan M, et al. Extreme heat and work injuries in Kuwait’s hot summers. Occupational and Environmental Medicine. 2023;80[6]:347-352. doi:10.1136/oemed-2022-108697
[72] Kanan MW. CEMENT DERMATITIS AND ATMOSPHERIC PARAMETERS IN KUWAIT*. British Journal of Dermatology. 1972;86[2]:155-159. doi:10.1111/j.1365-2133.1972.tb16079.x
[73] Al-Shatti AKS, El-Desouky MA, Zaki RM, Al-Azem MA, Al-Lagani M. Health care for pesticide applicators in a locust eradication campaign in Kuwait [1988–1989]. Environmental Research. 1997;73[1-2]:219-226. doi:10.1006/enrs.1997.3735
[74] Al-Ayyadhi N, Akhtar S. Prevalence and Risk Factors Associated with Self-Rated Morbidities Among South Asian Migrant Gas Station Workers in Kuwait. Journal of Immigrant and Minority Health. 2018;20[6]:1324-1331. doi:10.1007/s10903-018-0701-1
[75] Hashim R, Salah A, Mayahi F, Haidary S. Prevalence of postural musculoskeletal symptoms among dental students in United Arab Emirates. BMC Musculoskeletal Disorders. 2021;22[1]. doi:10.1186/s12891-020-03887-x
[76] Al‐Rawi NH, Yousef H, Khamis M, Belkadi O, Ahmed S, Ali S. Vertebral Malalignment among Male Dentists with Workrelated Musculoskeletal Pain in the United Arab Emirates. The Journal of Contemporary Dental Practice. 2018;19[7]:773-777. doi:10.5005/jp-journals-10024-2335
[77] Al‐Rawi NH, Khatib HE, Lin R, et al. Work-related Musculoskeletal Pain among Different Dental Specialists in United Arab Emirates. The Journal of Contemporary Dental Practice. 2016;17[8]:639-644. doi:10.5005/jp-journals-10024-1904
[78] Hussein A, Mando M, Radišauskas R. Work-Related Musculoskeletal Disorders among Dentists in the United Arab Emirates: A Cross-Sectional Study. Medicina-lithuania. 2022;58[12]:1744. doi:10.3390/medicina58121744
[79] Elshami W, Abuzaid MM, Pekkarinen A, Kortesniemi M. ESTIMATION OF OCCUPATIONAL RADIATION EXPOSURE FOR MEDICAL WORKERS IN RADIOLOGY AND CARDIOLOGY IN THE UNITED ARAB EMIRATES: NINE HOSPITALS EXPERIENCE. Radiation Protection Dosimetry. 2020;189[4]:466-474. doi:10.1093/rpd/ncaa060
[80] AlMahmoud T, Elkonaisi I, Grivna M, AlNuaimi G, Abu‐Zidan FM. Eye Injuries and Related Risk Factors among Workers in Small-scale Industrial Enterprises. Ophthalmic Epidemiology. 2020;27[6]:453-459. doi:10.1080/09286586.2020.1770302
[81] Grivna M, Eid HO, Abu‐Zidan FM. Injuries from falling objects in the United Arab Emirates. International Journal of Injury Control and Safety Promotion. 2013;22[1]:68-74. doi:10.1080/17457300.2013.863784
[82] Ahmed H. Noise exposure, awareness, practice and noise annoyance among steel workers in United Arab Emirates. The Open Public Health Journal. 2012;5[1]:28-35. doi:10.2174/1874944501205010028
[83] Elmehdi HM, Emirates UA. Noise levels in UAE Dental clinics: Health impact on dental healthcare professionals. Public Health Frontier. Published online December 24, 2013:189-192. doi:10.5963/phf0204002
[84] Nassar M, Islam MS, D’souza S, et al. Tinnitus Prevalence and Associated Factors among Dental Clinicians in the United Arab Emirates. International Journal of Environmental Research and Public Health. 2023;20[2]:1403. doi:10.3390/ijerph20021403
[85] Jacob A, Newson-Smith M, Murphy EA, Steiner M, Dick F. Sharps injuries among health care workers in the United Arab Emirates. Occupational Medicine. 2010;60[5]:395-397. doi:10.1093/occmed/kqq039
[86] Bener A, Almehdi AM, Alwash R, Al-Neamy FRM. A pilot survey of blood lead levels in various types of workers in the United Arab Emirates. Environment International. 2001;27[4]:311-314. doi:10.1016/s0160-4120[01]00061-7
[87] Bener A, Obineche EN, Gillett MPT, Pasha M, Bishawi B. Association between blood levels of lead, blood pressure and risk of diabetes and heart disease in workers. International Archives of Occupational and Environmental Health. 2001;74[5]:375-378. doi:10.1007/s004200100231
[88] Ahmed H, Abdullah AA. Dust Exposure and Respiratory Symptoms among Cement Factory Workers in the United Arab Emirates. Industrial Health. 2012;50[3]:214-222. doi:10.2486/indhealth.ms1320
[89] Gomes J, Lloyd O, Revitt DM, Norman J. Erythrocyte cholinesterase activity levels in desert farm workers. Occupational Medicine. 1997;47[2]:90-94. doi:10.1093/occmed/47.2.90
[90] Abou-Taleb ANM, Musaiger AO, Abdelmoneim RB. Health status of cement workers in the United Arab Emirates. The Journal of the Royal Society of Health. 1995;115[6]:378-381. doi:10.1177/146642409511500610
[91] Lotah HNA, Agarwal AK, Khanam R. Heavy metals in hair and nails as markers of occupational hazard among welders working in United Arab Emirates. Toxicological Research. 2021;38[1]:63-68. doi:10.1007/s43188-021-00091-4
[92] Al-Neamy FRM, Almehdi AM, Alwash R, Pasha MA, Ahmad I, Bener A. Occupational lead exposure and amino acid profiles and liver function tests in industrial workers. International Journal of Environmental Health Research. 2001;11[2]:181-188. doi:10.1080/09603120020047564
[93] Beshwari MMM, Bener A, Ameen A, Almehdi AM, Ouda HZ, Pasha MA. Pesticide-related health problems and diseases among farmers in the United Arab Emirates. International Journal of Environmental Health Research. 1999;9[3]:213-221. doi:10.1080/09603129973182
[94] Al-Neaimi YI, Gomes J, Lloyd O. Respiratory illnesses and ventilatory function among workers at a cement factory in a rapidly developing country. Occupational Medicine. 2001;51[6]:367-373. doi:10.1093/occmed/51.6.367
[95] Bener A, Brebner JA, Atta MNS, Gomes J, Ozkaragoz F, Cheema M. Respiratory symptoms and lung function in taxi drivers and manual workers. Aerobiologia. 1997;13[1]:11-15. doi:10.1007/bf02694785
[96] Bener A, Galadari I, Al-Mutawa JK, Al-Maskari F, Das M, Abuzeid MSO. Respiratory symptoms and lung function in garage workers and taxi drivers. Journal of the Royal Society for the Promotion of Health. 1998;118[6]:346-353. doi:10.1177/146642409811800613
[97] Bener A, Lestringant GG, Beshwari MMM, Pasha MA. Respiratory symptoms, skin disorders and serum IgE levels in farm workers. PubMed. 1999;31[2]:52-56. https://pubmed.ncbi.nlm.nih.gov/10219428
[98] Dafalla AIA, Almuhairi SASO, AlHosani MHJ, et al. Intestinal parasitic infections among expatriate workers in various occupations in Sharjah, United Arab Emirates. Revista Do Instituto De Medicina Tropical De Sao Paulo. 2017;59[0]. doi:10.1590/s1678-9946201759082
[99] Al-Ali K, Hashim R. Occupational health problems of dentists in the United Arab Emirates. International Dental Journal. 2012;62[1]:52-56. doi:10.1111/j.1875-595x.2011.00091.x
[100] Salem AM, Jaumally BA, Bayanzay K, Khoury KE, Torkaman A. Traumatic brain injuries from work accidents: a retrospective study. Occupational Medicine. 2013;63[5]:358-360. doi:10.1093/occmed/kqt037
[101] Ka AW, Almas K, Shethri A, Mq AQ. Back &amp; Neck Problems Among Dentists and Dental Auxiliaries. The Journal of Contemporary Dental Practice. 2001;2[3]:1-10. doi:10.5005/jcdp-2-3-1
[102] Meo SA, Alsaaran ZF, Alshehri M, et al. Work-Related musculoskeletal symptoms among building construction workers in Riyadh, Saudi Arabia. Pakistan Journal of Medical Sciences. 2013;29[6]. doi:10.12669/pjms.296.4052
[103] Behisi M, Al-Otaibi ST, Beach J. Back pain among health care workers in a Saudi Aramco facility: prevalence and associated factors. Archives of Environmental & Occupational Health. 2013;68[1]:30-38. doi:10.1080/19338244.2011.627895
[104] Attar SM. Frequency and risk factors of musculoskeletal pain in nurses at a tertiary centre in Jeddah, Saudi Arabia: a cross sectional study. BMC Research Notes. 2014;7[1]. doi:10.1186/1756-0500-7-61
[105] Alghadir AH, Anwer S. Prevalence of musculoskeletal pain in construction workers in Saudi Arabia. The Scientific World Journal. 2015;2015:1-5. doi:10.1155/2015/529873
[106] Aljanakh M, Shaikh S, Siddiqui AA, Al-Mansour M, Hassan SS. Prevalence of musculoskeletal disorders among dentists in the Ha’il Region of Saudi Arabia. Annals of Saudi Medicine. 2015;35[6]:456-461. doi:10.5144/0256-4947.2015.456
[107] Al-Juhani MAM, Khandekar R, Al-Harby M, Al-Hassan A, Edward DP. Neck and upper back pain among eye care professionals. Occupational Medicine. Published online September 28, 2015:kqv132. doi:10.1093/occmed/kqv132
[108] Ezzat HM, Al-Sultan A, Al-Shammari A, et al. Prevalence of neck pain among cabin crew of Saudi Airlines. Journal of Back and Musculoskeletal Rehabilitation. 2015;28[3]:425-431. doi:10.3233/bmr-140536
[109] Homaid MB, Abdelmoety D, Alshareef W, et al. Prevalence and risk factors of low back pain among operation room staff at a Tertiary Care Center, Makkah, Saudi Arabia: a cross-sectional study. Annals of Occupational and Environmental Medicine. 2016;28[1]. doi:10.1186/s40557-016-0089-0
[110] Muaidi QI, Shanb AA. Prevalence causes and impact of work related musculoskeletal disorders among physical therapists. Journal of Back and Musculoskeletal Rehabilitation. 2016;29[4]:763-769. doi:10.3233/bmr-160687
[111] Al-Mohrej OA, AlShaalan NS, Al-Bani WM, Masuadi E, Almodaimegh H. Prevalence of musculoskeletal pain of the neck, upper extremities and lower back among dental practitioners working in Riyadh, Saudi Arabia: a cross-sectional study. BMJ Open. 2016;6[6]:e011100. doi:10.1136/bmjopen-2016-011100
[112] Alshehri ZY, Al-Zoughool M. Self-reported musculoskeletal symptoms among dentists in Saudi Arabia. Industrial Health. 2017;55[4]:338-344. doi:10.2486/indhealth.2016-0174
[113] Al-Shehri Z, Zoughool MA. RETRACTED ARTICLE: Prevalence and risk factors of musculoskeletal symptoms among dental students and dental practitioners in Riyadh City, Saudi Arabia. Archives of Environmental & Occupational Health. 2017;73[1]:56-63. doi:10.1080/19338244.2017.1299085
[114] El-Helaly M, Balkhy HH, Vallenius L. Carpal tunnel syndrome among laboratory technicians in relation to personal and ergonomic factors at work. Journal of Occupational Health. 2017;59[6]:513-520. doi:10.1539/joh.16-0279-oa
[115] Alghadir AH, Zafar H, Iqbal ZA, Al-Eisa E. Work-Related low back pain among physical therapists in Riyadh, Saudi Arabia. Workplace Health & Safety. 2017;65[8]:337-345. doi:10.1177/2165079916670167
[116] Al-Rammah TY, Aloufi AS, Algaeed SK, Alogail NS. The prevalence of work-related musculoskeletal disorders among sonographers. Work-a Journal of Prevention Assessment & Rehabilitation. 2017;57[2]:211-219. doi:10.3233/wor-172558
[117] Al-Shagga MA, Ibrahim IAA, Shahzad N, Al-Dubai SAR. PREVALENCE AND ASSOCIATED FACTORS OF MUSCOSKELETAL DISORDERS AMONG ARABIC CALLIGRAPHERS LIVING IN SAUDI... Malaysian Journal of Public Health Medicine. 2018;18[2]:39-44.
[118] Al-Salameen AH, Abugad H, Al-Otaibi ST. Low back pain among workers in a paint factory. Saudi Journal of Medicine and Medical Sciences. 2019;7[1]:33. doi:10.4103/sjmms.sjmms_81_17
[119] Alnaami I, Awadalla NJ, Alkhairy MA, et al. Prevalence and factors associated with low back pain among health care workers in southwestern Saudi Arabia. BMC Musculoskeletal Disorders. 2019;20[1]. doi:10.1186/s12891-019-2431-5
[120] Alnefaie M, Alamri AA, Hariri AF, et al. Musculoskeletal Symptoms Among Surgeons at a Tertiary Care Center: a Survey Based Study. Medicinski Arhiv. 2019;73[1]:49. doi:10.5455/medarh.2019.73.49-54
[121] Alhusain F, Almohrij M, Althukeir F, et al. Prevalence of carpal tunnel syndrome symptoms among dentists working in Riyadh. Annals of Saudi Medicine. 2019;39[2]:104-111. doi:10.5144/0256-4947.2019.07.03.1405
[122] Gaowgzeh RA. Low back pain among nursing professionals in Jeddah, Saudi Arabia: Prevalence and risk factors. Journal of Back and Musculoskeletal Rehabilitation. 2019;32[4]:555-560. doi:10.3233/bmr-181218
[123] Abdel-Salam DM, Almuhaisen AS, Alsubiti RA, et al. Musculoskeletal pain and its correlates among secondary school female teachers in Aljouf region, Saudi Arabia. Journal of Public Health. 2019;29[2]:303-310. doi:10.1007/s10389-019-01127-8
[124] Shammari MA, Hassan A, Dandan OA, Gadeeb MA, Bubshait D. Musculoskeletal symptoms among radiologists in Saudi Arabia: a multi-center cross-sectional study. BMC Musculoskeletal Disorders. 2019;20[1]. doi:10.1186/s12891-019-2933-1
[125] Aboalshamat K. Nordic assessment of occupational disorders among dental students and dentists in Saudi Arabia. Journal of International Society of Preventive and Community Dentistry. 2020;10[5]:561. doi:10.4103/jispcd.jispcd_142_20
[126] AlNekhilan AF, AlTamimi AM, Alaqeel BY, AlHawery AA, Alfadhel S, Masuadi E. Work-related musculoskeletal disorders among clinical laboratory workers. Avicenna Journal of Medicine. 2020;10[1]:29. doi:10.4103/ajm.ajm_67_19
[127] Dalboh A, Alshehri NA, Alrafie AA, Bakri KA. Prevalence and awareness of varicose veins among teachers in Abha, Saudi Arabia. Journal of Family Medicine and Primary Care. 2020;9[9]:4784. doi:10.4103/jfmpc.jfmpc_490_20
[128] Tariah HA, Nafai S, Alajmi M, Almutairi FM, Alanazi B. Work-related musculoskeletal disorders in nurses working in the Kingdom of Saudi Arabia. Work-a Journal of Prevention Assessment & Rehabilitation. 2020;65[2]:421-428. doi:10.3233/wor-203094
[129] Algarni FS, Kachanathu SJ, AlAbdulwahab SS. A Cross-Sectional Study on the Association of Patterns and Physical Risk Factors with Musculoskeletal Disorders among Academicians in Saudi Arabia. BioMed Research International. 2020;2020:1-7. doi:10.1155/2020/8930968
[130] Kaleem SM, Asif SM, Kota MZ, Alam T, Assiri H, Zakirulla M. Ergonomic Considerations in the incidence of CTS in College of Dentistry, King Khalid University, Abha - Kingdom of Saudi Arabia. PubMed. 2020;18[1]:277-285. doi:10.3290/j.ohpd.a44031
[131] Elmannan AAA, AlHindi HA, AlBaltan RI, et al. Non-specific low back pain among nurses in Qassim, Saudi Arabia. Cureus. Published online November 15, 2021. doi:10.7759/cureus.19594
[132] Abolfotouh MA, Alomair F, Alangari D, Bushnak I, Aldebasi B, Almansoof AS. Epidemiology of work-related lower back pain among rehabilitation professionals in Saudi Arabia. Eastern Mediterranean Health Journal. 2021;27[4]:390-398. doi:10.26719/emhj.21.019
[133] Althomali OW, Amin J, Alghamdi W, Shaik DH. Prevalence and Factors Associated with Musculoskeletal Disorders among Secondary Schoolteachers in Hail, Saudi Arabia: A Cross-Sectional Survey. International Journal of Environmental Research and Public Health. 2021;18[12]:6632. doi:10.3390/ijerph18126632
[134] Aldukhayel A, Almeathem FK, Aldughayyim AA, et al. Musculoskeletal pain among school teachers in Qassim, Saudi Arabia: prevalence, pattern, and its risk factors. Cureus. Published online August 27, 2021. doi:10.7759/cureus.17510
[135] Aldhafian OR, Alsamari F, Alshahrani NA, et al. Musculoskeletal pain among male faculty members of the College of Medicine and College of Dentistry. Medicine. 2021;100[21]:e26176. doi:10.1097/md.0000000000026176
[136] Almaghrabi A, Alsharif F. Prevalence of Low Back Pain and Associated Risk Factors among Nurses at King Abdulaziz University Hospital. International Journal of Environmental Research and Public Health. 2021;18[4]:1567. doi:10.3390/ijerph18041567
[137] Kakaraparthi VN, Vishwanathan K, Gadhavi B, et al. The prevalence, characteristics, and impact of work-related musculoskeletal disorders among physical therapists in the Kingdom of Saudi Arabia – a cross-sectional study. Medycyna Pracy. Published online August 27, 2021. doi:10.13075/mp.5893.01114
[138] AlOmar RS, AlShamlan NA, Alawashiz S, Badawood Y, Ghwoidi BA, Abugad H. Musculoskeletal symptoms and their associated risk factors among Saudi office workers: a cross-sectional study. BMC Musculoskeletal Disorders. 2021;22[1]. doi:10.1186/s12891-021-04652-4
[139] Alhasan AS, Aalam WA. Magnitude and determinants of computer vision syndrome among radiologists in Saudi Arabia: a national survey. Academic Radiology. 2022;29[9]:e197-e204. doi:10.1016/j.acra.2021.10.023
[140] Alhakami AM, Madkhli A, Ghareeb M, et al. The Prevalence and Associated Factors of Neck Pain among Ministry of Health Office Workers in Saudi Arabia: A Cross Sectional Study. Healthcare. 2022;10[7]:1320. doi:10.3390/healthcare10071320
[141] Althomali OW. Long-Term Prevalence and Risk Factors of Musculoskeletal Disorders among the Schoolteachers in Hail, Saudi Arabia: A Cross-Sectional Study. BioMed Research International. 2022;2022:1-7. doi:10.1155/2022/3610196
[142] Shubayr N, Alashban Y. Musculoskeletal symptoms among radiation technologists in Saudi Arabia: prevalence and causative factors. Acta Radiologica. 2021;63[4]:497-503. doi:10.1177/02841851211004428
[143] Alzayani MK, Salama KF, Zafar M. Work-related musculoskeletal disorders among dental staff in Armed Force Hospital in Dhahran, Saudi Arabia. DOAJ [DOAJ: Directory of Open Access Journals]. 2021;12:119. doi:10.4103/ijpvm.ijpvm_136_20
[144] Ali SA, Najmi WK, Hakami FM, et al. Prevalence of varicose veins among nurses in different departments in Jazan Public Hospitals, Saudi Arabia: a Cross-Sectional study. Cureus. Published online April 25, 2022. doi:10.7759/cureus.24462
[145] AlMaghlouth MK, Alserhani NM, Aldossary FA, Alabdulqader MA, Al-Dhafer BA. Prevalence, patterns, and risk factors of Work-Related Musculoskeletal Diseases among teachers in the Eastern Province, Saudi Arabia: A Community-Based Retrospective Cross-Sectional Survey. Cureus. Published online December 4, 2022. doi:10.7759/cureus.32178
[146] Alghadir AH, Khalid S, Iqbal ZA. Work-related musculoskeletal disorders among information technology professionals in Riyadh, Saudi Arabia. Medycyna Pracy. 2022;73[5]:397-406. doi:10.13075/mp.5893.01281
[147] Alzahrani AS, Baatiyyah EA, Bakry S, et al. The prevalence of back pain among male teachers in Makkah region, Saudi Arabia: An analytic cross-sectional study. Medical Science. 2022;26[125]:1-9. doi:10.54905/disssi/v26i125/ms318e2326
[148] Saikhan LA. Prevalence, characteristics, consequences, and awareness of work-related musculoskeletal pain among cardiac sonographers compared with other healthcare workers in Saudi Arabia: A cross sectional study. PLOS ONE. 2023;18[5]:e0285369. doi:10.1371/journal.pone.0285369
[149] Aldaheri A, Al-Juhani MAM, Aldaheri R. The prevalence and associated factors of lower back pain among surgeons in Makkah Region, Saudi Arabia. Journal of Family Medicine and Primary Care. 2023;12[7]:1308-1314. doi:10.4103/jfmpc.jfmpc_468_22
[150] Alelyani M, Gameraddin M, Khushayl AMA, et al. Work-related musculoskeletal symptoms among Saudi radiologists: a cross-sectional multi-centre study. BMC Musculoskeletal Disorders. 2023;24[1]. doi:10.1186/s12891-023-06596-3
[151] AlSahiem J, Alghamdi S, AlQahtani R, et al. Musculoskeletal disorders among dental students: a survey from Saudi Arabia. BMC Oral Health. 2023;23[1]. doi:10.1186/s12903-023-03469-y
[152] Al-Haj AN, Lobriguito AM, Al-Gain I. Staff eye doses in a large medical centre in Saudi Arabia: are they meeting the new ICRP recommendations? Radiation Protection Dosimetry. 2015;165[1-4]:294-298. doi:10.1093/rpd/ncv073
[153] Salama KF, AlObireed A, AlBagawi M, AlSufayan Y, AlSerheed M. Assessment of occupational radiation exposure among medical staff in health-care facilities in the Eastern Province, Kingdom of Saudi Arabia. Indian Journal of Industrial Medicine. 2016;20[1]:21. doi:10.4103/0019-5278.183832
[154] Alashrah S, El-Taher A. Assessing Exposure Hazards and Metal Analysis Resulting from Bauxite Samples Collected from a Saudi Arabian Mine. Polish Journal of Environmental Studies. 2018;27[3]:959-966. doi:10.15244/pjoes/76177
[155] Alkhorayef M, Sulieman A, Mohamed‐Ahmed MM, et al. Staff and ambient radiation dose resulting from therapeutic nuclear medicine procedures. Applied Radiation and Isotopes. 2018;141:270-274. doi:10.1016/j.apradiso.2018.07.014
[156] Shhub AN. Monitoring radiation exposure of Saudi Aramco pilots. Health Physics. 2019;118[2]:162-169. doi:10.1097/hp.0000000000001162
[157] Alkhorayef M, Mayhoub FH, Salah H, et al. Assessment of occupational exposure and radiation risks in nuclear medicine departments. Radiation Physics and Chemistry. 2020;170:108529. doi:10.1016/j.radphyschem.2019.108529
[158] Shubayr N, Alashban Y, Almalki M, Aldawood S, Aldosari A. Occupational radiation exposure among diagnostic radiology workers in the Saudi ministry of health hospitals and medical centers: A five-year national retrospective study. Journal of King Saud University - Science. 2021;33[1]:101249. doi:10.1016/j.jksus.2020.101249
[159] Alashban Y, Shubayr N, Almalki M, Albeshan S, Aldawood S, Aldosari A. Assessment of radiation dose for dental workers in Saudi Arabia [2015–2019]. Journal of King Saud University - Science. 2021;33[1]:101250. doi:10.1016/j.jksus.2020.101250
[160] Al-Mohammed HI, Sulieman A, Mayhoub FH, et al. Occupational exposure and radiobiological risk from thyroid radioiodine therapy in Saudi Arabia. Scientific Reports. 2021;11[1]. doi:10.1038/s41598-021-93342-1
[161] Johary YH, Aamry A, Albarakati S, et al. Staff radiation exposure at four radiology departments in the Aseer region of Saudi Arabia. Radiation Physics and Chemistry. 2022;200:110302. doi:10.1016/j.radphyschem.2022.110302
[162] Khouqeer GA, Sulieman A, Mayhoub FH, et al. Staff occupational exposure and ambient doses resulting from patients undergoing nuclear medicine procedures’. Radiation Physics and Chemistry. 2023;212:111091. doi:10.1016/j.radphyschem.2023.111091
[163] Omer H, Salah H, Tamam N, et al. Assessment of occupational exposure from PET and PET/CT scanning in Saudi Arabia. Radiation Physics and Chemistry. 2023;204:110642. doi:10.1016/j.radphyschem.2022.110642
[164] Shanks NJ, Al-Kalai D. Occupation risk of needlestick injuries among health care personnel in Saudi Arabia. Journal of Hospital Infection. 1995;29[3]:221-226. doi:10.1016/0195-6701[95]90332-1
[165] Memish ZA, Almuneef M, Dillon JAR. Epidemiology of needlestick and sharps injuries in a tertiary care center in Saudi Arabia. American Journal of Infection Control. 2002;30[4]:234-241. doi:10.1067/mic.2002.118841
[166] Ghamdi SA, Al-Azraqi TA, Bello CSS, Gutierrez H, Hyde MR, Abdullah M. Needlestick and sharps injuries at Asir Central Hospital, Abha, Saudi Arabia. Annals of Saudi Medicine. 2003;23[6]:404-407. doi:10.5144/0256-4947.2003.404
[167] Jahan S. Epidemiology of needlestick injuries among health care workers in a secondary care hospital in Saudi Arabia. Annals of Saudi Medicine. 2005;25[3]:233-238. doi:10.5144/0256-4947.2005.233
[168] Mahfouz AA, Abdelmoneim I, Khan M, et al. Injection safety at primary health care level in south-western Saudi Arabia. Eastern Mediterranean Health Journal. 2009;15[2]:443-450. doi:10.26719/2009.15.2.443
[169] El-Hazmi M, Almajid F. Needle stick and sharps injuries among health care workers: A 5-year surveillance in a teaching center in... ResearchGate. Published online May 1, 2008. https://www.researchgate.net/publication/228639578
[170] Memish ZA, Assiri AM, Eldalatony MM, Hathout H, Alzoman H, Undaya M. Risk analysis of needle stick and sharp object injuries among health care workers in a tertiary care hospital [Saudi Arabia]. Journal of Epidemiology and Global Health. 2013;3[3]:123. doi:10.1016/j.jegh.2013.03.004
[171] Khabour OF, Ali KHA, Mahallawi WH. Occupational infection and needle stick injury among clinical laboratory workers in Al-Madinah city, Saudi Arabia. Journal of Occupational Medicine and Toxicology. 2018;13[1]. doi:10.1186/s12995-018-0198-5
[172] Samargandy S, Bukhari LM, Samargandy S, et al. Epidemiology and clinical consequences of occupational exposure to blood and other body fluids in a university hospital in Saudi Arabia. Saudi Medical Journal. 2016;37[7]:783-790. doi:10.15537/smj.2016.7.14261
[173] Aldakhil L, Yenugadhati N, Al-Seraihi O, Al-Zoughool M. Prevalence and associated factors for needlestick and sharp injuries [NSIs] among dental assistants in Jeddah, Saudi Arabia. Environmental Health and Preventive Medicine. 2019;24[1]. doi:10.1186/s12199-019-0815-7
[174] Makeen AM, Alharbi A, Mahfouz MS, et al. Needlestick and sharps injuries among secondary and tertiary healthcare workers, Saudi Arabia. Nursing Open. 2021;9[1]:816-823. doi:10.1002/nop2.1136
[175] Fadil RA, Abdelmutalab NA, Abdelhafeez SA, et al. Pattern and risk factors of sharp object injuries among health care workers in two tertiary hospitals, Al Taif-Kingdom of Saudi Arabia 2016–2018. Saudi Journal of Biological Sciences. 2021;28[11]:6582-6585. doi:10.1016/j.sjbs.2021.07.031
[176] Abalkhail A, Kabir R, Elmosaad YM, et al. Needle-Stick and Sharp Injuries among Hospital Healthcare Workers in Saudi Arabia: A Cross-Sectional Survey. International Journal of Environmental Research and Public Health. 2022;19[10]:6342. doi:10.3390/ijerph19106342
[177] Alharazi R, Almutary H, Felemban O, et al. Prevalence of needle stick injuries among nurses in Jeddah, Saudi Arabia. Nursing. 2022;Volume 12:235-246. doi:10.2147/nrr.s376343
[178] Al-Shehri S, Kayal M, Almshhad HA, et al. The incidence of needlestick and sharps injuries among healthcare workers in a tertiary care hospital: a Cross-Sectional study. Cureus. Published online April 25, 2023. doi:10.7759/cureus.38097
[179] Ballal SG. Ocular trauma in an iron forging industry in the Eastern Province, Saudi Arabia. Occupational Medicine. 1997;47[2]:77-80. doi:10.1093/occmed/47.2.77
[180] Ali BA, Ballal SG, Albar AA, Ahmed H. Post-shift changes in pulmonary function in a cement factory in eastern Saudi Arabia. Occupational Medicine. 1998;48[8]:519-522. doi:10.1093/occmed/48.8.519
[181] Al-Dawood K. Non-fatal occupational injuries admitted to hospitals among general organization for social insurance workers in Al-Khobar city, Saudi Arabia: Experience of one year. Journal of Family and Community Medicine. 2000;7[2]:35. doi:10.4103/2230-8229.98179
[182] Ahmed H, Dennis J, Badran OB, et al. Occupational noise exposure and hearing loss of workers in two plants in eastern Saudi Arabia. Annals of Occupational Hygiene. Published online July 1, 2001. doi:10.1093/annhyg/45.5.371
[183] Noweir MH, Jamil A. Noise pollution in textile, printing and publishing industries in Saudi Arabia. Environmental Monitoring and Assessment. 2003;83[1]:103-111. doi:10.1023/a:1022418805827
[184] Noweir MH, Bafail AO. Study of summer heat exposure at the ground services operations of a main international airport in Saudi Arabia. Environmental Monitoring and Assessment. 2007;145[1-3]:103-111. doi:10.1007/s10661-007-0019-2
[185] Noweir MH, Zytoon MA. Occupational exposure to noise and hearing thresholds among civilian aircraft maintenance workers. International Journal of Industrial Ergonomics. 2013;43[6]:495-502. doi:10.1016/j.ergon.2013.04.001
[186] Noweir MH, Bafail AO, Jomoah IM. Noise pollution in metalwork and woodwork industries in the Kingdom of Saudi Arabia. International Journal of Occupational Safety and Ergonomics. 2014;20[4]:661-670. doi:10.1080/10803548.2014.11077068
[187] Ballal SG, Ahmed H, Ali BA, Albar AA, Alhasan AY. Pulmonary Effects of Occupational Exposure to Portland Cement: A Study from Eastern Saudi Arabia. International Journal of Occupational and Environmental Health. 2004;10[3]:272-277. doi:10.1179/oeh.2004.10.3.272
[188] Al-Abdulwahhab B, Alduraiby RI, Ahmed MA, et al. Hearing loss and its association with occupational noise exposure among Saudi dentists: a cross-sectional study. BDJ Open. 2016;2[1]. doi:10.1038/bdjopen.2016.6
[189] Alsabaani NA, Awadalla NJ, Saq IHA, et al. Occupational ocular incidents in dentists: a multicentre study in southwestern Saudi Arabia. International Dental Journal. 2017;67[6]:371-377. doi:10.1111/idj.12324
[190] Al-Bouwarthan M, Quinn M, Kriebel D, Wegman DH. Assessment of Heat Stress Exposure among Construction Workers in the Hot Desert Climate of Saudi Arabia. Annals of Work Exposures and Health. 2019;63[5]:505-520. doi:10.1093/annweh/wxz033
[191] Al-Bouwarthan M, Quinn M, Kriebel D, Wegman DH. A field evaluation of construction workers’ activity, hydration status, and heat strain in the extreme summer heat of Saudi Arabia. Annals of Work Exposures and Health. 2020;64[5]:522-535. doi:10.1093/annweh/wxaa029
[192] Al-Bouwarthan M, Quinn M, Kriebel D, Wegman DH. Risk of Kidney Injury among Construction Workers Exposed to Heat Stress: A Longitudinal Study from Saudi Arabia. International Journal of Environmental Research and Public Health. 2020;17[11]:3775. doi:10.3390/ijerph17113775
[193] Eman AS. Prevalence of Noise Induced Hearing Loss and Other Associated Risk Factors among Saudi Dental Professionals. DOAJ [DOAJ: Directory of Open Access Journals]. Published online August 1, 2020. https://doaj.org/article/ad8528475d3c4671978801243dfa53c6
[194] Alsaab F, Alaraifi AK, Alhomaydan WA, Ahmed AZ, Elzubair AG. Hearing impairment in military personnel in Eastern Saudi Arabia. Journal of Family and Community Medicine. 2021;28[2]:110. doi:10.4103/jfcm.jfcm_501_20
[195] Razik M, Altuwayhir A, Almihmadi M, et al. Pattern of traumatic occupational injuries in Saudi Arabia: A cross-sectional study. Journal of Family Medicine and Primary Care. 2022;11[12]:7907. doi:10.4103/jfmpc.jfmpc_2009_22
[196] Ballal SG, Ali B, Albar AA, Ahmed H, Alhasan AY. Bronchial asthma in two chemical fertilizer producing factories in eastern Saudi Arabia. PubMed. 1998;2[4]:330-335. https://pubmed.ncbi.nlm.nih.gov/9559405
[197] Ali BA, Ahmed H, Ballal SG, Albar AA. Pulmonary function of workers exposed to ammonia: a study in the eastern province of Saudi Arabia. International Journal of Occupational and Environmental Health. Published online January 1, 2001. doi:10.1179/107735201800339669
[198] Al‐Sarar AS, Bakr YA, Al-Erimah GS, Hussein HA, Bayoumi AE. Hematological and biochemical alterations in occupationally Pesticides-Exposed workers of Riyadh municipality, Kingdom of Saudi Arabia. Research Journal of Environmental Toxicology. 2009;3[4]:179-185. doi:10.3923/rjet.2009.179.185
[199] Al-Malki AL. Serum heavy metals and hemoglobin related compounds in Saudi Arabia firefighters. Journal of Occupational Medicine and Toxicology. 2009;4[1]:18. doi:10.1186/1745-6673-4-18
[200] Balkhyour MA, Goknil MK. Total Fume and Metal Concentrations during Welding in Selected Factories in Jeddah, Saudi Arabia. International Journal of Environmental Research and Public Health. 2010;7[7]:2978-2987. doi:10.3390/ijerph7072978
[201] El-Helaly M, Balkhy HH, Khan W, Khawaja S. Respiratory symptoms and ventilatory function among health-care workers exposed to cleaning and disinfectant chemicals, a 2-year follow-up study. Toxicology and Industrial Health. 2016;32[12]:2002-2008. doi:10.1177/0748233715610043
[202] Salama KF, Bashawri L. Biochemical and hematological changes among saudi firefighters in the eastern province. International Journal of Environmental Health Engineering. 2017;6[1]:2. doi:10.4103/ijehe.ijehe_12_15
[203] Meo SA, Almutairi FJ, Alasbali MM, et al. Men’s Health in Industries: Plastic Plant Pollution and Prevalence of Pre-diabetes and Type 2 Diabetes Mellitus. American Journal of Men’s Health. 2018;12[6]:2167-2172. doi:10.1177/1557988318800203
[204] Rahmani AH, Al-Hurabi AA, Joseph RJ, Babiker AY. STUDY OF WORK RELATED RESPIRATORY SYMPTOMS AMONG WELDING WORKERS. Asian Journal of Pharmaceutical and Clinical Research. 2018;11[2]:97. doi:10.22159/ajpcr.2018.v11i2.22767
[205] Carrieri M, Pigini D, Martinelli A, et al. Effect of benzene exposure on the urinary biomarkers of nucleic acid oxidation in two cohorts of gasoline pump attendants. International Journal of Environmental Research and Public Health. 2019;16[1]:129. doi:10.3390/ijerph16010129
[206] Shaik AP, Alsaeed AH, Faiyaz‐Ul‐Haque M, Alsaeed MA, Shaik AS. Research Article Human Serum Paraoxonase [PON-1] and hemochromatosis gene [HFE] gene polymorphisms in occupationally exposed lead workers from Saudi Arabia. Genetics and Molecular Research. 2019;18[2]. doi:10.4238/gmr18317
[207] Aljohaney AA, Daali SM, Al-Juaid H, Ageel M, Sukkar S, Alhejaili F. RESPIRATORY SYMPTOMS AND PULMONARY FUNCTION TEST AMONG SALON EMPLOYEES IN JEDDAH, SAUDI ARABIA: CROSS-SECTIONAL STUDY. Chest. 2018;154[4]:791A. doi:10.1016/j.chest.2018.08.712
[208] Ahmad MS. Work Related Respiratory Complications among the Worker of Qassim Cement Industry of Qassim Region, Saudi Arabia. https://www.jrmds.in/abstract/work-related-respiratory-complications-among-the-worker-of-qassim-cement-industry-of-qassim-region-saudi-arabia-44604.html
[209] Sa M, Yab M, Na B, Ma A, Rf H, As A. Prevalence of Pre Diabetes and Type 2 Diabetes Mellitus among cement industry workers. Pakistan Journal of Medical Sciences. 2019;36[2]. doi:10.12669/pjms.36.2.1266
[210] Meo SA, Al-Khlaiwi T, Abukhalaf AA, et al. The Nexus between Workplace Exposure for Wood, Welding, Motor Mechanic, and Oil Refinery Workers and the Prevalence of Prediabetes and Type 2 Diabetes Mellitus. International Journal of Environmental Research and Public Health. 2020;17[11]:3992. doi:10.3390/ijerph17113992
[211] Ahmad I, Balkhyour MA. Occupational exposure and respiratory health of workers at small scale industries. Saudi Journal of Biological Sciences. 2020;27[3]:985-990. doi:10.1016/j.sjbs.2020.01.019
[212] Barradah RK, Ahmad MS, Shaik RA, Ahmad R, Al-Mutairi A, Alghuyaythat WK. Assessment of hand or foot eczema and contact dermatitis among car mechanics. DOAJ [DOAJ: Directory of Open Access Journals]. 2021;25[10]:3737-3744. doi:10.26355/eurrev_202105_25941
[213] Khathlan NA, Al‐dabbus Z, Al‐khdir N, Al‐Matar M, Al‐Nusaif S, Yami BA. Incense [bakhour] smoke exposure is associated with respiratory symptoms and impaired lung function among adults: A cross‐sectional study in Eastern Province of Saudi Arabia. Indoor Air. 2021;31[5]:1577-1582. doi:10.1111/ina.12833
[214] Al-Khlaiwi T, Meo SA, Habib SS, Meo IMU, Alqhtani MS. Incense Burning Indoor Pollution: Impact on the prevalence of prediabetes and Type-2 Diabetes Mellitus. Pakistan Journal of Medical Sciences. 2022;38[7]. doi:10.12669/pjms.38.7.6189
[215] Adly HM, Saleh SA. The association of increased oxidative stress and tumor biomarkers related to polyaromatic hydrocarbons exposure for different occupational workers in Makkah, Saudi Arabia. Cureus. Published online December 26, 2022. doi:10.7759/cureus.32981
[216] Mergler D, Vézina N, Beauvais A. Warts among workers in poultry slaughterhouses. PubMed. 1982;8 Suppl 1:180-184. https://pubmed.ncbi.nlm.nih.gov/7100850
[217] Ahmad S. The prevalence of Staphylococcus aureus colonization among healthcare workers at a specialist hospital in... Journal of Clinical and Diagnostic Research. 2010;4[3]:2438-2441. http://www.jcdr.net/back_issues.asp?issn=0973-709x&year=2010
[218] Mazi W, Senok A, Assiri AM, Kazem N, Abato AT. Occupational exposure to Blood-Borne pathogens in a tertiary hospital. Asia Pacific Journal of Public Health. 2012;27[2]:NP1727-NP1732. doi:10.1177/1010539512450608
[219] Balkhy HH, Miller TL, Ali S, et al. Compliance with Postexposure Screening and Treatment of Latent Tuberculosis Infection among Healthcare Workers in a Tertiary Care Hospital in Saudi Arabia. Infection Control and Hospital Epidemiology. 2014;35[2]:176-181. doi:10.1086/674855
[220] Yezli S, Alotaibi BM, Al-Abdely HM, et al. Acquisition of respiratory and gastrointestinal pathogens among health care workers during the 2015 Hajj season. American Journal of Infection Control. 2019;47[9]:1071-1076. doi:10.1016/j.ajic.2019.02.033
[221] Almasri M, Ahmed QA, Turkestani A, Memish ZA. Hajj abattoirs in Makkah: risk of zoonotic infections among occupational workers. Veterinary Medicine and Science. 2019;5[3]:428-434. doi:10.1002/vms3.169
